# Olverembatinib, a multikinase inhibitor that modulates lipid metabolism, in advanced succinate dehydrogenase-deficient gastrointestinal stromal tumors: a phase 1b study and translational research

**DOI:** 10.1038/s41392-025-02456-9

**Published:** 2025-11-04

**Authors:** Hai-Bo Qiu, Zhiyan Liang, Jing Yang, Ye Zhou, Zhi-Wei Zhou, Xiang-Bin Wan, Ning Li, Kai-Xiong Tao, Yong Li, Xin Wu, Chen Yang, Zi Chen, Hengbang Wang, Lichuang Men, Yan Xiong, Lihui Liu, Dajun Yang, Yifan Zhai, Rui-Hua Xu

**Affiliations:** 1 Department of Gastric Surgery, State Key Laboratory of Oncology in South China, Collaborative Innovation Center for Cancer Medicine, Sun Yat-sen University Cancer Center, Guangzhou, Guangdong China; 2 Ascentage Pharma Group Inc., Rockville, MD USA; 3 State Key Laboratory of Oncology in South China, Collaborative Innovation Center for Cancer Medicine, Sun Yat-sen University Cancer Center, Guangzhou, China; 4 Department of Gastric Surgery, Fudan University Shanghai Cancer Center, Shanghai, China; 5 Department of Oncology, Shanghai Medical College, Fudan University, Shanghai, China; 6 Department of General Surgery, Affiliated Cancer Hospital of Zhengzhou University & Henan Cancer Hospital, Zhengzhou, China; 7 Department of Gastroenterology, Affiliated Cancer Hospital of Zhengzhou University & Henan Cancer Hospital, Zhengzhou, China; 8 Department of Gastrointestinal Surgery, Union Hospital, Tongji Medical College, Huazhong University of Science and Technology, Wuhan, China; 9 Department of Gastrointestinal Surgery, Department of General Surgery, Guangdong Provincial People’s Hospital, Guangdong Academy of Medical Sciences, Guangzhou, China; 10 Department of General Surgical Medicine, The First Medical Center of the People’s Liberation Army, Beijing, China; 11 Guangzhou Healthquest Pharma Co. Ltd., Guangzhou, China; 12 Department of Experimental Research, State Key Laboratory of Oncology in South China Collaborative Innovation Center for Cancer Medicine, Sun Yat-sen University Cancer Center, Guangzhou, China; 13 Department of Medical Oncology, Sun Yat-sen University Cancer Center, State Key Laboratory of Oncology in South China, Guangdong Provincial Clinical Research Center for Cancer, Sun Yat-sen University, Guangzhou, China; 14 Research Unit of Precision Diagnosis and Treatment for Gastrointestinal Cancer, Chinese Academy of Medical Sciences, Guangzhou, China

**Keywords:** Sarcoma, Gastrointestinal cancer, Molecular medicine

## Abstract

Succinate dehydrogenase (SDH)-deficient gastrointestinal stromal tumors (GISTs) are generally resistant to targeted therapy with tyrosine kinase inhibitors (TKIs), such as imatinib, and there are no standard therapeutic options for advanced SDH-deficient GISTs. The precise oncogenic mechanisms of SDH mutations in GIST have not been elucidated. Olverembatinib, a novel multikinase inhibitor, has shown promising activity in treating imatinib-resistant GIST. We conducted a phase 1 study (NCT03594422) to evaluate the safety and antitumor activity of olverembatinib in 66 patients with unresectable/metastatic GIST/other solid tumors, including 26 with TKI-failed SDH-deficient GISTs. To our knowledge, this is the largest prospective clinical trial for this rare GIST subtype. The median follow-up was 14.5 (0.9–57.5) months. Olverembatinib was well tolerated; treatment-emergent adverse events (≥20%) included increases in hepatic transaminases, increases in leukocytes and neutrophils, anemia, and pyrexia. For SDH-deficient GISTs, confirmed partial responses were observed in 6 of the 26 evaluable patients (objective response rate, 23.1%; 95% CI, 9–43.7); an additional 16 (61.5%) did not progress during the first 6 months of treatment. This resulted in a clinical benefit rate of 84.6% (95% CI, 65.1–95.6), and the median progression-free survival was 25.7 months (95% CI, 12.9–NR). As a putative mechanism of action, translational research revealed significant lipid enrichment with the overexpression of lipid uptake-related genes and proteins, including CD36, fatty acid binding proteins, fatty acid transport proteins, and lipid metabolites, in SDH-deficient GIST patients, and olverembatinib suppressed lipid uptake and CD36 expression in GIST cells. Olverembatinib also exerts antitumor effects by inhibiting tumorigenic signaling pathways associated with hypoxia, angiogenesis, proliferation, and survival.

## Introduction

Gastrointestinal stromal tumors (GISTs) are the most common mesenchymal malignancies of the digestive tract.^1^ The incidence is estimated at 10 to 15 cases per million people per year, representing 1% to 3% of all gastrointestinal cancers.^2^ Approximately 75% to 80% of patients have activating mutations in the *KIT* proto-oncogene (CD117). A further 5% to 10% of GISTs have mutations in the platelet-derived growth factor receptor alpha (*PDGFRA*) gene.^3^ Approximately 15% of GISTs lack mutations in KIT/PDGFRA and are designated as KIT/PDGFRA wild-type (WT) GISTs, of which succinate dehydrogenase (SDH)-deficient GISTs are the most common, accounting for 5% to 7.5% of all GISTs.^4^ Succinate dehydrogenase (succinate-coenzyme Q reductase or mitochondrial complex II) is composed of four subunit proteins (SDHA, SDHB, SDHC, and SDHD), which are localized in the inner mitochondrial membrane and plays an integral role in the respiratory chain and cellular metabolism. Dysfunction of SDH complex can be caused by loss-of-function germline or somatic mutation in any of the constituent subunits, or by hypermethylation of the *SDHC* promoter.^5–7^ Consequently, lack of activity of SDH fails to convert succinate to fumarate and leads to an accumulation of succinate. Succinate is an “oncometabolite” that promotes tumorigenesis by inducing DNA hypermethylation^8^ and inhibiting prolyl hydroxylase, thus upregulating hypoxia-inducible factor 1a, which transcriptionally activates hypoxia-induced signaling, including vascular endothelial growth factor receptor (VEGFR) and insulin growth factor receptor (IGF1R).^9^ In addition, studies attemptting to unravel the molecular mechanism of SDH-deficient GISTs found that KIT and FGF receptor 1 (FGFR1) are the most highly expressed receptor tyrosine kinases (RTKs) in this type of cancer. Furthermore, FGF3 and FGF4 were expressed at remarkably high levels in SDH-deficient GISTs.^10^ These mechanisms may set the basis for clinical use of regorafenib and sunitinib, TKIs that target VEGFR and IGF1R, or investigation of the efficacy of FGFR inhibitors in clinical trials in SDH-deficient GISTs.^11^

SDH-deficient GISTs occur primarily in the pediatric and young adult population and in patients with the heritable Carney–Stratakis syndrome or the nonhereditary Carney triad.^12^ Because of their rarity and resistance to conventional therapies, clinical experience in managing SDH-deficient GISTs remains very limited. Furthermore, much of the published data on the efficacy of tyrosine kinase inhibitors (TKIs) in KIT/PDGFRA wild-type GISTs or in pediatric GISTs lack specific annotation of SDH deficiency. In brief, objective tumor responses in this specific GIST subtype are uncommon, and previous experience with imatinib, sunitinib, and regorafenib has shown limited response or disease stabilization in SDH-deficient GISTs.^6,13,14^ In a phase 3 trial examining imatinib in advanced GIST patients, the time to tumor progression was shorter for patients with WT GISTs than for those with exon 11 mutation-positive tumors.^15^ In adolescents with advanced GIST after imatinib failure, treatment with sunitinib resulted in 56% of patients experiencing stable disease for 6 months or more.^16^ In a phase 1/2 prospective trial examining sunitinib in patients aged 6 to less than 18 years with advanced GIST, no tumor response was observed in 6 patients (5 with SDH-deficient tumors and 1 not tested), but 3 patients had stable disease, and the median PFS was only 5.8 months.^17^ In a multicenter phase 2 trial of regorafenib in patients with metastatic and/or unresectable GISTs after failure of standard TKI therapy, the median PFS in 6 patients with SDH-deficient GISTs receiving regorafenib was 10 months, with all patients deriving clinical benefit for more than 16 weeks.^18^ In a phase 2, single-arm trial of regorafenib in the first-line setting for patients with metastatic and/or unresectable *KIT*/*PDGFR* WT GISTs, 15 patients were enrolled, and 2 (13%) patients experienced partial response, the disease control rate at 12 weeks was 86.7%, and the median PFS was 11 months.^19^ Currently, there are no standard-of-care therapeutic options for patients with advanced SDH-deficient GISTs.

Olverembatinib (HQP1351) is a novel TKI that targets multiple kinases, such as BCR-ABL1, KIT, FGFR1-4, SRC, PDGFR, and VEGFR2, and has demonstrated potent efficacy in chronic myeloid leukemia (CML).^20^ Olverembatinib also exhibited potent and superior in vitro and in vivo antitumor activity compared with other TKIs in a preclinical model of imatinib-resistant GIST.^21^

Here, we report the safety and tolerability of olverembatinib in patients with GISTs or other solid tumors and its antitumor activity in patients with SDH-deficient GISTs in a multicenter, open-label, phase 1b randomized clinical trial. This is the largest prospective clinical trial in SDH-deficient GISTs to date. In addition, we report the results of translational research in an attempt to unravel the mechanisms of action (MOAs) of olverembatinib in patients with SDH-deficient GIST, which included clinical biomarker analysis, in vitro and ex vivo studies in several other SDH-deficient tumor cell lines (due to the paucity of preclinical models of SDH-deficient GIST), and primary SDH-deficient GIST cells freshly isolated from patient biopsies. Compared with reported mechanisms of other TKIs in SDH-deficient GISTs, we focused on delineating the potential roles that olverembatinib might play in the cellular metabolism, as SDH deficiency is the pathogenesis of this disease.

## Results

### Patients

**Fig. 1:**
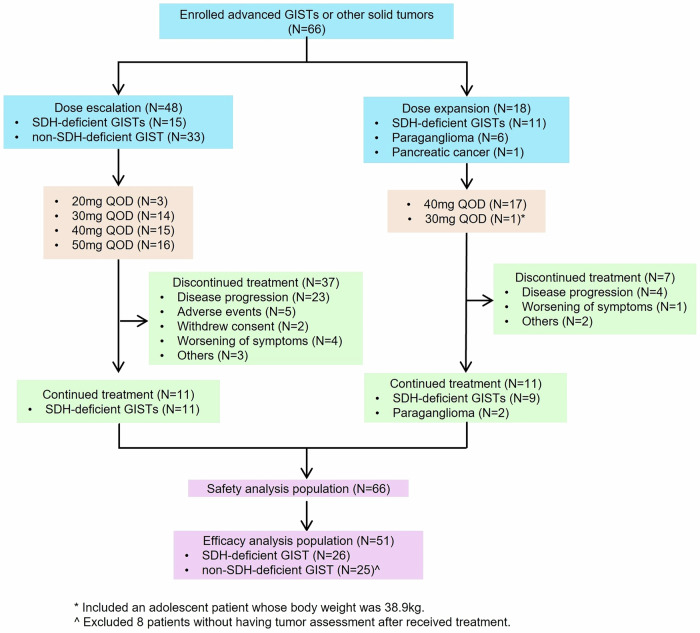
Study design and patient disposition

From July 11, 2018, to December 27, 2023, a total of 66 patients were enrolled, including 26 with SDH-deficient GISTs, 33 with non-SDH-deficient GISTs, 6 with paragangliomas, and 1 with pancreatic cancer (Fig. 1). The baseline characteristics of patients in the non-SDH-deficient and SDH-deficient GIST groups are presented in Supplementary Table 1. In the SDH-deficient GIST group (*n* = 26), the median (range) age was 30 (13–56) years; 7 (26.9%) patients were male; and for most patients (*n* = 24 [92.3%]), the primary tumor site was the stomach. Most patients had been treated with TKIs, including four (15.4%) who received at least two, 11 (42.3%) who received three, and two (7.7%) who received four or more. Among the 26 patients with SDH-deficient GISTs, germline *SDHB* was the most common SDH mutation (*n* = 12 [46.2%]). One patient each had germline *SDHA* and *SDHC* mutations, and the remaining 12 (46.2%) did not have detectable *SDH* mutations. All the SDH-deficient GISTs (*n* = 26) had wild-type *KIT* and wild-type *PDGFRA*, and exons of all the genes, including *KIT*, *PDGFRA*, *SDHA*, *SDHB*, *SDHC* and *SDHD*, were sequenced.

### Safety

At the data cutoff, the median (range) duration of follow-up was 14.5 (0.9–57.5) months. All 66 (100.0%) subjects experienced treatment-emergent AEs (TEAEs), most of which were grade 1 or 2. TEAEs with an incidence of ≥20% included increased leukocyte count (*n* = 32 [48.5%]); anemia (*n* = 31 [47.0%]); increased aspartate aminotransferase (*n* = 31 [47%]); pyrexia (*n* = 28 [42.4%]); constipation (*n* = 27 [40.9%]); increased alanine aminotransferase (*n* = 25 [37.9%]); increased neutrophil count (*n* = 25 [37.9%]); and fatigue (*n* = 20 [30.3%]) (Table 1). Sixty-two (93.9%) patients experienced treatment-related AEs (TRAEs), most of which were grade 1 or 2 (Supplementary Table 2). Nine (13.6%) patients experienced grade ≥3 TRAEs, and five (7.6%) treatment-related serious AEs were reported (Supplementary Table 3).

**Table 1:** Treatment-emergent adverse events (TEAEs) in all patients

	Olverembatinib 20 mg (*n* = 3)		Olverembatinib 30 mg (*n* = 15)		Olverembatinib 40 mg (*n* = 32)		Olverembatinib 50 mg (*n* = 16)		Total (*n* = 66)	
	Any grade	Grade 3-5	Any grade	Grade 3–5	Any grade	Grade 3–5	Any grade	Grade 3–5	Any grade	Grade 3–5
TEAEs, *n* (%)	3 (100.0)	0	15 (100.0)	5 (33.3)	32 (100.0)	12 (37.5)	16 (100.0)	9 (56.3)	66 (100.0)	26 (39.4)
Terms reported in ≥20% of patients, *n* (%)										
Leukocyte count increased	1 (33.3)	0	8 (53.3)	0	15 (46.9)	0	8 (50.0)	0	32 (48.5)	0
Anemia	1 (33.3)	0	8 (53.3)	1 (6.7)	14 (43.8)	2 (6.3)	8 (50.0)	1 (6.3)	31 (47.0)	4 (6.1)
Aspartate aminotransferase increased	0	0	7 (46.7)	0	14 (43.8)	0	10 (62.5)	2 (12.5)	31 (47.0)	2 (3.0)
Hyperuricemia	2 (66.7)	0	7 (46.7)	1 (6.7)	17 (53.1)	0	5 (31.3)	0	31 (47.0)	1 (1.5)
Pyrexia	0	0	5 (33.3)	0	17 (53.1)	0	6 (37.5)	0	28 (42.4)	0
Constipation	2 (66.7)	0	5 (33.3)	0	13 (40.6)	0	7 (43.8)	0	27 (40.9)	0
Alanine aminotransferase increased	0	0	6 (40.0)	0	13 (40.6)	0	6 (37.5)	2 (12.5)	25 (37.9)	2 (3.0)
Neutrophil count increased	1 (33.3)	0	6 (40.0)	0	12 (37.5)	0	6 (37.5)	0	25 (37.9)	0
Fatigue	0	0	4 (26.7)	0	11 (34.4)	1 (3.1)	5 (31.3)	0	20 (30.3)	1 (1.5)
Melanocytic nevus	0	0	3 (20.0)	0	10 (31.3)	0	4 (25.0)	0	17 (25.8)	0
Abdominal pain	0	0	5 (33.3)	1 (6.7)	7 (21.9)	0	3 (18.8)	0	15 (22.7)	1 (1.5)
C-reactive protein increased	0	0	3 (20.0)	0	8 (25.0)	0	4 (25.0)	0	15 (22.7)	0
Hypoalbuminemia	0	0	4 (26.7)	0	5 (15.6)	0	5 (31.3)	0	14 (21.2)	0

### Efficacy

**Fig. 2:**
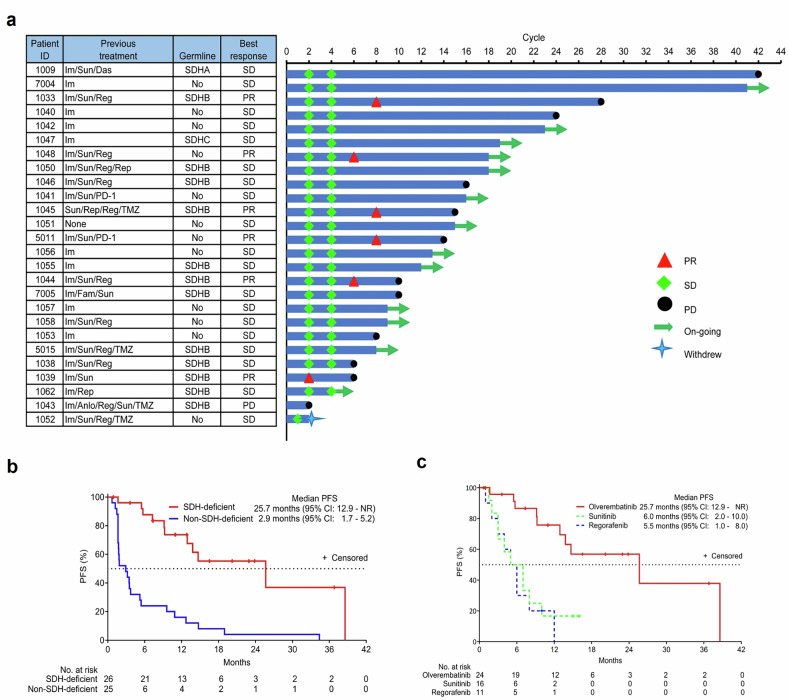
Antitumor efficacy of olverembatinib in patients with SDH-deficient GISTs. **a** Duration of treatment and best response (RECIST v1.1). Each row represents one patient’s duration of treatment with her/his best response. The three left columns show the treatment history, gene mutation, and dose received by each patient. (Im, imatinib; Sun, sunitinib; Das, dasatinib; Reg, regorafenib; Rip, ripretinib; Anlo, anlotinib; Fam, famitinib; TMZ, temozolomide; PD-1, monoclonal antibody targeting PD-1. PD, progressive disease; PR, partial response; SD, stable disease. **b** Kaplan‒Meier estimates of progression-free survival (PFS) by RECIST v1.1. The median PFS was 25.7 months (range, 12.9 to not reached) for olverembatinib in SDH-deficient patients (indicated by the red solid line) and 2.9 months (range, 1.7–5.2) in non-SDH-deficient patients (indicated by the purple solid line). **c** Median (range) PFS was 6.0 (2.0–10.0) and 5.5 (1.0–8.0) months for sunitinib and regorafenib, respectively (indicated by the green and blue dotted lines)

In the SDH-deficient GIST group, with a median (range) follow-up of 17.0 (4.1–57.5) months, 6 of 26 (23.1%) evaluable patients experienced a confirmed PR per RECIST v1.1 (objective response rate, 23.1%; 95% confidence interval [CI], 9–43.7); an additional 16 patients (61.5%) did not progress during the first 6 months of treatment. Together, the clinical benefit rate was 84.6% (95% CI, 65.1–95.6) (Fig. 2a and Supplementary Fig. 1). In the non-SDH-deficient GIST group, no response was observed among the 25 evaluable patients. The median (range) PFS was 25.7 (12.9 to not reached) months among patients with SDH-deficient GISTs and 2.9 (1.7–5.2) months among those with non-SDH-deficient GISTs (Fig. 2b). At the time of data cutoff, all patients with SDH-deficient GISTs survived, resulting in an OS rate of 100%.

To account for available historical control data and ascertain the relative efficacy of olverembatinib, we analyzed the medical histories of the 26 patients with SDH-deficient GISTs enrolled in this study. The median PFS was 6.0 months for patients treated with sunitinib and 5.5 months for those treated with regorafenib, regardless of the treatment line (Fig. 2c). For each treatment line, the median PFS was 6.0 months for first-line TKI treatment, 8.0 months for second-line TKI treatment, and 5.0 months for third-line TKI treatment (Supplementary Table 4).

### Pharmacokinetic analyses

The pharmacokinetic profile of olverembatinib was evaluated in patients with GIST following oral administration at assigned doses (20 to 50 mg QOD) under fed conditions. The results of the pharmacokinetic analysis of olverembatinib at steady state (Cycle 1 Day 27) are summarized in Supplementary Table 5. After oral administration at doses ranging from 20 to 50 mg, the peak concentration of olverembatinib occurred between 6 and 8 h. The mean *t*_1/2_ ranged from 18.9–27 h. Olverembatinib exhibited an approximately dose-proportional increase in *C*_max_ and AUC_last_ over the dose range of 20 to 50 mg. There was no significant accumulation of olverembatinib after multiple-dose administration.

### Biomarker analysis

RNA sequencing was performed via FFPE tumor tissue samples from 7 available patients, three of whom achieved a PR and four of whom achieved SD (Supplementary Table 6). In patients with PR, Kyoto Encyclopedia of Genes and Genomes (KEGG) enrichment analysis (Supplementary Fig. 2a) revealed that the upregulated genes were enriched mainly in the metabolism of fatty acids/lipids, glucose, and amino acids.

In addition, GO enrichment analysis was performed to determine the main biological functions of these upregulated genes. Supplementary Fig. 2b shows that most of the differentially expressed genes (DEGs) were associated with the catabolic processes of fatty acids/lipids, glucose, and amino acids.

Moreover, gene set enrichment analysis (GSEA) revealed that hypoxia and the HIF-1 transcription network pathways were enriched in patients who experienced a PR (Supplementary Fig. 2c), indicating that olverembatinib might exert its antitumor effects on SDH-deficient GISTs by targeting the HIF pathway, which affects tumor cell metabolism.

### Mechanism of action studies

#### Olverembatinib shows superior antiproliferative activity in SDH-deficient tumor cell lines and primary SDH-deficient GIST cells

To elucidate the effects of olverembatinib on SDH-deficient GISTs, we performed preclinical research using SDHB-deficient models or ex vivo studies involving human samples. There is no immortalized SDH-deficient GIST cell line commercially available, and the currently established GIST cell lines with mutated *KIT* or *PDGFRA* cannot be used because olverembatinib is a potent inhibitor of these targets. Consequently, other types of SDH-deficient tumor cell lines were used to assess the in vitro antitumor activity of olverembatinib. These included Jurkat clone E6-1 (*SDHB* mutation [mut]), OS-RC-2 (*SDHB* mut), RKO (*SDHA* mut), and rat pheochromocytoma PC12 cells in which SDHB was knocked down (KD, PC12#5F7). As shown in Supplementary Table 7, olverembatinib exhibited superior antiproliferative activity in SDH-deficient cell lines compared with other TKIs, including imatinib, sunitinib, regorafenib, and ripretinib. In addition, we obtained primary cell samples freshly isolated from the tumor specimens of four patients with SDH-deficient GISTs (patients #1, 2, 3, and 4). The SDH deficiency status of these patients was confirmed by *SDH* gene mutation (Supplementary Table 8) and SDHB protein expression (Supplementary Fig. 3). The antiproliferative activities of olverembatinib and other TKIs were evaluated in freshly isolated primary cells from three patients with SDH-deficient GISTs. As shown in Supplementary Fig. 4 and Supplementary Table 9, olverembatinib was the most potent inhibitor among all the TKIs tested under the same experimental conditions.

#### Multi-omics analyses reveal a deregulated lipid metabolism in tumor tissues from patients with SDH-deficient GIST

**Fig. 3:**
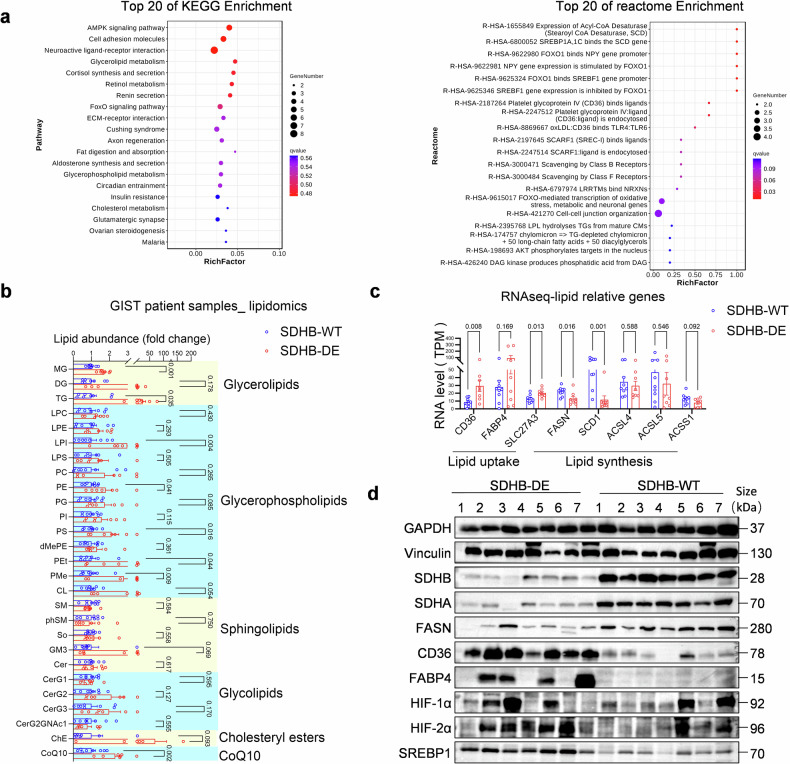
Differences in lipid metabolism among SDH-deficient GISTs. **a** The Kyoto Encyclopedia of Genes and Genomes (KEGG) (left) and Reactome (right) enrichment bubble plots indicate enrichment of the pathways of the differentially expressed genes in SDH-deficient (*n* = 8) and non-SDH-deficient (*n* = 9) GIST tumor tissues. Both plots utilize the top 20 pathways with the smallest *q*-values for construction; the vertical coordinate lists the pathway; the horizontal coordinate represents the enrichment factor (the number of differentially expressed genes divided by the total number of genes in the pathway); the size indicates the number of differentially expressed genes in the pathway; and the redder the color is, the smaller the *q* value. **b** Lipidomic data comparing the lipid abundance of SDH-deficient (*n* = 7) and non-SDH-deficient GIST (*n* = 8) tumor tissues. **c** Gene expression levels related to lipid uptake and lipid synthesis in SDH-deficient (*n* = 8) and non-SDH-deficient (*n* = 9) GIST tumor tissues. **d** Protein expression levels in SDH-deficient (*n* = 7) and non-SDH-deficient (*n* = 7) GIST tumor tissues confirmed the results obtained from RNAseq

To explore potential MOAs of olverembatinib in SDH-deficient GISTs, we characterized their biological features and compared them for patients with SDH-deficient vs. non-SDH-deficient GISTs. Tumor tissues from 17 patients with GIST treated at Sun Yat-sen University Cancer Center were collected, including 8 with SDH-deficient GIST and 9 with non-SDH-deficient GISTs. Tissues from these patients were evaluated by RNA sequencing and untargeted metabolomics analyses. After the KEGG database^22^ was used to enrich pathways for the DEGs between the SDH-deficient and non-SDH-deficient groups (Fig. 3a, left), lipid metabolism pathways were found to be enriched. These included glycerolipid metabolism, fat digestion and absorption, glycerophospholipid metabolism, and cholesterol metabolism. Furthermore, we used the Reactome database^23,24^ to enrich pathways of DEGs between two sets of samples (Fig. 3a, right). The data revealed that lipid metabolism-related genes, including stearoyl-CoA desaturase 1 (*SCD1*), sterol regulatory element-binding protein (*SREBP1*), and CD36 (platelet glycoprotein IV), were enriched, which aligns with the results of biomarker analyses showing enriched genes linked to the catabolic processes of fatty acids/lipids in patients with PR after olverembatinib treatment (Supplementary Fig. 2b).

For metabolomics analysis, lipids from SDH-deficient and non-SDH-deficient tumor tissues were extracted, and metabolites were detected via liquid chromatography‒mass spectrometry (LC‒MS) (Fig. 3b). The levels of glycerolipids (GLs), including mono esters, diglycerides, and triglycerides, were significantly greater in the SDH deficient-group than in the non-SDH-deficient group. In addition, sphingolipids (SPs), glycolipids, cholesteryl esters, CoQ10, and most glycerophospholipids (GPLsPPs) were also elevated. When the mRNA levels of genes related to lipid metabolism were compared (Fig. 3c), upregulated expression of genes related to lipid uptake, including *CD36*, fatty acid-binding protein 4 (*FABP4*), and solute carrier family 22 member 3 *(SLC27A3)*, was observed in the SDH-deficient tumors. On the other hand, the expression of genes related to lipid synthesis, including fatty acid synthase (*FASN*), stearoyl-CoA desaturase 1 (*SCD1*), acyl-CoA synthetase long chain family member (*ACSL4, ACSL5*), and acyl-CoA synthetase 1 (*ACSS1*), was downregulated. These transcriptional differences were further confirmed at the protein level via western blotting analysis of CD36, FABP4 and FASN. In addition, western blotting revealed altered protein levels of key lipid metabolism-related transcription factors, including HIF-1α, HIF-2α, and SREBP1 (Fig. 3d).

On the basis of these findings, we hypothesize that SDH-deficient GIST cells are more dependent on fatty acid uptake from the tumor microenvironment than non-SDH-deficient GIST cells are.

#### Lipid uptake is increased in SDH-deficient GIST cells compared with non-SDH-deficient GIST cells and can be inhibited by olverembatinib through CD36 targeting

**Fig. 4:**
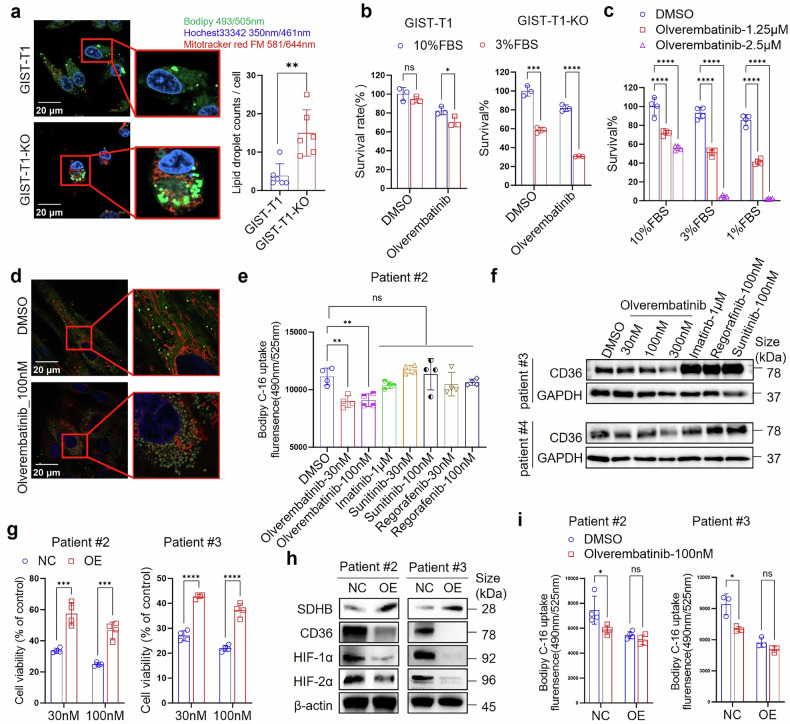
SDHB knockout enhanced lipid uptake in GIST cells, which could be inhibited by olverembatinib treatment, and reoverexpression of SDHB increased the resistance of these cells to olverembatinib and abolished the suppression of lipid uptake by olverembatinib. **a** SDHB knockout enhanced lipid accumulation in GIST-T1 cells. Representative images showing lipid droplets stained with BODIPY-labeled GIST-T1 cells and GIST-T1-SDHB-KO cells. Lipid droplet counts per cell were then measured via ImageJ according to the fluorescence image (*n* = 6). **b** Compared with GIST-T1 cells, GIST-T1-SDHB-KO cells were more sensitive to olverembatinib treatment in lipid-depleted medium. GIST-T1 cells and GIST-T1-SDHB-KO cells were treated with 3 nM olverembatinib in RPMI 1640 with 10% or 3% FBS, and then, cell viability was measured with a CellTiter 96^®^ Aqueous Non-Radioactive Cell Proliferation Assay. **c** SDH-deficient GIST primary cells cultured in lipid-depleted medium were more sensitive to olverembatinib treatment than those cultured in lipid-rich medium. SDH-deficient GIST primary cells from Patient #1 were treated with 1.25 and 2.5 μM olverembatinib in RPMI 1640 with 10%, 3% and 1% FBS, and then, cell viability was measured with a CellTiter 96^®^ Aqueous Non-Radioactive Cell Proliferation Assay. **d** Olverembatinib inhibited lipid droplet accumulation and lipid uptake in SDH-deficient GIST primary cells. Representative images showing lipid droplets stained with BODIPY in primary SDH-deficient GIST (patient #3) cells. Green, BODIPY (493 nm/503 nm) for lipid droplet staining; blue, Hoechst 33342 (350 nm/461 nm) for nuclear staining; red, MitoTracker Red FM (581 nm/644 nm) for mitochondrial staining. **e** Effects of olverembatinib and other TKIs on lipid uptake in SDH-deficient GIST primary cells from Patient #2. After treatment with DMSO and TKIs at the indicated concentrations for 24 h in basic medium without FBS, the intracellular BODIPY™ FL C16 level in the cells was detected by a multifunction microplate reader at 490 nm/535 nm (*n* = 4). **f** Olverembatinib, but not other TKIs, downregulated CD36 in primary SDH-deficient GIST cells. After the cells were treated with the indicated concentrations of olverembatinib, imatinib, regorafenib, and sunitinib for 24 h, the cell lysates were collected for western blot analysis. **g** Viability response to olverembatinib after SDHB re-expression. Primary SDH-deficient GIST cells from Patients #2 and #3, transfected as in (**f**), were treated with 100 nM olverembatinib 48 h post-transfection. Cell viability was measured to assess olverembatinib sensitivity before and after SDHB restoration. **h** Western blot analysis confirmed SDHB overexpression post-transfection. Reoverexpression of SDHB was carried out by transient transfection of pcDNA3.1-SDHB into primary SDH-deficient GIST cells (patients #2 and #3). Western blots showing the overexpression of SDHB after transfection. NC, cells transfected with the pcDNA3.1 vector plasmid. SDHB-OE, cells transfected with the SDHB-overexpressing pcDNA3.1 plasmid. **i** Lipid uptake response to olverembatinib after SDHB re-expression. Primary SDH-deficient GIST cells from Patients #2 and #3, transfected as in (**f**), were treated with 100 nM olverembatinib 48 h post-transfection. Lipid uptake capacity was evaluated before and after SDHB restoration. ns, not significant; ^*^P < 0.05, ^**^P < 0.01, ^***^P < 0.001

To validate the above hypothesis, we further characterized lipid metabolism in SDH-deficient GIST cells. Initial BODIPY fluorescence staining revealed significantly greater lipid droplet accumulation in SDHB knockout (KO) GIST-T1 cells than in wild-type GIST-T1 cells (negative control, NC) (Fig. 4a), indicating increased lipid uptake after SDHB knockout and that tumor cells with SDHB deficiency may rely more on energy from fatty acid metabolism for growth. Given that cellular free fatty acids are derived primarily from fetal bovine serum (FBS), the cells were cultured in media supplemented with different concentrations of FBS (3% and 10%), and the cells were treated with olverembatinib. Compared with GIST-T1 cells, SDHB-KO GIST-T1 cells exhibited greater dependence on FBS for proliferation and were more sensitive to olverembatinib treatment when cultured in medium supplemented with 3% FBS than when cultured with 10% FBS (Fig. 4b). This observation was confirmed in primary SDH-deficient GIST (patient #1) cells, which showed that cells cultured in medium with 1% FBS are more sensitive to olverembatinib treatment than those cultured with 3% or 10% FBS (Fig. 4c). Furthermore, the effects of olverembatinib on lipid metabolite levels and lipid uptake were investigated and compared with those of other TKIs. As shown in Fig. 4d, much weaker fluorescence staining was observed in SDH-deficient GIST (patient #3) cells treated with 100 nM olverembatinib, indicating the inhibitory effects of olverembatinib on lipid accumulation. Consistent with this observation, olverembatinib suppressed lipid uptake in primary SDH-deficient GIST (patient #2) cells more significantly than other TKIs, as measured by BODIPY labeling (Fig. 4e).

The suppression of lipid metabolism by olverembatinib was further confirmed via lipidomic analysis. Olverembatinib dose-dependently decreased the levels of monoglycerides (MGs), diglycerides (DGs), and triglycerides (TGs), while imatinib had little effect (Supplementary Fig. 5). Next, we observed that CD36 in primary SDH-deficient GIST cells (patients #3 and #4) could be inhibited by olverembatinib but not other TKIs used in the clinic (Fig. 4f).

To further verify the effect of olverembatinib on SDH-associated pathways, we performed transient overexpression (OE) of the SDHB protein in primary SDH-deficient GIST cells (from patients #2 and #3), as described in the Methods. The cells were treated with 100 nM olverembatinib for 48 h after transfection, and cell viability was determined via a CellTiter-Glo^®^ assay kit. As shown in Fig. 4g, overexpression of SDHB rendered both cell lines resistant to olverembatinib treatment, indicating that olverembatinib functions through pathways associated with SDHB deficiency. Notably, CD36 protein levels were downregulated after SDHB was overexpressed in both cell lines (Fig. 4h), which is in line with the finding that CD36 protein levels were higher in tumor samples from patients with SDH-deficient GISTs than in those from patients with SDH-competent GISTs (Fig. 3d). We also observed decreased protein levels of HIF-1α and HIF-2α when SDHB was overexpressed. This finding is consistent with the literature that SDHB deficiency upregulates HIF proteins. The concurrent decrease in HIF-1α, HIF-2α, and CD36 protein levels upon SDHB overexpression indicates that HIF could regulate CD36 expression in SDH-deficient tumors, which has been previously reported.^25–27^ In addition, the suppression of lipid uptake by olverembatinib was also abolished after SDHB overexpression (Fig. 4i).

#### Olverembatinib downregulates other key signaling pathways in SDHB-deficient cells

Western blot analysis using cell lines revealed that, compared to parental PC12 cells, knock-down of SDHB increased hypoxia-inducible factors (HIF-1α, HIF-2α), vascular endothelial growth factor-A (VEGFA), fibroblast growth factor receptor-1 (FGFR1), insulin-like growth factor-1 receptor (IGF-1R), and phosphorylated FGFR1, all of which have been reported to be involved in tumorigenesis because of SDH deficiency (Supplementary Fig. 6). Olverembatinib treatment dose-dependently inhibited these proteins as well as their downstream proteins AKT (protein kinase B) and extracellular signal-regulated kinase 1/2 (ERK1/2). These signaling pathways are involved in tumor cell hypoxia, angiogenesis, proliferation, and survival. Increased cleavage of caspase-3 and poly (ADP-ribose) polymerase 1 was also observed in tumor cell lines treated with olverembatinib, suggesting the induction of apoptosis.

Taking all of the above data together, we propose that decreasing lipid uptake in SDH-deficient tumor cells, whose growth relies more on energy from fatty acids in the tumor microenvironment, compared to non-SDH deficient cells, contributes to a certain extent to the antitumor effects of olverembatinib in this type of cancer. Moreover, because olverembatinib is a multitarget kinase with evident inhibition of several tumorigenic signaling pathways (e.g., HIF, FGFR, VEGFR), these effects could contribute to the superior antitumor efficacy of olverembatinib compared with other TKIs.

## Discussion

Although the advent and use of imatinib have revolutionized care for most patients with advanced GISTs, patients with SDH-deficient disease rarely respond to this agent, and other approved TKIs have not been proved to be efficacious. This study revealed that olverembatinib, a novel multikinase inhibitor, has clinical activity in patients with advanced SDH-deficient GIST and has a manageable safety profile and durable response, with a median PFS of 25.7 months. To our knowledge, this is the largest prospective clinical trial conducted to date that focused on the treatment of patients with TKI-resistant SDH-deficient GISTs.

Most patients with SDH-deficient GISTs included in this study were female, younger, and had a primary gastric site of tumor origin. These clinicopathological associations are consistent with the literature concerning SDH-deficient GISTs.^6,12^ Loss of SDHB protein expression occurs from biallelic inactivation of any of the *SDH* genes by mutation or hypermethylation of the *SDHC* gene promoter, which leads to SDH deficiency.^4,7^ Approximately half of the patients have SDH subunit gene mutations, often germline and most commonly A (30%), B, C, or D (together 20%).^12,28^ Most other SDH-deficient GISTs (~50% of patients) lack *SDH* mutations but exhibit hypermethylation at the *SDHC* promoter site.^4,7^ In our study, germline *SDHB* was the most common *SDH* mutation (in 12/26 patients), with only 1 case each of germline *SDHA* and *SDHC* mutations. It is unclear why the frequency of germline *SDH* mutations in our study differed from previously reported values, but one potential reason is that all patients in our study were of Asian descent.

The role of TKIs in the treatment of patients with SDH-deficient GISTs remains controversial. Approved TKIs have shown limited efficacy, and studies of these agents in metastatic SDH-deficient GISTs have reported a median progression-free survival (PFS) of 6–10 months.^13^ The development of new drugs is constrained by the dual challenges of insufficient sample size and suboptimal efficacy. Linsitinib, an oral TKI IGF-1R inhibitor, showed no objective responses in 20 patients with WT GISTs (including 17 SDH-deficient GISTs) and 9-month estimated PFS was 52% in a phase 2 trial.^29^ Guadecitabine, a dinucleotide containing the DNA methyltransferase inhibitor decitabine, showed no partial or complete responses in 9 SDH-deficient patients (including 7 SDH-deficient GISTs) in a phase 2 trial.^30^

Considering that more than 60% of the patients in our study were heavily pretreated with more than two TKIs, it is noteworthy that patients receiving olverembatinib experienced a clinical benefit rate (CBR) of more than 80% and a median PFS of 25.7 months, compared with 2.9 months in non-SDH-deficient GIST patients. These levels of disease control and response durability appear to exceed all the literature reports of prospective clinical trials. In addition, olverembatinib was well tolerated in this group of patients, with a frequency and severity of adverse events that were lower than those in patients with CML.^20^ Common drug-related toxicities were chiefly grade 1 or 2 and generally manageable with standard supportive medical treatments. Notably, no patients discontinued treatment because of AEs.

With respect to the MOAs of olverembatinib in this type of rare cancer, first, biomarker analysis using tumor samples from patients in the current clinical trial offered clues that better clinical efficacy of olverembatinib is associated with upregulated lipid metabolism in SDH-deficient GIST patients. Second, RNA sequencing and lipidomic analyses using banked tumor samples from GIST patients collected at the collaborating hospital revealed significant glycerol lipid enrichment in the tissues of SDH-deficient patients compared with non-SDH-deficient patients, with the overexpression of genes related to lipid uptake, including *CD36, FABPs*, and *FATPs*. Among these genes, *CD36* is a major player in metabolic tissues.^31,32^ CD36 accounts for 50% of FA uptake in adipose tissues and muscle in mice. Humans with CD36 deficiency show significantly decreased FA uptake in heart, muscle, and adipose tissues.^33,34^ Third, the immunofluorescence data revealed that SDH-deficient GIST-TI-SDHB-KO cells have increased lipid droplet storage, confirming the increased lipid uptake observed via RNA sequencing and lipidomic analyses. Further data revealed lipid droplet accumulation and lipid uptake, as well as that CD36 protein levels could be suppressed by olverembatinib but not other TKIs. Finally, the effects of olverembatinib on SDH-associated pathways were further validated via rescue experiments. The overexpression of SDHB in SDH-deficient tumor cells rendered the cells more resistant to olverembatinib treatment and abolished the ability of olverembatinib to suppress lipid uptake. Moreover, downregulation of CD36 was observed when SDHB was overexpressed, which is in agreement with the findings that CD36 protein levels in tumor samples were higher in SDH-deficient GIST patients than in SDH-competent GIST patients and confirmed that the upregulation of CD36 and lipid uptake was due to SDHB deficiency. The concurrent decrease in HIF-1α/2α upon SDHB overexpression indicated that CD36 could be regulated by HIF proteins, which was previously reported.^25–27^

On the basis of the above findings, we propose that SDH-deficient GIST tumors may rely on exogenous lipids to provide energy for cellular metabolism, which could be interfered with by olverembatinib and contribute to the antitumor effects of the drug. The regulation of lipid metabolism was observed with olverembatinib but not with other TKIs clinically used for SDH-deficient GIST treatment, which could explain the superior clinical efficacy of olverembatinib. To our knowledge, this is the first study to establish a relationship between SDH deficiency and lipid metabolism. Although many questions remain to be answered, such as how olverembatinib regulates *CD36* or other genes involved in lipid metabolism, the results from our study provide a new direction for revealing MOAs of TKIs in SDH-deficient GIST.

In addition to its effect on lipid metabolism, we also showed that olverembatinib downregulated several reported tumorigenic pathways in SDH-deficient GISTs, the most reported of which involve succinate-induced signaling pathways (HIF-1α/2α, IGF-1R, and VEGF) and the FGFR pathway, which is highly expressed in SDH-deficient GISTs.^10^ The results from in vitro biochemical assays indicate that olverembatinib inhibits the kinase activities of VEGFRs (1, 2, and 3) and FGFRs (1, 2, 3 and 4) at IC_50_ values lower than 0.6 nM. To further investigate the inhibitory effects of olverembatinib, we performed antiproliferation assays in primary SDH-deficient GIST cells and SDHB-knockout PC12#5F7 cells treated with olverembatinib and compared the results with those of agents that target various pathways: HIF-α (belzutifan, BAY 87-2243, PX-478); IGF-1R (linsitinib); VEGFR (sunitinib, regorafenib); and FGFR (rogaratinib, pemigatinib). The results (Supplementary Fig. 4 and data not shown) demonstrated that olverembatinib is more potent than all other TKIs at inhibiting cell growth, which could be explained by the multitargeting properties of olverembatinib. Angiogenesis is an important feature of cancer cells, and although available in vitro data have demonstrated that olverembatinib potently inhibits VEGFRs, a lack of suitable SDH-deficient GIST animal models is a significant hurdle preventing potential evaluation of the antiangiogenic effects of the compound. Taken together, these findings suggest that the tumor-killing effect of olverembatinib in this type of cancer could be a combinatorial outcome of its ability to modulate deregulated lipid metabolism and tumorigenic pathways. Nevertheless, the specific mechanism by which olverembatinib regulates lipid metabolism, or the interplay between lipid metabolism and the abovementioned tumorigenic signaling pathways, awaits further comprehensive investigation.

Our findings should be considered in light of potential study limitations. First, even though this is the largest global prospective clinical trial evaluating a TKI for SDH-deficient GIST, it was an open-label, single-arm, phase 1b study, which is consistent with the rarity of SDH-deficient- GIST. Second, at the time of the analysis, the secondary endpoint of PFS was not sufficiently mature to further interpret outcomes. Third, no testing was performed to detect SDHC methylation in patients who did not have SDH gene mutations. However, there was no statistically significant difference in the efficacy data between the groups with and without SDH mutations.

**Fig. 5:**
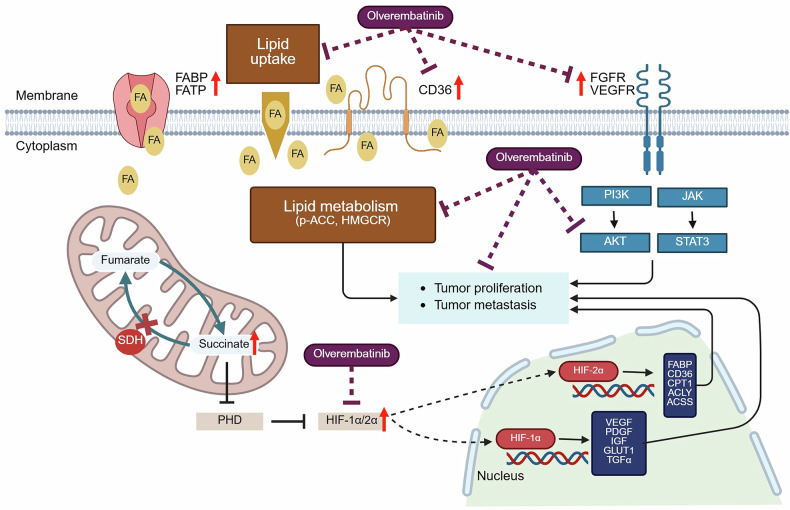
Proposed mechanisms of action of olverembatinib in SDH-deficient GIST. Red arrows represent upregulation in the SDH-deficient GIST cells. The purple dotted lines with a bar represent inhibition by olverembatinib. Increased lipid uptake, lipid synthesis, and FABP, FATP and CD36 protein expression are found in SDH-deficient GIST tumor cells, and olverembatinib treatment dose-dependently suppressed lipid uptake, CD36, and proteins involved in the regulation of lipid metabolism. The accumulation of cellular succinate due to SDH deficiency leads to the inhibition of prolyl hydroxylases (PHDs), which in turn results in hypoxia-inducible factor (HIF) stabilization and HIF activation. Activated HIF1α/2α induces the expression of genes responsible for FA uptake and triacylglycerol (TAG) synthesis. Consequently, lipid enrichment in SDH-deficient GIST tumor cells may provide a major energy source for cell proliferation and metastasis. Moreover, stabilized HIF1α upregulates the expression of genes, such as Glut1, IGF, TGFα, and VEGF, that are involved in angiogenesis; glucose metabolism; and cell proliferation, survival, invasion, and metastasis. The results reported in this study demonstrated that olverembatinib blocks the energy source of SDH-deficient tumor cells by suppressing lipid uptake as well as lipid metabolism, which has not been previously reported with other TKIs used for the treatment of SDH-deficient cancers. In addition, olverembatinib inhibits other signaling pathways, including the VEGFR, IGF1R, FGFR, and SRC pathways, which have been implicated in the tumorigenesis of SDH-deficient neoplasms. Taken together, these findings indicate that olverembatinib exerts antitumor effects by regulating lipid uptake, lipid metabolism, and multiple tumorigenic pathways involved in proliferation, angiogenesis, apoptosis, and survival. Created in BioRender. Yang, C. (2025) https://BioRender.com/to2gqhf (agreement number: VO28QGKQUY)

In conclusion, in this trial of prospectively enrolled patients with SDH-deficient GISTs, olverembatinib was well tolerated, the CBR was more than 80%, and the estimated median PFS was significantly prolonged, indicating a potential benefit of this treatment and providing a benchmark for future studies in this rare subtype of GIST. On the basis of our findings, we propose that MOAs of olverembatinib in SDH-deficient tumor cells involve the regulation of lipid metabolism (previously unreported), including the suppression of FA uptake; the suppression of HIF and CD36; and the modulation of multiple signaling pathways involved in hypoxia, angiogenesis, apoptosis, proliferation, and survival (Fig. 5). Further studies to validate these preliminary findings and elucidate the MOA of olverembatinib are ongoing.

## Materials and methods

### Ethics statement

This study was performed in accordance with the ethical principles of the Declaration of Helsinki and was consistent with the International Conference on Harmonisation Good Clinical Practice. The institutional review board or independent ethics committee at each site approved the study. This included the following sites and reference numbers: Sun Yat-sen University Cancer Center (A2018-020); Fudan University Shanghai Cancer Center (1904199-2); Affiliated Cancer Hospital of Zhengzhou University and Henan Cancer Hospital (2019290); Union Hospital, Tongji Medical College, Huazhong University of Science and Technology (No.14, 2019); Guangdong Provincial People’s Hospital (2019-14); and The First Medical Center of the People’s Liberation Army (C2018-060). All patients provided written informed consent before participating in the study.

### Study design and conduct

This multicenter, open-label, phase 1 study (ClinicalTrials.gov identifier: NCT03594422) evaluating the safety and antitumor efficacy of olverembatinib monotherapy was conducted in two parts. Part 1 was a dose-escalation study to determine the recommended phase 2 dose (RP2D) in solid tumors. Oral olverembatinib was administered every other day (QOD) on the basis of the ~20-h half-life of the compound.^20^ The starting dose was 20 mg, followed by sequential doses of 30, 40, or 50 mg in continuous 28-day cycles. The initial design was a 3 + 3 dose escalation from 20 mg, but the protocol was amended to randomly enroll and assign patients to starting doses of 30, 40, and 50 mg on the basis of safety data acquired in the CML trial.^20^ Patients were randomly assigned at a 1:1:1 ratio to receive one of three olverembatinib monotherapy doses: 30, 40, or 50 mg. An interactive web response system was used for randomization. Part 2 was a nonrandomized study focusing on patients with SDH-deficient GISTs. The RP2D of olverembatinib was determined to be 40 mg QOD on the basis of safety data from a previous phase 1 study^20^ and randomization in Part 1. On the basis of preliminary data from adult patients, adolescent patients with advanced wild-type GISTs were also included in Part 2. The dosage for adolescents was adjusted according to their body weights. If patients weighed at least 40 kg, they received 40 mg of olverembatinib (QOD); if they weighed between 30 and 40 kg, they received 30 mg QOD; and if they weighed between 20 and 30 kg, they received 20 mg QOD.

### Patients

Eligible patients included those aged 12 years or older with histologically confirmed advanced GISTs or other solid tumors. Patients with GISTs had primary resistance to imatinib (i.e., tumor progression within 6 months after first-line use of imatinib, SDH-deficient tumors verified by immunohistochemistry, or tumors with NF1 mutation) or had failed treatment with either imatinib or imatinib plus at least one other TKI. Twenty-four of the 26 patients with SDH-deficient GISTs were primarily resistant to imatinib. The definition of SDH-deficient GIST was based on negative immunohistochemical staining for SDHB (Supplementary Fig. 7). In addition, study enrollment required an Eastern Cooperative Oncology Group performance status ≤2 and adequate renal, hepatic, cardiac, and bone marrow function. A full list of inclusion and exclusion criteria is included in the clinical trial protocol.

### Study assessments

AEs were evaluated at each follow-up visit: on Days 1, 8, 15, and 22 during Cycles 1 and 2; and on Days 1 and 15 in Cycle 3 and all subsequent cycles, up to 30 days after the final dose. All AEs were graded according to the National Cancer Institute Common Terminology Criteria for Adverse Events v4.03. Clinical laboratory investigations for routine blood examinations and serum chemistry parameters were performed during every cycle (on Day 1), during Cycles 1 and 2 (on Days 8, 15, and 22), and 30 days after the last dose of treatment. Urinalysis, coagulation, cardiac troponin I/T, lipase, amylase, and electrocardiographic evaluations were conducted at baseline; during Cycles 1 and 2 (on Days 1 and 15); during Cycles 3, 5, 7, and 9; every three cycles after Cycle 9 (on Day 1); and 14 days after the last dose of treatment. All patients underwent tumor imaging for response assessment via computed tomography or magnetic resonance imaging at screening and every two cycles (8 weeks) until progression or discontinuation. Target and nontarget lesions were assessed per the Response Evaluation Criteria in Solid Tumors version 1.1 (RECIST v1.1).

Serial blood samples were collected to measure olverembatinib plasma concentrations after dosing on Days 1 and 27 of Cycle 1 and before dosing on Days 15, 17, and 19. Noncompartmental pharmacokinetic analysis was conducted via Phoenix WinNonlin software (version 6.4). Pharmacokinetic parameters, including the area under the plasma concentration–time curve (AUC), maximum observed plasma concentration (*C*_max_), and apparent terminal elimination half-life (*t*_1/2_), were derived for olverembatinib.

### Study outcomes

The primary endpoints were the determination of the RP2D and safety profile of olverembatinib. The secondary endpoints included efficacy assessments (overall response rate [ORR], clinical benefit rate [CBR], PFS per RECIST v1.1 and overall survival [OS]) and pharmacokinetic properties. The CBR was defined as the proportion of patients with a confirmed complete response (CR) or partial response (PR) of any duration or stable disease (SD) for at least 6 months from the start of treatment. The ORR was defined as the proportion of patients with a confirmed CR or PR of any duration. PFS was defined as the time from the start of treatment to the date of first documented disease progression or death due to any cause, whichever occurred first. OS was defined as the time from the start of treatment until the date of death.

### RNA sequencing

After informed consent was obtained, tumor tissue samples were collected from patients prior to olverembatinib treatment and processed via formalin fixation and paraffin embedding (FFPE). Total RNA was extracted from blocks of FFPE tumor samples from seven patients (three PRs and four SDs; Supplementary Table 6) via the RNeasy FFPE Kit (Catalog # 217504; Qiagen, Hilden, Germany). Ribosomal RNA and genomic DNA were removed, and the remaining RNA was purified via Agencourt^®^ RNAClean^TM^ XP beads (Catalog # A63987; Beckman Coulter, Beverly, MA, USA). Sequencing libraries were constructed via the KAPA Stranded RNA-Seq Library Preparation Kit (Catalog # KK8482 and KK8401, KAPA Biosystems, Wilmington, MA, USA) and then sequenced on the DNBSEQ-T7 NGS platform (MGI Tech Co., Ltd, Shenzhen, China). Transcription sequencing of cancer-related genes was performed via the Sarcorna^TM^ panel (Geneseeq Technology Inc., Nanjing, China). Transcriptome mapping was performed via HISAT2 (version 2.2.1), and gene expression was analyzed via StringTie (version 2.2.1). Differentially expressed genes were analyzed via edgeR (version 3.40.2) for up- and downregulated gene screening (|fold change| > 2, *P* < 0.05). Gene Ontology (GO) analysis, Kyoto Encyclopedia of Genes and Genomes (KEGG) enrichment analysis, and gene set enrichment analysis (GSEA) were performed via clusterProfiler (version 4.6.2).

### Statistical analysis

The data cutoff date for this analysis was December 27, 2023. The safety population included all patients who received at least one dose of olverembatinib. Antitumor activity was evaluated in patients from the GIST population (including those with SDH-deficient and non-SDH-deficient disease) who had undergone at least one postbaseline disease assessment. The Kaplan‒Meier method was used to estimate PFS, and the corresponding 95% CIs were provided. All the statistical analyses were performed via SAS (v9.3 or higher; SAS Institute, Cary, NC).

### Information on the SDH-deficient cell lines

A rat pheochromocytoma cell line (PC12) was obtained from Cobioer Biosciences Co., Ltd (Nanjing, China). The PC12 SDHB knockdown (KD) cell line (PC12#5F7) was obtained from GENEWIZ, Inc. (Azenta Life Sciences; Suzhou, China) via a sgRNA-mediated clustered regularly interspersed short palindromic repeat (CRISPR)/Cas9 system. Jurkat, Clone E6-1, and OS-RC-2 cell lines were obtained from the Cell Resource Center, Shanghai Institutes for Biological Sciences, Chinese Academy of Sciences. The GIST-T1 cell line was obtained as a gift from Dr. Jonathan Fletcher’s laboratory at Brigham and Women’s Hospital, Harvard Medical School, USA. RKO was kindly gifted by Prof. Shaomeng Wang’s laboratory at the University of Michigan, USA. All the cell lines were cultured in the recommended medium at 37 °C in an atmosphere of 5% CO_2_ (Supplementary Table 10).

### Analyses of human GlST tissue samples

Frozen GIST tissues collected from 15 patients were used for RNA sequencing and LC‒MS; primary cells isolated from fresh tumor tissues of 4 patients with SDH-deficient GISTs were used for ex vivo analyses. All samples were collected at Sun Yat-sen University Cancer Center, with informed consent obtained from each subject. The study, which involved human subjects, was approved by the Medical Ethics Committee of Sun Yat-sen University Cancer Center.

Tissues from GIST patient samples were digested with 1 mg/mL collagenase IV at 37 °C for 30 min after being cut into pieces, followed by filtration of the suspension into single cells. Finally, the cell suspensions were collected and resuspended in GIST primary cell culture medium [(DMEM)/F12 medium containing 10% FBS, 1% GlutaMAX™ (35050061, Thermo Fisher), 1% Anti-Anti (15240062, Gibco), 50 ng/mL SCF (300–07–10, PPL), 125 ng/mL EGF (PK0014–505, PPL), 20 ng/mL bFGF (HY-P7004, MCE), 25 ng/mL hydrocortisone (HY-N0583, MCE), and 5 µg/mL insulin (HY-P0035, MCE)]. The cells were seeded in 96-well plates (5000 cells per well) and cocultured with different concentrations of olverembatinib or other TKIs for 72 h. Finally, cell proliferation was detected via a CellTiter 96^®^ Aqueous Non-Radioactive Cell Proliferation Assay (G7570, Promega, Madison, WI, USA).

### Reagents

Olverembatinib was manufactured by Jiangsu Ascentage Pharma Group Corp. Ltd. (Taizhou, China). Imatinib, avapritinib, ponatinib, dasatinib, sunitinib, regorafenib, sorafenib, pazopanib, rogaratinib, and pemigatinib were purchased from Jiangsu Aikon Biopharmaceutical R&D Co., Ltd. Ripretinib was purchased from Selleck Chem (Houston, TX, USA). Infigratinib was purchased from MCE (MedChemExpress).

### Cell proliferation assay

A dose range of the indicated compounds was prepared via serial dilutions and added to 96-well plates, followed by the seeding of cells at optimum densities per well. The cells were incubated with drugs for 72 h, and cell growth was quantified via a Cell Counting Kit-8 (Shanghai Liji Medical Technology Co., Ltd) or CellTiter-Glo^®^ Luminescent Cell Viability Assay Kit (G7573, Promega). Graphs were generated via Prism 9 (GraphPad Software, La Jolla, CA, USA).

### Western blot analysis

Harvested cells treated with dimethyl sulfoxide (DMSO) or the indicated concentrations of compounds were dissolved in 1× SDS sample lysis buffer. Equal amounts of protein were separated by 4–20% SDS‒PAGE and transferred to nitrocellulose membranes for immunoblotting. Finally, the target proteins were visualized via an enhanced chemiluminescence (ECL) western blotting detection kit (Yeasen). The primary antibodies used included FGF3 (PA5-102906) and FGF4 (PA5-115228) from Thermo Fisher Scientific (Waltham, MA, USA); HIF-2α (PA116510) from Invitrogen; SDHB (AB14714), HIF-1α (ab16066), VEGFA (ab214424), and IGF-1R (ab182408) and CD36 (ab133625) from Abcam; and phosphorylated (p-) FGFR1 (52928), FGFR1 (9740), p-SRC (6943), SRC (2109), p-VEGFR2 (3770), VEGFR2 (9698), p-AKT (4060), AKT (4685), p-ERK (4370), ERK (4695), p-IGF-1Rβ (3021), C-MYC (13987), p-STAT3 (9145), STAT3 (9139), PARP-1 (9532), caspase-3 (9665), cleaved caspase-3 (9664), and β-actin (3700) from Cell Signaling Technology (CST).

### Lipid metabolite assay using LC‒MS

SDH-deficient and non-SDH-deficient patient tissues were collected, and lipids were extracted and analyzed via liquid chromatography‒mass spectrometry (LC‒MS) according to a previously described method.^35^

### Lipid droplet staining

#### Lipid droplet visualization and quantification

Cellular lipid droplets were fluorescently labeled with BODIPY™ 493/503 (Thermo Fisher Scientific, USA; 2 µM). Following fixation, the cells were incubated with BODIPY working solution for 30 min at 37 °C under light-protected conditions. Subsequent counterstaining was performed with MitoTracker® Red FM (Thermo Fisher Scientific, USA; 200 nM) for mitochondrial visualization and Hoechst 33342 (Bio-Rad, USA; 1 µg/mL) for nuclear staining. High-resolution confocal imaging was conducted using a Zeiss LSM 980 system. For quantitative analysis, six randomly selected cells per experimental group were subjected to lipid droplet enumeration via ImageJ software. The fluorescence intensity thresholds were standardized across all the samples to ensure consistent particle detection.

#### Lipid uptake assay

To evaluate fatty acid uptake, primary SDH-deficient GIST cells were treated with DMSO (vehicle control), imatinib, olverembatinib, sunitinib, or regorafenib at different concentrations in serum-free DMEM/F12 basal medium for 24 h. Following treatment, the cells were incubated with the fluorescent fatty acid analog BODIPY™ FL C16 (Thermo Fisher Scientific, USA; 0.2 μM) at 37 °C for 4 h. Cellular internalization of the fluorescent probe was quantified by measuring the intracellular fluorescence intensity via a multimode microplate reader (BioTek Synergy H1, Molecular Devices) with excitation/emission wavelengths set at 490 nm and 525 nm, respectively.

#### SDHB overexpression

SDHB cDNA fragments were subcloned and inserted into the pcDNA3.1 vector plasmid (AZENTA, China), and the pcDNA3.1 vector served as the mock vehicle (NC). Before transfection, SDH-deficient tumor cells were cultured in 6-well plates for 24 h and then transiently transfected with the corresponding vector via Lipofectamine 3000 Transfection Reagent (Invitrogen, Carlsbad, CA, USA) according to the manufacturer’s protocol. After 48 h, the cells were treated with olverembatinib at the indicated concentrations, and cell viability or lipid uptake assays were performed as described above. Concurrently, some cells were harvested for western blotting to determine the protein levels of SDHB and CD36.

## Supplementary information

Supplementary Figures and Tables

Supplementary Figure 1

Supplementary Figure 2

Supplementary Figure 3

Supplementary Figure 4

Supplementary Figure 5

Supplementary Figure 6

Supplementary Figure 7

HQP1351 SJ0003 Protocol

Original films of western blots

## Data Availability

All sequencing data generated in this study have been deposited on GSA human (https://ngdc.cncb.ac.cn/gsa-human) under the project PRJCA026539. All the data generated or analyzed during this study are available from the corresponding authors.
