# Supplementary material for: Olverembatinib, a multikinase inhibitor that modulates lipid metabolism, in advanced succinate dehydrogenase-deficient gastrointestinal stromal tumors: a phase 1b study and translational research
Source: Signal Transduct Target Ther. 2025 Nov 4;10:361. doi: 10.1038/s41392-025-02456-9 (PMC12583704; doi:10.1038/s41392-025-02456-9)
Supplement: Supplementary file 1 — Supplementary Figures and Tables [file 41392_2025_2456_MOESM1_ESM.docx]

Supplementary Materials for

Olverembatinib, a multikinase inhibitor that modulates lipid metabolism, in advanced succinate dehydrogenase-deficient gastrointestinal stromal tumors:
A phase 1b study and translational research

Hai-Bo Qiu, Zhiyan Liang, Jing Yang, Ye Zhou, Zhi-Wei Zhou, Xiang-Bin Wan, Ning Li, Kai-Xiong Tao, Yong Li, Xin Wu, Chen Yang, Zi Chen, Hengbang Wang, Lichuang Men, Yan Xiong, Lihui Liu, Dajun Yang, Yifan Zhai, and Rui-Hua Xu

Correspondence to: [dyang@ascentage.com](mailto:dyang@ascentage.com), [yzhai@ascentage.com](mailto:yzhai@ascentage.com), [xurh@sysucc.org.cn](mailto:xurh@sysucc.org.cn)

**This PDF file includes:**

Figures. S1 to S7

Tables. S1 to S10


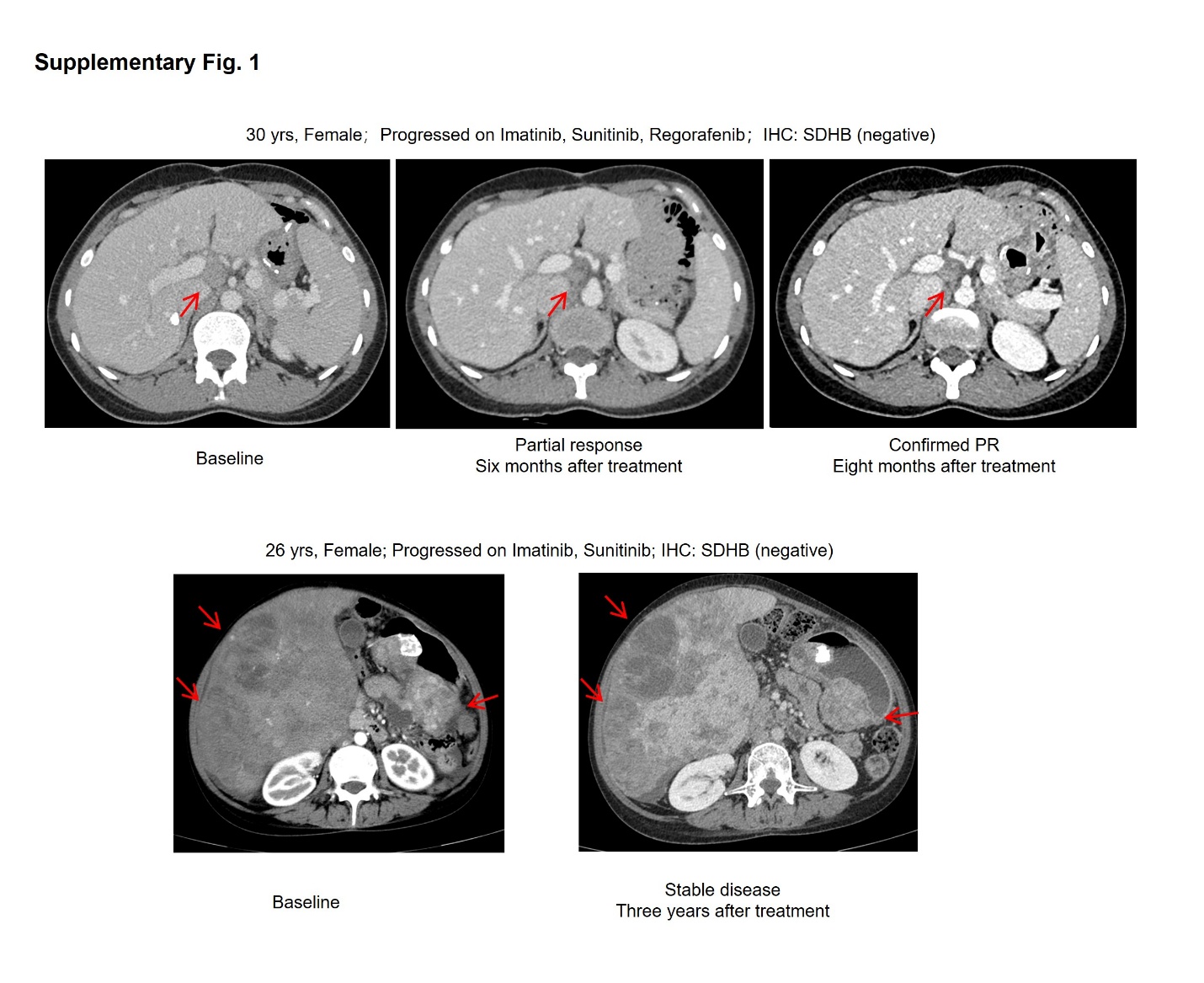


**Figure. S1. Representative CT images of two patients treated with olverembatinib.**

One with confirmed PR, another with durable SD. Red arrows indicate targeted lesions.


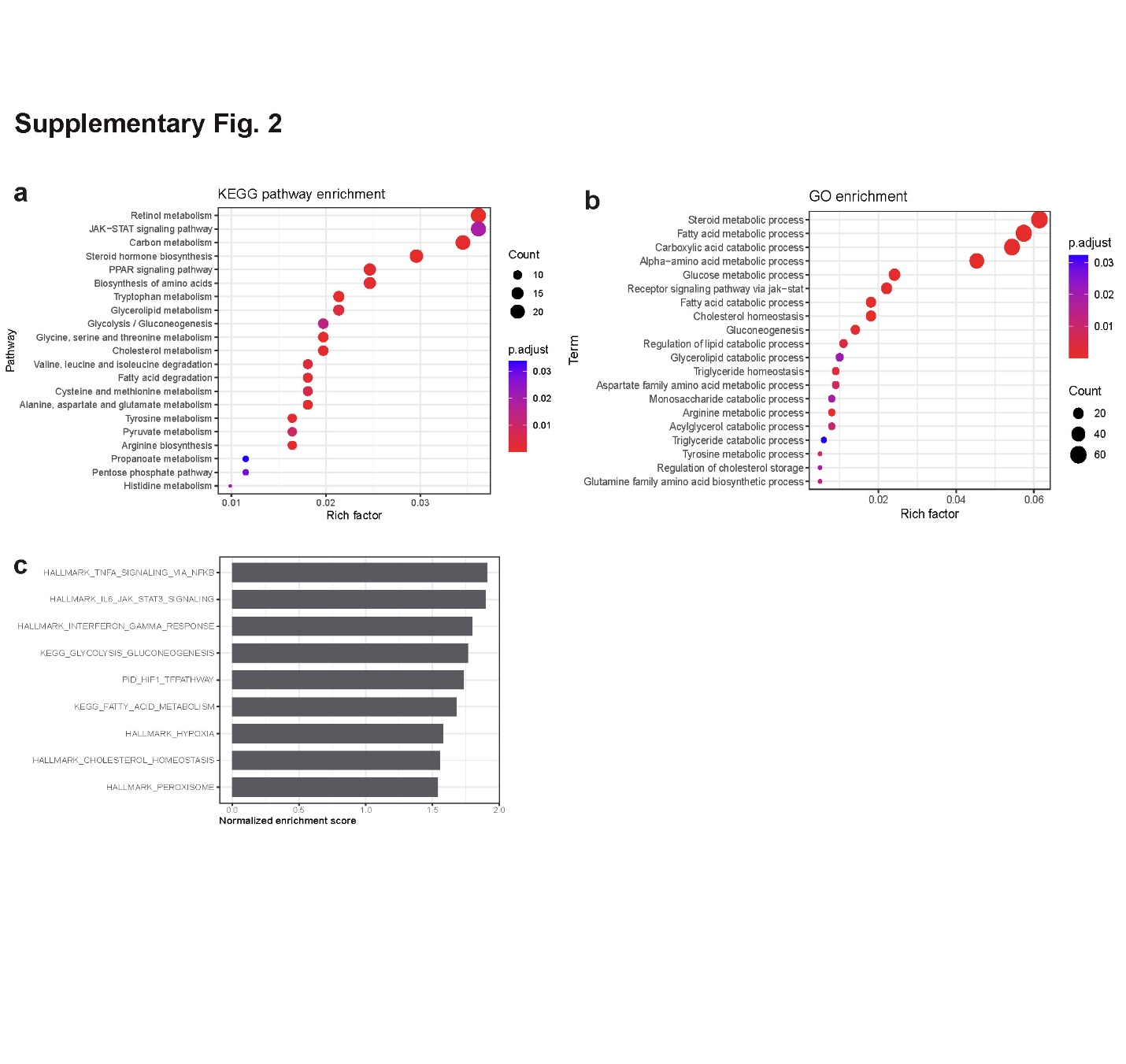


**Figure. S2. Differential gene expression analysis and enrichment results of transcriptome sequencing samples in succinate dehydrogenase–deficient gastrointestinal stromal tumour patients with partial response compared to patients with stable disease.**

**a** Kyoto encyclopaedia of genes and genomes (KEGG) enrichment analysis of upregulated genes in patients with partial response. The vertical coordinate lists the pathway; the horizontal coordinate represents the enrichment factor (the number of up-regulated genes divided by the total number of genes in the pathway); the size of the bubble indicates the number of up-regulated genes in the pathway; and the redder the color, the smaller is the q-value. **b** Gene Ontology (GO) enrichment analysis of up-regulated genes in patients with partial response. The description of the coordinates, size of the bubble, and color are the same as shown in KEGG enrichment plot. **c** Gene Set Enrichment Analysis (GSEA) enrichment bar plots of signaling pathways.

**
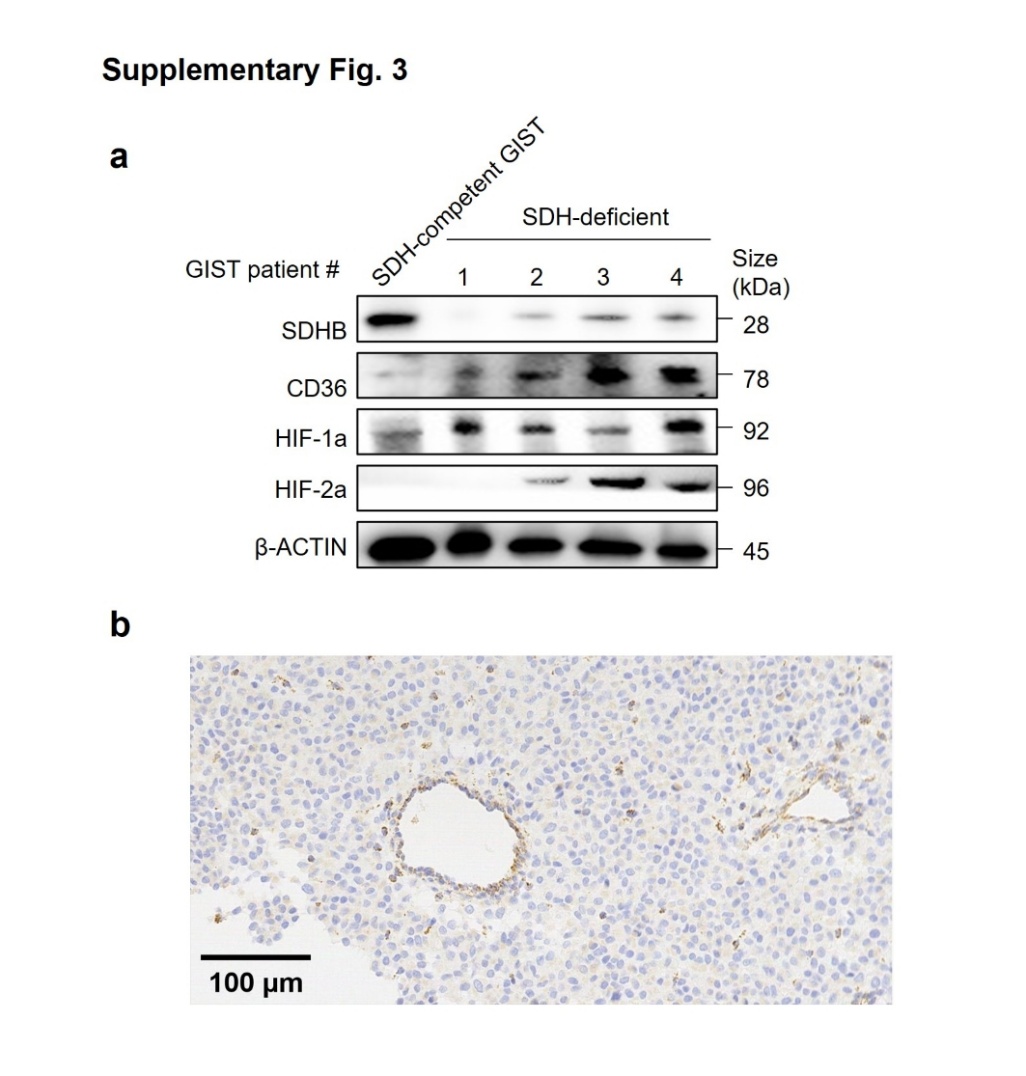
**

**Figure. S3. The expression of SDHB, CD36, and HIF1/2 in primary SDH-deficient GIST cells from patients #1-4.**

**a** SDHB protein expression was confirmed in primary samples from patients with SDH-deficient GIST (patient #1-4). Expression levels of CD36, HIF-1 alpha, and HIF-2 alpha were also evaluated. β-actin was used as a loading control. **b** Immunohistochemistry (IHC) staining of SDHB in SDH-deficient GIST patient (patient #1). The brown staining is the internal positive control of nonneoplastic cells (e.g., vascular, smooth muscle, and epithelial elements).


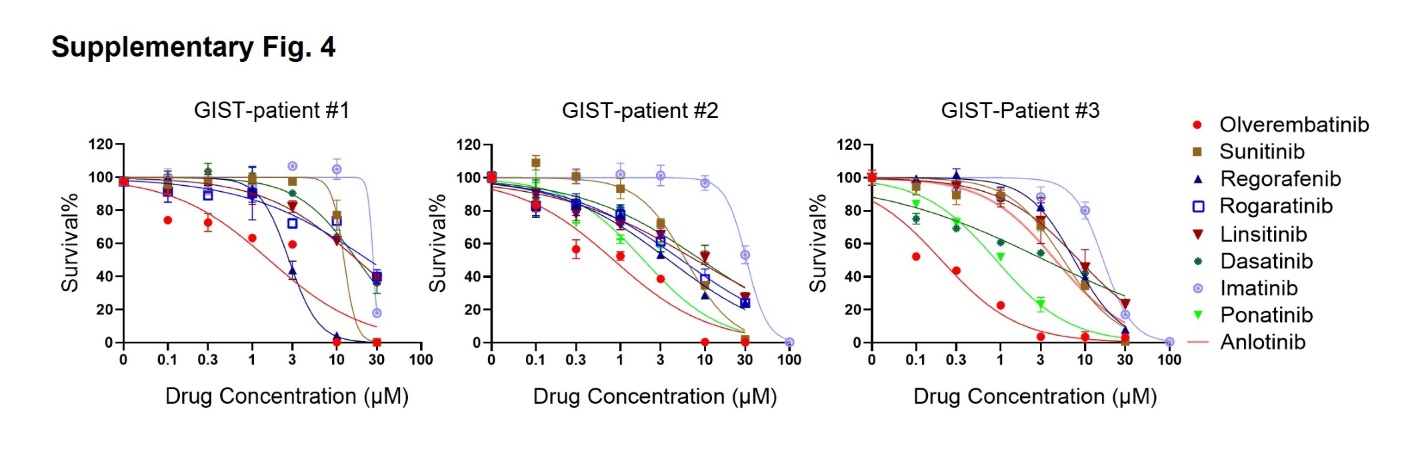


**Figure. S4. Potency of olverembatinib in the tumor cells freshly isolated from tumor specimens of the patients with SDH-deficient GIST.**

Antiproliferation activity of olverembatinib in SDH-deficient primary GIST cells, compared with other TKIs. Cells were extracted from SDH-deficient patient tumor tissues, seeded into 96-well plates, and incubated for 24 hours in GIST culture medium. Cells were exposed to olverembatinib and other TKIs for 72 hours, and CellTiter 96^®^ Aqueous Non-Radioactive Cell Proliferation Assays were used to assess cell viability.

**
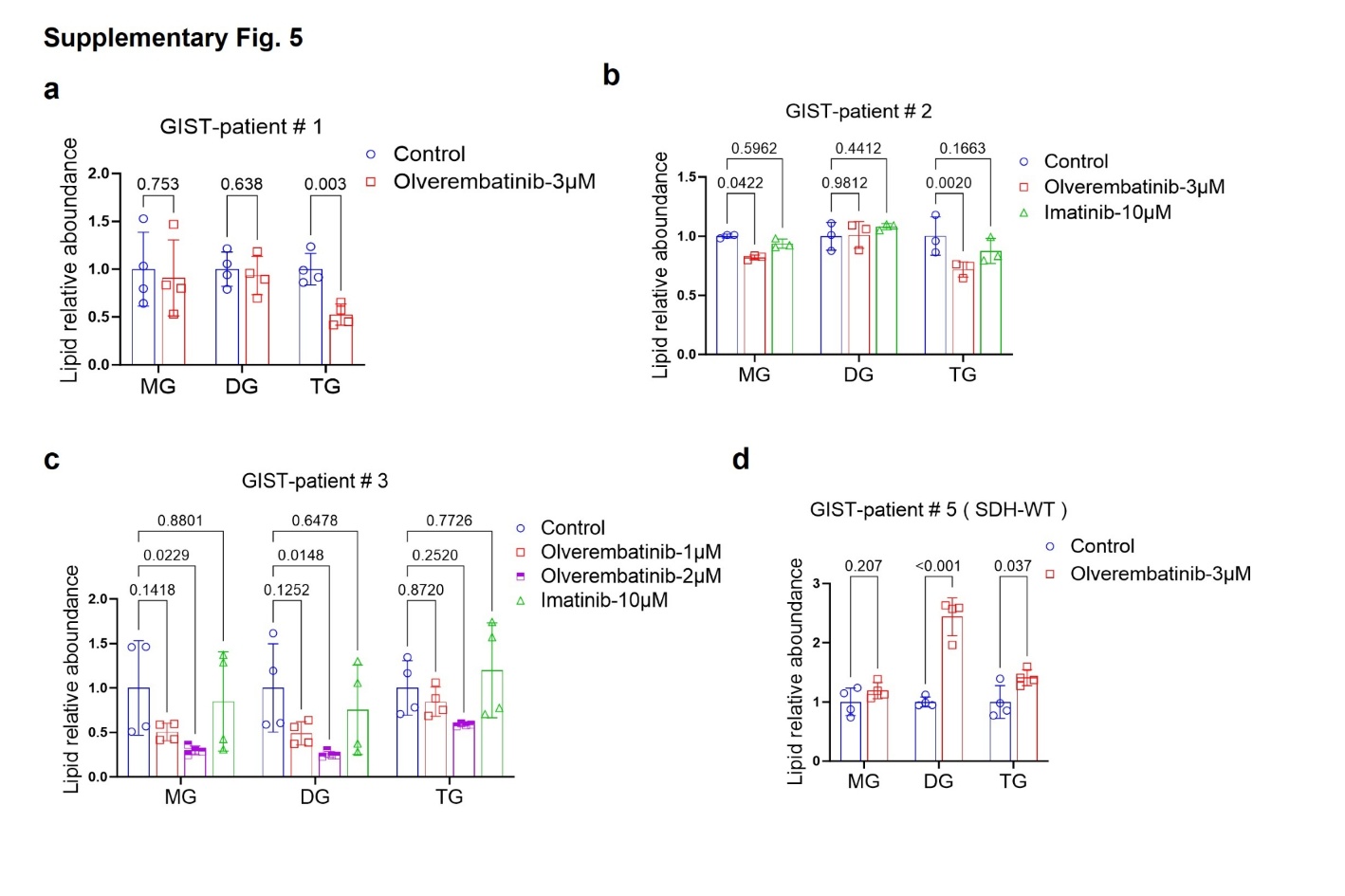
**

**Figure. S5. Olverembatinib suppressed lipids in SDH-deficient GIST cells but not in SDH WT GIST cells.**

Lipidomic analysis of the levels of glycerol esters (GL), including monoglycerides (MG), diglycerides (DG), and triglycerides (TG), in primary cells from SDH-deficient GIST patients (#1, 2, and 3) and SDH WT GIST patient (#5) after treatment with indicated concentrations of olverembatinib or imatinib for 24 hours.


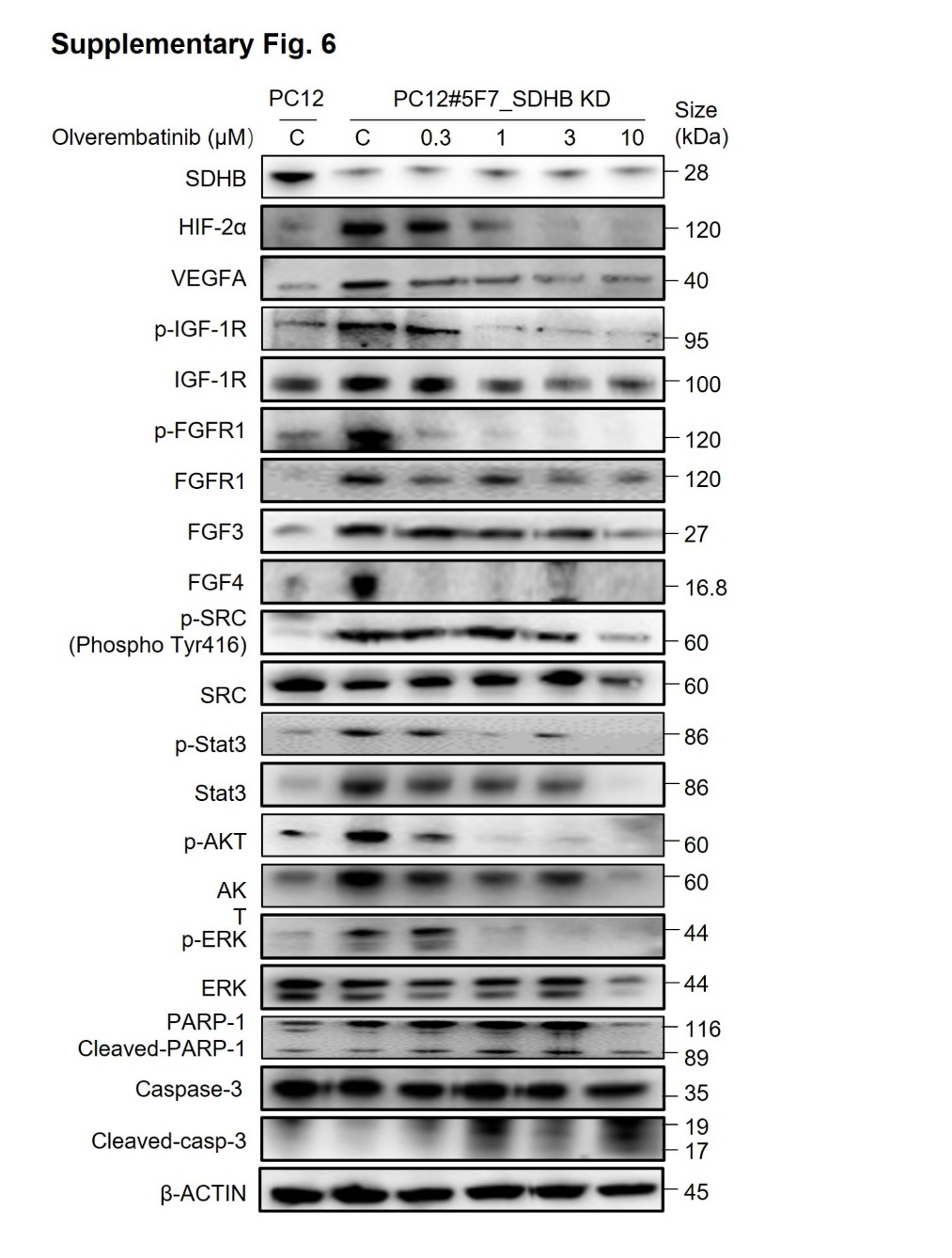


**Figure. S6. Molecular pathways regulated by olverembatinib in SDH knock-down (KD) tumor cells.**

Olverembatinib regulates multiple oncogenic signaling proteins in SDHB KD PC12#5F7 cells. After the SDHB KD PC12#5F7 cells were treated with olverembatinib at the indicated concentrations for 24 hours, cell lysates were collected for western blot analysis. p, phosphorylation; C, control.

**
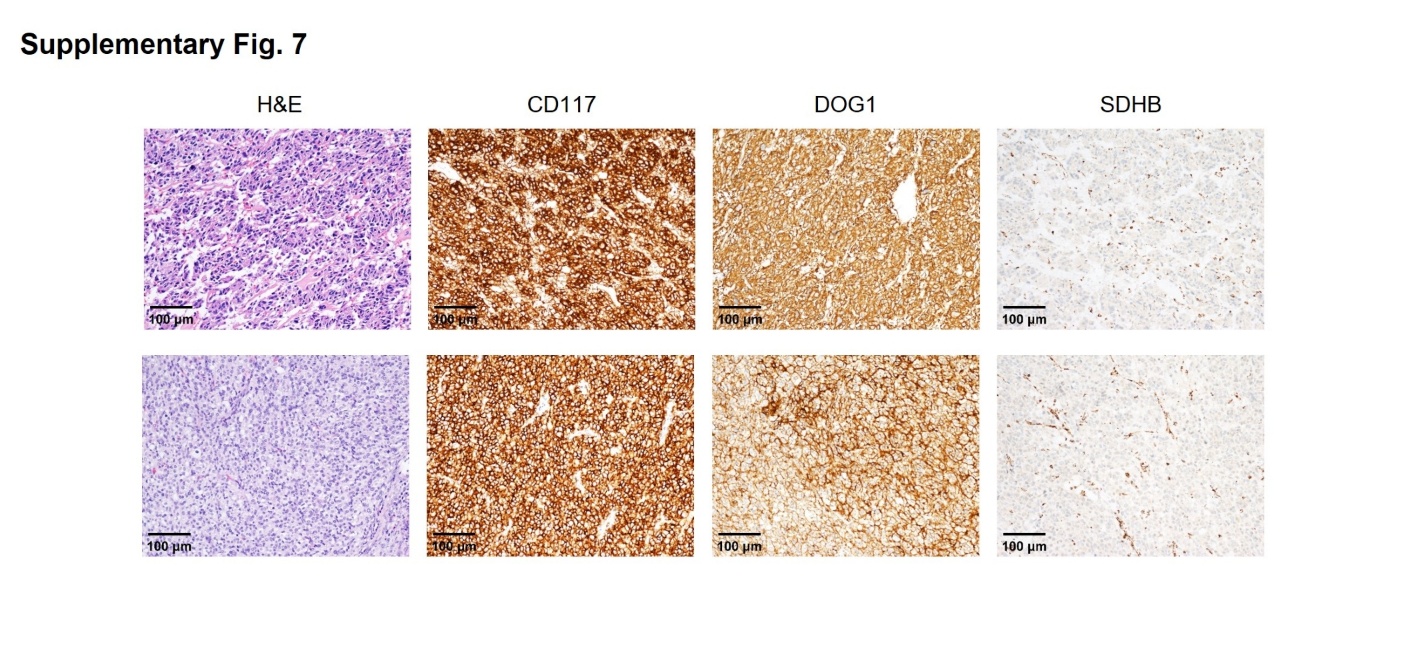
**

**Figure. S7. Representative SDHB-negative IHC staining of two cases.**

Each row is a different stain of the same sample (original magnification × 200).

**Table S1. Baseline characteristics of patients with GIST.**

|  | **Non-SDH-deficient GIST** | | **SDH-deficient GIST** | | **Total** |
| --- | --- | --- | --- | --- | --- |
| Population, n | 33 | | 26 | | 59 |
| Median age (range), y | 55.0 (33-72) | | 30.0 (13-56) | | 45.0 (13-72) |
| Sex, n (%) |  | |  | |  |
| Male | 26 (78.8) | | 7 (26.9) | | 33 (55.9) |
| Female | 7 (21.2) | | 19 (73.1) | | 26 (44.1) |
| Primary tumor site, n (%) |  | |  | |  |
| Stomach | 6 (18.2) | | 24 (92.3) | | 30 (50.8) |
| Small intestine ^a^ | 18 (54.5) | | 0 | | 18 (30.5) |
| Large intestine ^b^ | 2 (6.1) | | 0 | | 2 (3.4) |
| Other | 7 (21.2) | | 0 | | 7 (11.9) |
| Unknown | 0 | | 2 (7.7)^c^ | | 2 (3.4) |
| ECOG PS, n (%) |  | |  | |  |
| 0 | 12 (36.4) | | 11 (42.3) | | 23 (39.0) |
| 1 | 18 (54.5) | | 13 (50.0) | | 31 (52.5) |
| 2 | 3 (9.1) | | 0 | | 3 (5.1) |
| Missing | 0 | | 2 (7.7) | | 2 (3.4) |
| No. of previous TKIs, n (%) |  | |  | |  |
| 0 | 0 | | 1 (3.8) | | 1 (1.7) |
| 1 | 5 (15.2) | | 8 (30.8) | | 13 (22.0) |
| 2 | 15 (45.5) | | 4 (15.4) | | 19 (32.2) |
| 3 | 13 (39.4) | | 11 (42.3) | | 24 (40.7) |
| ≥ 4 | 0 | | 2 (7.7) | | 2 (3.4) |
| Gene mutations, n (%) | *KIT* | 29/33 (87.9) | *SDHA* | 1/26 (3.8) |  |
|  | *PDGFRA* | 2/33 (6.1) | *SDHB* | 12/26 (46.2) |  |
|  | Wild-type | 2/33 (6.1) | *SDHC* | 1/26 (3.8) |  |
|  | 1 SDH competent, NF1 positive; 1 unknown (tissue not available) | | No SDH mutation | 12/26 (46.2) |  |
| Data are median (range) or n (%). Certain proportions do not sum to 100% because of rounding. ^a^Small intestine, duodenum, jejunum, or ileum. ^b^Colon and rectum. ^c^Unknown: one was multiple liver metastases; another was umbilical masses. Abbreviations: ECOG PS, Eastern Cooperative Oncology Group performance status; GIST, gastrointestinal stromal tumour; TKI, tyrosine kinase inhibitor. | | | | | |

**Table S2. Treatment-related adverse events in all patients.**

|  | **20 mg (n = 3)** | | **30 mg (n = 15)** | | **40 mg (n = 32)** | | **50 mg (n = 16)** | | **Total (n = 66)** | |
| --- | --- | --- | --- | --- | --- | --- | --- | --- | --- | --- |
|  | **Any grade** | **Grade 3-5** | **Any grade** | **Grade 3-5** | **Any grade** | **Grade 3-5** | **Any grade** | **Grade 3-5** | **Any grade** | **Grade 3-5** |
| TRAEs, n (%) | 3 (100.0) | 0 | 13 (86.7) | 2 (13.3) | 31 (96.9) | 4 (12.5) | 15 (93.8) | 3 (18.8) | 62 (93.9) | 9 (13.6) |
| Terms reported in ≥10% of patients, n (%) |  |  |  |  |  |  |  |  |  |  |
| Leukocyte count increased | 1 (33.3) | 0 | 6 (40.0) | 0 | 12 (37.5) | 0 | 8 (50.0) | 0 | 27 (40.9) | 0 |
| AST increased | 0 | 0 | 5 (33.3) | 0 | 12 (37.5) | 0 | 6 (37.5) | 1 (6.3) | 23 (34.8) | 1 (1.5) |
| Constipation | 1 (33.3) | 0 | 4 (26.7) | 0 | 13 (40.6) | 0 | 5 (31.3) | 0 | 23 (34.8) | 0 |
| Neutrophil count increased | 1 (33.3) | 0 | 6 (40.0) | 0 | 9 (28.1) | 0 | 6 (37.5) | 0 | 22 (33.3) | 0 |
| ALT increased | 0 | 0 | 4 (26.7) | 0 | 11 (34.4) | 0 | 5 (31.3) | 1 (6.3) | 20 (30.3) | 1 (1.5) |
| Pyrexia | 0 | 0 | 1 (6.7) | 0 | 14 (43.8) | 0 | 4 (25.0) | 0 | 19 (28.8) | 0 |
| Fatigue | 0 | 0 | 4 (26.7) | 0 | 9 (28.1) | 1 (3.1) | 5 (31.3) | 0 | 18 (27.3) | 1 (1.5) |
| Melanocytic nevus | 0 | 0 | 3 (20.0) | 0 | 8 (25.0) | 0 | 4 (25.0) | 0 | 15 (22.7) | 0 |
| Hypoalbuminemia | 0 | 0 | 4 (26.7) | 0 | 3 (9.4) | 0 | 3 (18.8) | 0 | 10 (15.2) | 0 |
| Hyperuricemia | 2 (66.7) | 0 | 2 (13.3) | 0 | 6 (18.8) | 0 | 2 (12.5) | 0 | 12 (18.2) | 0 |
| CRP increased | 0 | 0 | 2 (13.3) | 0 | 6 (18.8) | 0 | 3 (18.8) | 0 | 11 (16.7) | 0 |
| Blood creatinine increased | 0 | 0 | 3 (20.0) | 0 | 5 (15.6) | 0 | 1 (6.3) | 0 | 9 (13.6) | 0 |
| Anemia | 0 | 0 | 1 (6.7) | 0 | 4 (12.5) | 0 | 4 (25.0) | 0 | 9 (13.6) | 0 |
| Platelet count increased | 0 | 0 | 1 (6.7) | 0 | 4 (12.5) | 0 | 2 (12.5) | 0 | 7 (10.6) | 0 |
| Abbreviations: ALT, alanine aminotransferase; AST, aspartate aminotransferase; CRP, C-reactive protein; TRAE, treatment-related adverse event. | | | | | | | | | | |

**Table S3. Treatment-related serious adverse events in all patients.**

| Population, N | 68 |
| --- | --- |
| Treatment-related SAE, n (%) | 5 (7.4) |
| Intestinal obstruction | 2 (2.9) |
| Anemia | 1 (1.5) |
| Leukemoid reaction | 1 (1.5) |
| Arteriosclerosis coronary artery | 1 (1.5) |
| SAE, serious adverse event. | |

**Table S4. Kaplan-Meier estimates of progression-free survival of patients treated with TKIs, based on real-world medical history per treatment line.**

|  | **First-line TKI** | **Second-line TKI** | **Third-line TKI** |
| --- | --- | --- | --- |
| Population, N | 24 | 16 | 11 |
| Event, n (%) | 18 (75.0) | 11 (68.8) | 7 (63.6) |
| Censored, n (%) | 6 (25.0) | 5 (31.3) | 4 (36.4) |
| Kaplan-Meier method |  |  |  |
| Median PFS, mo. | 6.00 | 8.00 | 5.00 |
| 95% CI | 4.00-8.00 | 3.00-10.00 | 1.00-NR |
| Abbreviations: CI, confidence interval; NR, not reached; PFS, progression-free survival; TKI, tyrosine kinase inhibitor. | | | |

**Table S5. Summary of pharmacokinetic parameters of olverembatinib at steady state in patients with GIST.**

| **Dose, mg** | **Subject no.** | **T_max_, h** | **C_max_, ng/mL** | **AUC_last_, h*ng/m** | **T_1/2_, h** | **Vz/F, L** | **Cl/F, L/h** |
| --- | --- | --- | --- | --- | --- | --- | --- |
| 20 | 2 | 7 (6.00, 8.00) | 9.18 ± 0.13 | 228 ± 6.4 | 22.2 ± 0.92 | 2143 ± 17.1 | 67.2 ± 2.3 |
| 30 | 4 | 7 (4.00, 8.00) | 12.5 ± 2.9 | 307 ± 121 | 27.0 ± 15.4 | 2649 ± 877 | 87.7 ± 56.8 |
| 40 | 15 | 6 (4.00, 24.0) | 21.8 ± 9.7 | 473 ± 177 | 18.9 ± 6.4 | 2126 ± 990 | 79.9 ± 35.3 |
| 50 | 3 | 8 (6.00, 8.00) | 26.5 ± 16.0 | 518 ± 174 | 19.5 ± 6.8 | 2349 ± 942 | 87.5 ± 39.7 |
| Values are median (range) for T_max_ and mean (SD) for all others. Pharmacokinetic parameters were derived by noncompartmental analysis. Abbreviations: AUC_last_, area under the curve from time zero to the time of the last measurable concentration (T_last_); CL/F, apparent total oral clearance; C_max_, maximum observed plasma drug concentration; T_1/2_, terminal elimination half-life; T_max_, time to reach maximum observed plasma drug concentration; Vz/F, apparent volume of distribution. | | | | | | | |

**Table S6.** **Summary of previous TKI treatment history and response to olverembatinib in patients with SDH-deficient GIST.**

| **Subject ID** | **Previous treatment history** | **Dose of olverembatinib, mg** | **Best response** |
| --- | --- | --- | --- |
| 07005 | Imatinib; Famitinib; Sunitinib | 30 | SD |
| 05011 | Imatinib; Sunitinib; Monoclonal antibody targeted PD-1 | 40 | PR |
| 01044 | Imatinib; Sunitinib; Regorafenib | 40 | PR |
| 01039 | Imatinib; Sunitinib | 40 | PR |
| 01050 | Imatinib; Sunitinib; Regorafenib; Ripretinib | 40 | SD |
| 01040 | Imatinib | 50 | SD |
| 01062 | Imatinib; Ripretinib | 40 | SD |

**Table S7. Antiproliferative activity of TKIs in SDH-deficient cell lines.**

| **Cell lines** | **IC_50_ μM, mean ± SD** | | | | |
| --- | --- | --- | --- | --- | --- |
|  | ALL | RCC | Colon cancer | Rat pheochromocytoma | |
|  | Jurkat, clone E6-1 | OS-RC-2 | RKO | PC12#5F7 | |
| **SDH status*** | SDHB: missense_variant, p.A15T | SDHA: missense_variant, p.G184R;  SDHB: stop gained, p.W218Ter | SDHA: missense_variant, p.P279S | | SDHB exon 2+3 KD (heterozygote)  by CRISPR-Cas9 |
| **Compound** |  |  |  |  | |
| Olverembatinib | 0.149 ± 0.04 | 0.135 ± 0.083 | 0.129 ± 0.136 | 5.132 ± 1.950 | |
| Imatinib | > 10 | > 10 | > 10 | > 10 | |
| Ripretinib | 1.559 ± 0.294 | 8.781 ± 1.725 | 1.247 ± 0.933 | > 10 | |
| Avapritinib | > 8.071 ± 2.728 | > 10 ± 0 | > 10 ± 0 | > 10 | |
| Ponatinib | 2.136 ± 2.107 | 0.809 ± 0.443 | 0.274 ± 0.241 | 8.537 ± 3.553 | |
| Dasatinib | 2.025 ± 0.644 | 0.029 ± 0.003 | 0.382 ± 0.320 | > 10 | |
| Sunitinib | 4.289 ± 0.280 | >5.099 ± 0.207 | 3.789 ± 0.385 | > 10 | |
| Regorafenib | > 10 | 5.110 ± 0.588 | 2.145 ± 0.812 | > 10 | |
| Sorafenib | > 10 | 3.581 ± 0.255 | 3.875 ± 0.170 | > 10 | |
| Pazopanib | > 10 | > 10 | > 10 | > 10 | |
| Rogaratinib | > 10 | > 10 | > 10 | > 10 | |
| Infigratinib | 4.581 ± 0.342 | 3.166 ± 0.791 | 2.374 ± 2.311 | > 10 | |
| Pemigatinib | 4.642 ± 1.842 | > 10 | > 10 | > 10 | |
| ^*^From Crown Bioscience database. ALL, acute lymphoblastic leukemia; CRISPR, clustered, regularly interspersed short palindromic repeats; IC_50_, half-maximal inhibitory concentration; RCC, renal cell carcinoma. | | | | | |

**Table S8. SDH mutations of SDH-deficient patients #1, 2, 3, and 4.**

| **SDH-deficient Patient #** | **SDH mutation** |
| --- | --- |
| 1 | No SDH mutation was detected, but western blot showed that SDHB levels were downregulated (Supplementary Fig. 2a), and IHC results (Supplementary Fig. 2b) also displayed negative SDHB staining. |
| 2 | SDHA, c.1866G>A (p. Trp622, heterozygote) |
| 3 | SDHA, p. L452F |
| 4 | SDHB, c. 136C>T. |

**Table S9. Antiproliferative activity of TKIs in SDH-deficient primary cells.**

| **Compound/IC_50_ (µM)** | **GIST patient 1** | **GIST patient 2** | **GIST patient 3** |
| --- | --- | --- | --- |
| Olverembatinib | 1.586 | 0.8365 | 0.1994 |
| Sunitinib | 12.24 | 5.976 | 5.584 |
| Regorafenib | 2.726 | 3.761 | 7.476 |
| Rogaratinib | 23.99 | 5.331 | ─ |
| Linsitinib | 17.61 | 7.919 | 8.513 |
| Dasatinib | 17.5 | 9.181 | 3.375 |
| Imatinib | ~26.96 | 31.09 | 16.46 |
| Ponatinib | ─ | 1.893 | 0.8881 |
| Anlotinib | ─ | ─ | 4.479 |

**Table S10. Cell lines and culture conditions.**

| **Tumor type** | **Cell line** | **Culture conditions** |
| --- | --- | --- |
| Acute lymphoblastic leukemia | Jurkat, Clone E6-1 | RPMI 1640 medium supplemented with 4.5 g/L glucose, 10 mM HEPES, 1.0 mM sodium pyruvate, 10% fetal bovine serum (FBS), and 1% penicillin/streptomycin (P/S) |
| Renal cell carcinoma | OS-RC-2 | RPMI 1640 medium supplemented with 10% FBS and 1% P/S |
| Colon cancer | RKO | Minimum essential medium supplemented with 0.1 mM nonessential amino acids, 1.0 mM sodium pyruvate, 10% FBS, and 1% P/S |
| Rat pheochromocytoma | PC12 | RPMI 1640 medium supplemented with 5% FBS, 10% horse serum, and 1% P/S |
|  | PC12#5F7 | RPMI 1640 medium supplemented with 5% FBS, 10% horse serum, and 1% P/S |

HEPES, N-2-hydroxyethylpiperazine-N'-2-ethanesulfonic acid; RPMI, Roswell Park Memorial Institute.
