# Supplementary figures and images for: Olverembatinib, a multikinase inhibitor that modulates lipid metabolism, in advanced succinate dehydrogenase-deficient gastrointestinal stromal tumors: a phase 1b study and translational research
Source: Signal Transduct Target Ther. 2025 Nov 4;10:361. doi: 10.1038/s41392-025-02456-9 (PMC12583704; doi:10.1038/s41392-025-02456-9)

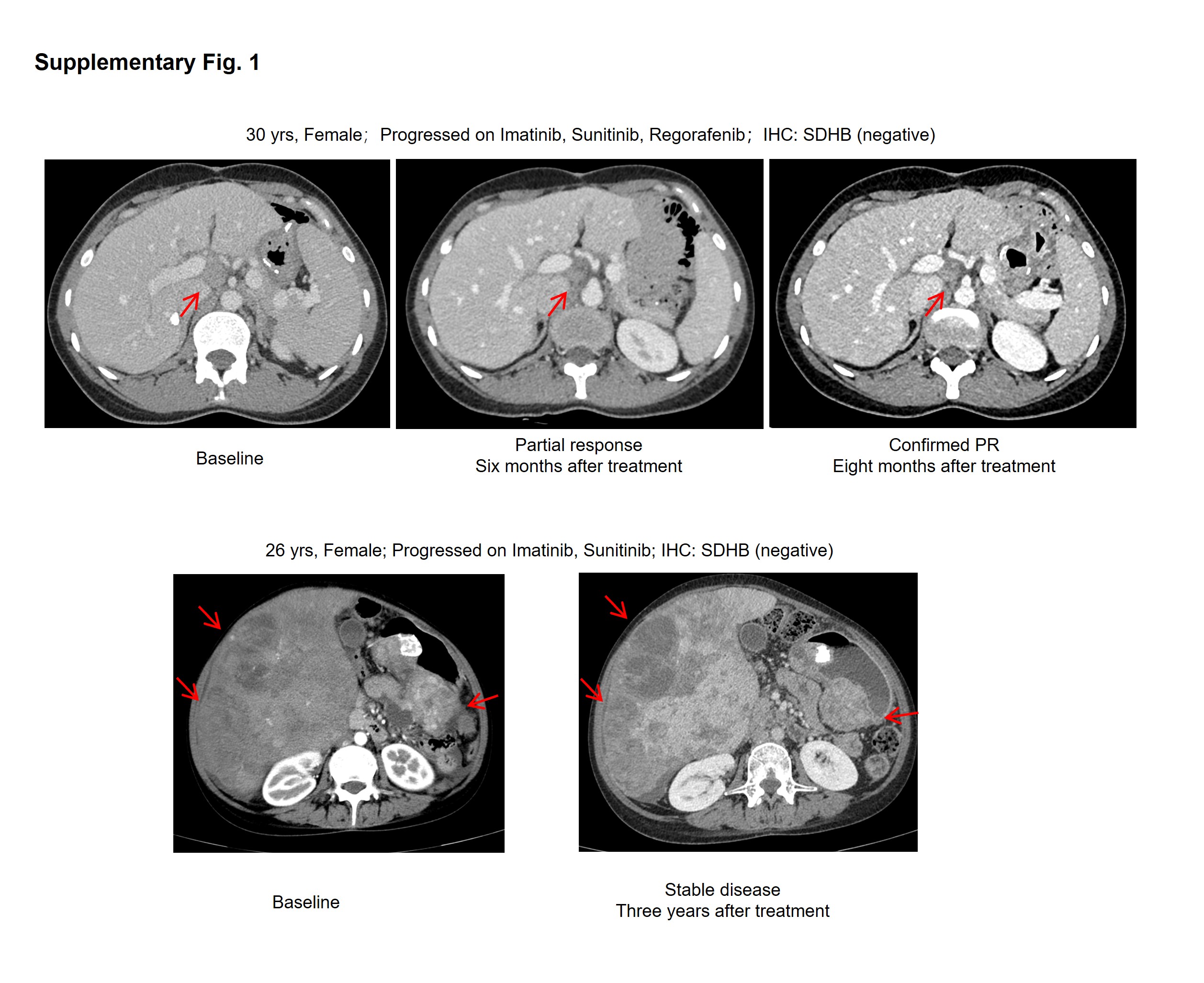

Supplement: Supplementary file 2 — Supplementary Figure 1 [file 41392_2025_2456_MOESM2_ESM.jpg]

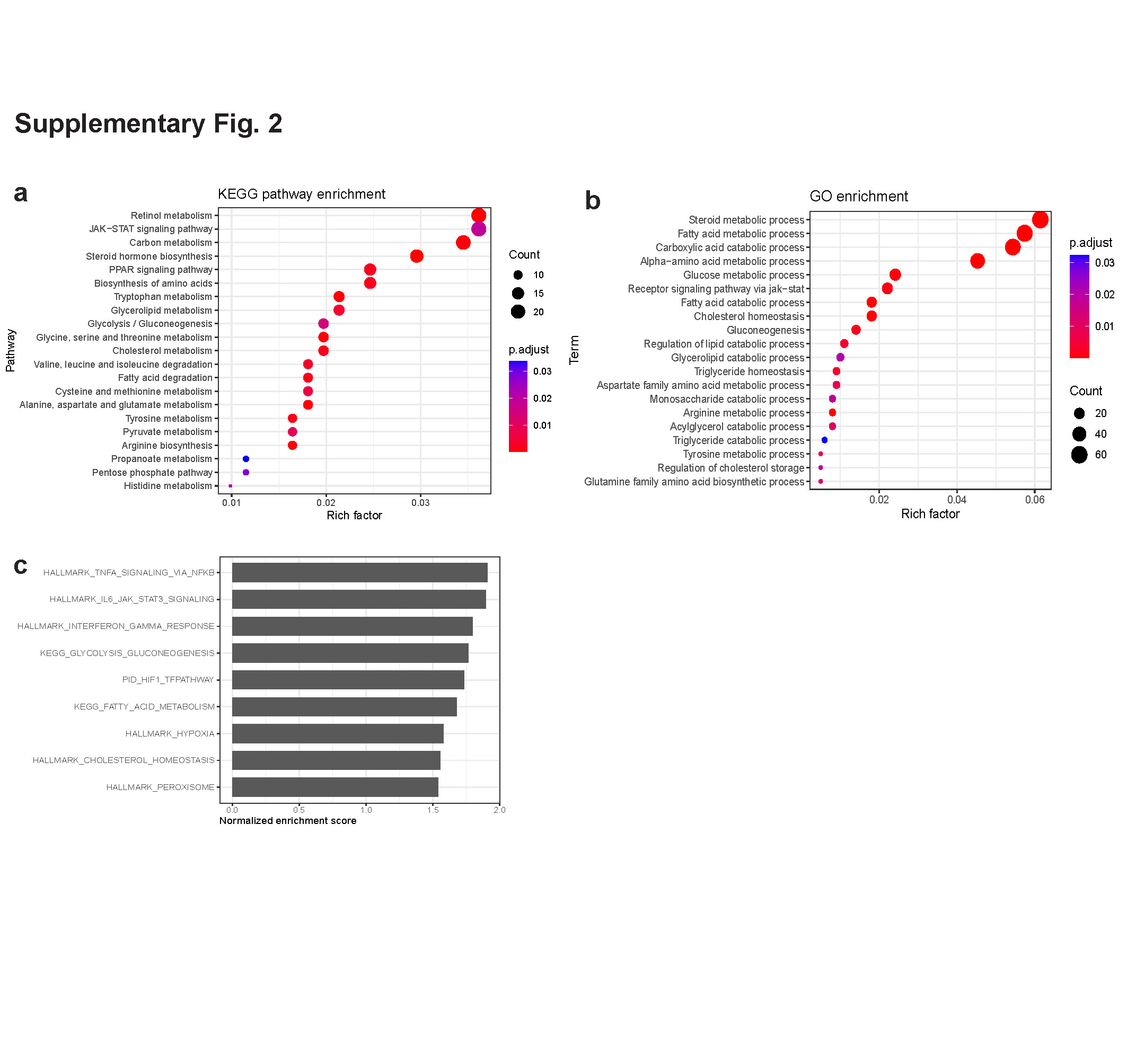

Supplement: Supplementary file 3 — Supplementary Figure 2 [file 41392_2025_2456_MOESM3_ESM.jpg]

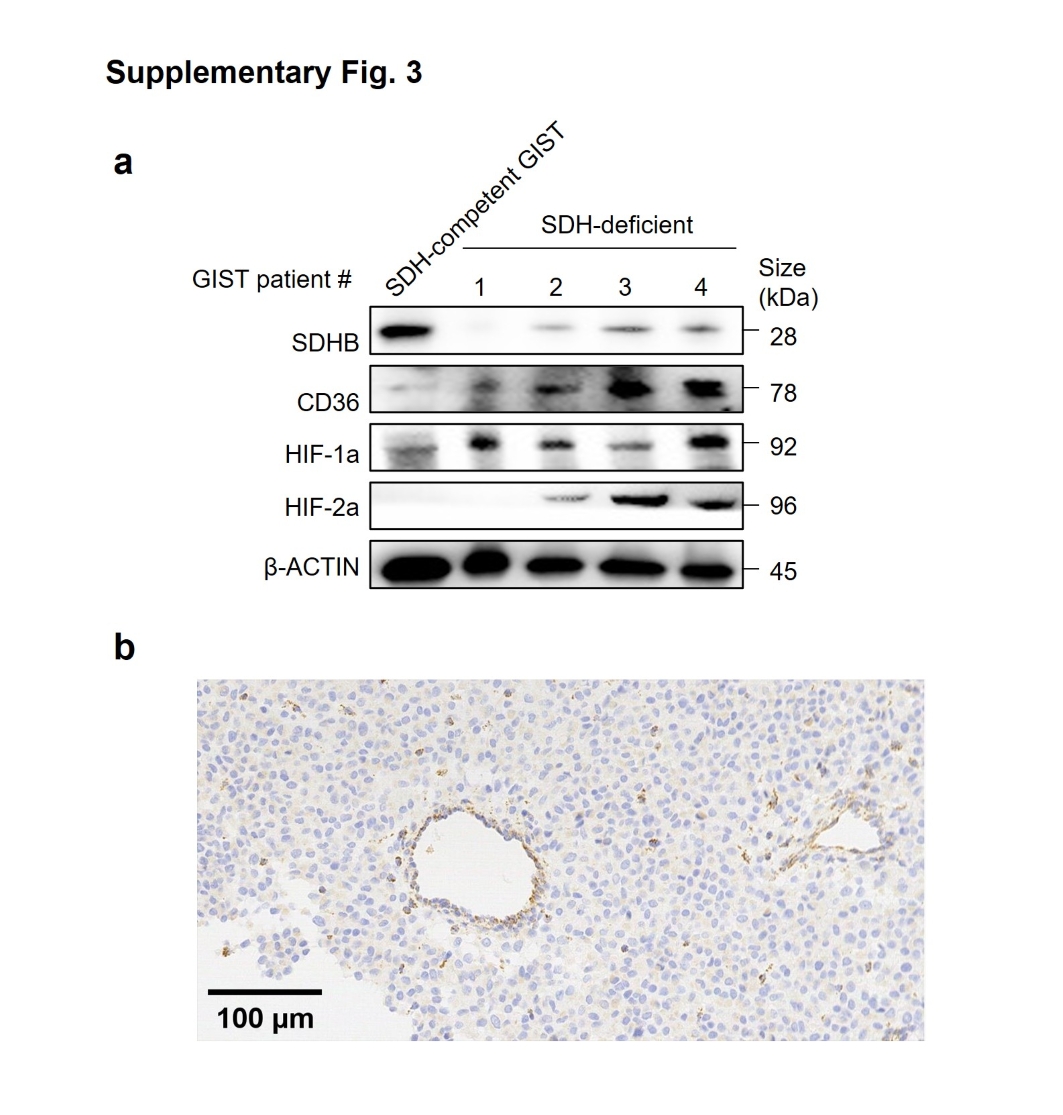

Supplement: Supplementary file 4 — Supplementary Figure 3 [file 41392_2025_2456_MOESM4_ESM.jpg]

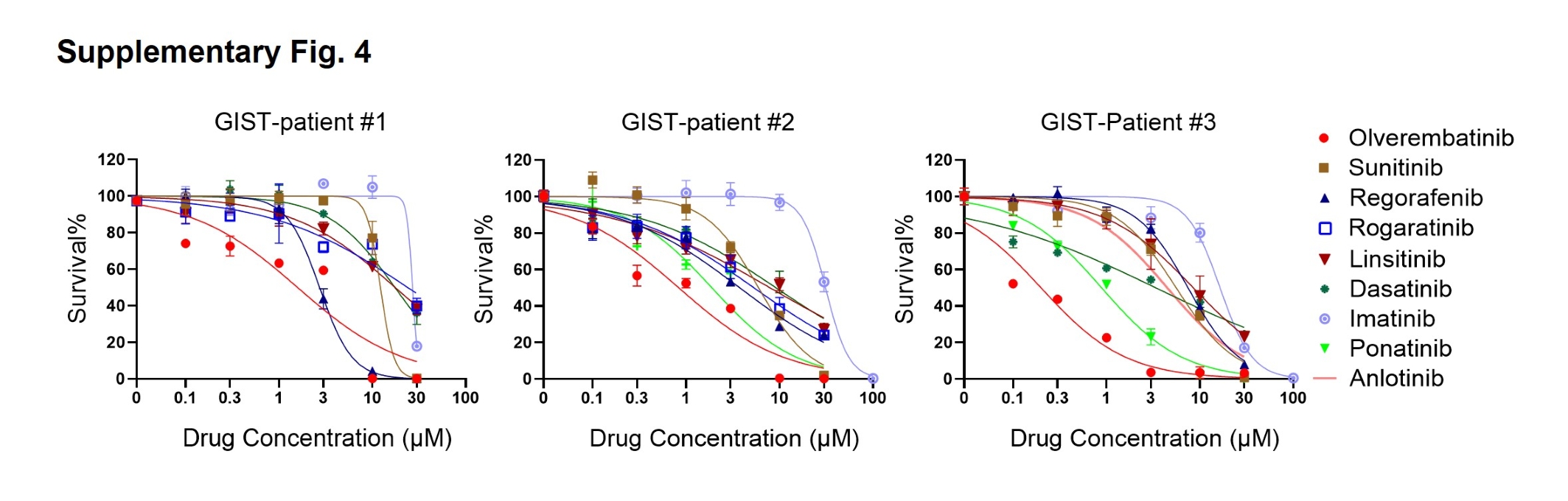

Supplement: Supplementary file 5 — Supplementary Figure 4 [file 41392_2025_2456_MOESM5_ESM.jpg]

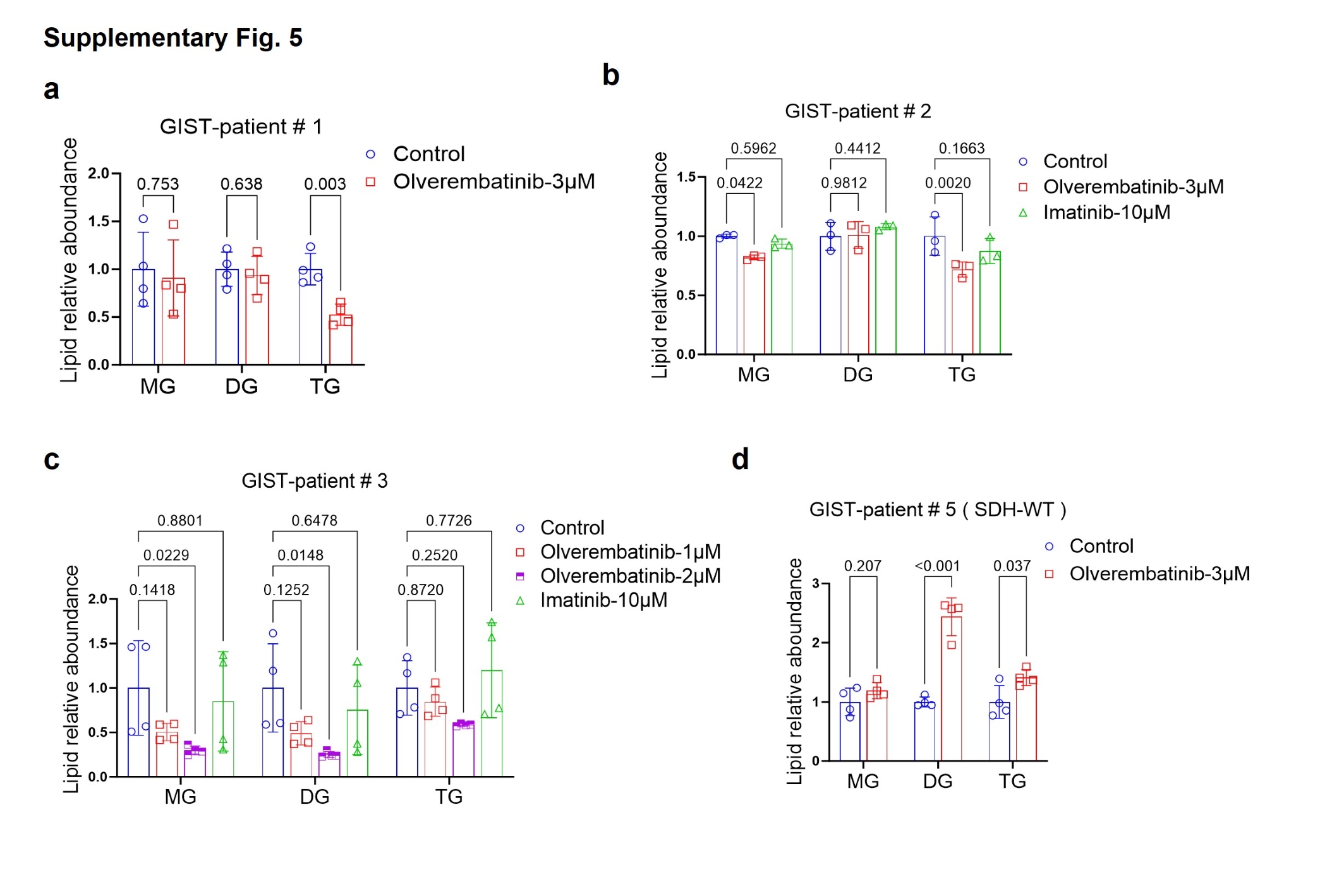

Supplement: Supplementary file 6 — Supplementary Figure 5 [file 41392_2025_2456_MOESM6_ESM.jpg]

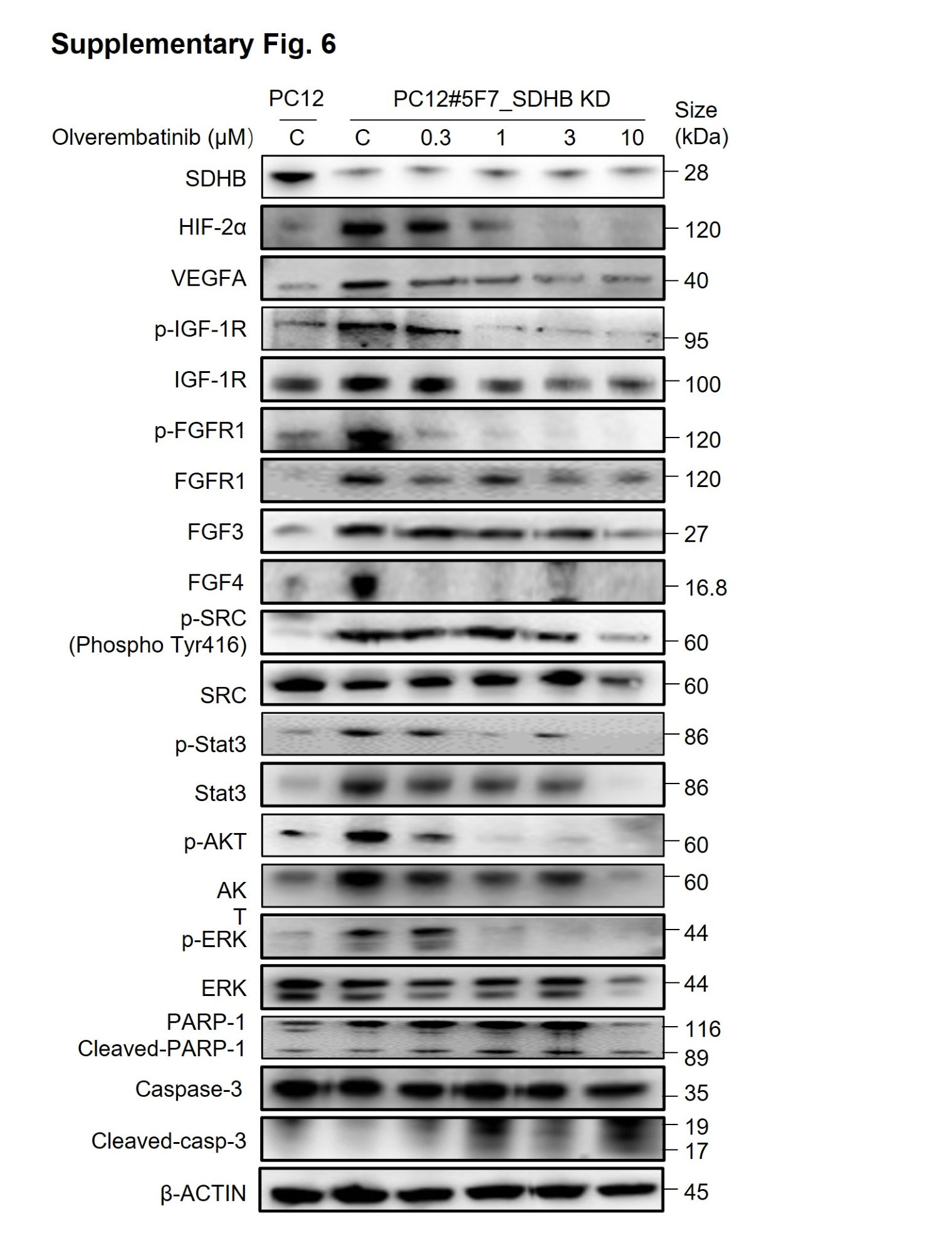

Supplement: Supplementary file 7 — Supplementary Figure 6 [file 41392_2025_2456_MOESM7_ESM.jpg]

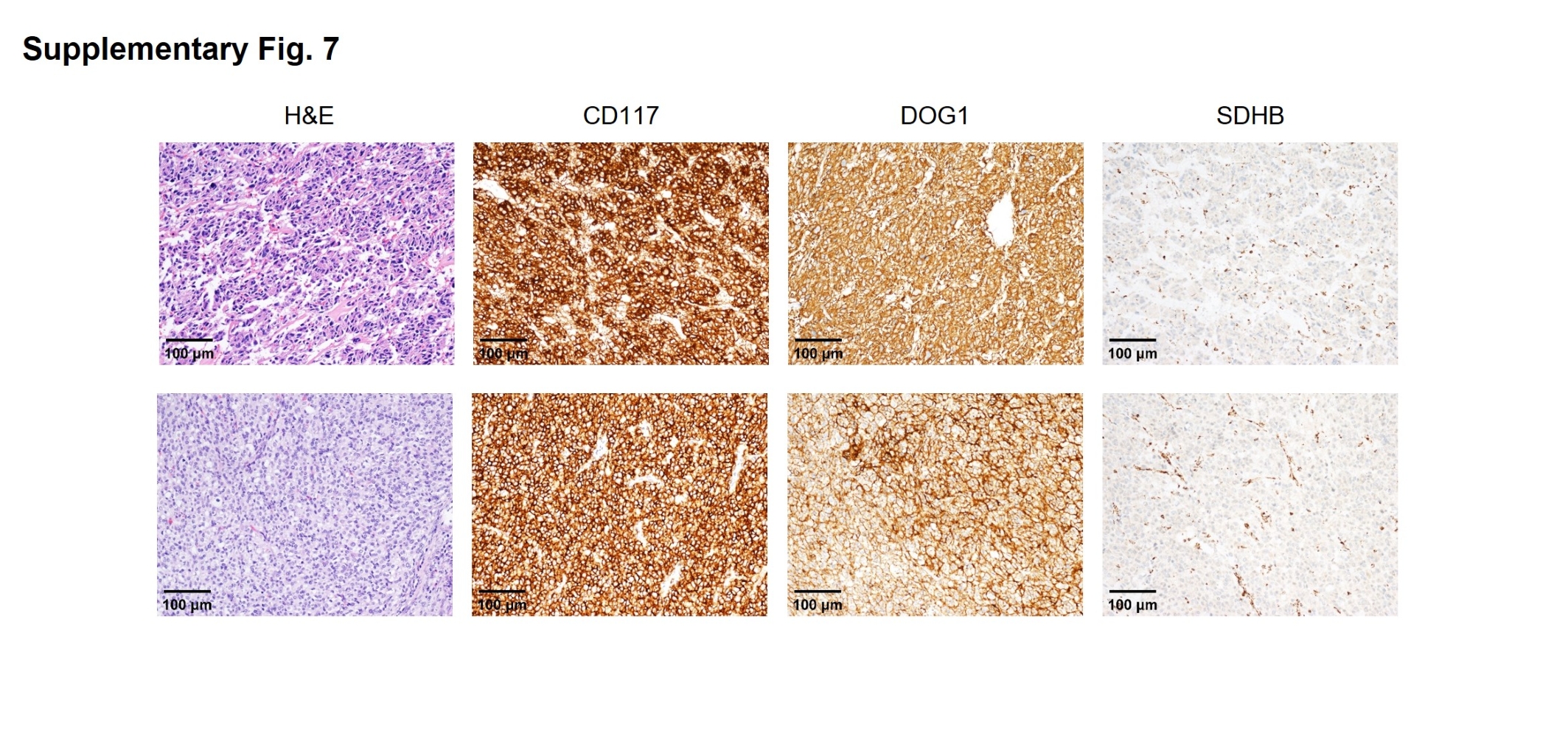

Supplement: Supplementary file 8 — Supplementary Figure 7 [file 41392_2025_2456_MOESM8_ESM.jpg]
