# Supplementary material for: Olverembatinib, a multikinase inhibitor that modulates lipid metabolism, in advanced succinate dehydrogenase-deficient gastrointestinal stromal tumors: a phase 1b study and translational research
Source: Signal Transduct Target Ther. 2025 Nov 4;10:361. doi: 10.1038/s41392-025-02456-9 (PMC12583704; doi:10.1038/s41392-025-02456-9)
Supplement: Supplementary file 9 — HQP1351 SJ0003 Protocol [file 41392_2025_2456_MOESM9_ESM.pdf]

HQP1351 Tablets

Clinical Study Plan and Protocol

**Study title:**	**A Phase I Clinical Study of the Safety, Pharmacokinetics and Pharmacodynamics of HQP1351 in Patients with Advanced Gastrointestinal Stromal Tumors and Other Solid Tumors**
**Protocol No.:**	SJ-0003
**Protocol Version:**	4.0
**Date:**	May 27, 2022

**Leading Site:**	Sun Yat-sen University Cancer Center
Address:	No. 651, Dongfeng East Road, Guangzhou, Guangdong
Principal investigator:	Professor Ruihua Xu
	Professor Zhiwei Zhou

Statement of Confidentiality

The information contained in this document is proprietary to Guangzhou HealthQuest Pharma Inc. This information should be used only for review or participating in approved activities related to the clinical study of the investigational drug as described in the protocol. Without the written approval of Guangzhou HealthQuest Pharma Inc., such information should not be disclosed to others who are not obliged to keep the information confidential, except that it is required to provide the potential subject with a general description of the protocol, to obtain his/her informed consent.

Emergency Contact Person

Table 1: Emergency Contact Information

Responsibilities in the study	Name, position	Contact information
24-Hour emergency contact person	Dr. Yifan Zhai<br>CEO/ President	Phone number: 18998334688<br>Fax: +86-20-2806 8500
Drug safety physician	Dr. Zi Chen<br>SVP of Clinical Development	Phone number: 18117275173<br>Fax: +86-20-2806 8500
Contact person supporting clinical study	Dr. Dajun Yang<br>Chairman	Phone number: 13661838118

Statement and Signature of the Sponsor

We will conscientiously perform our duties according to relevant regulations in *Good Clinical Practice*.

Sponsor: Guangzhou HealthQuest Pharma Inc.

Address: Room 515, Building D, No. 3, Lanyue Road, Huangpu District, Guangzhou, Guangdong;

Postcode: 510700

Tel.: (020)28068500 Fax: (020)28068525

Signature of Responsible Person of the Sponsor

Dr. Yifan Zhai

CEO/ President

Signature: ____________________ Date: DD/MM/YYYY

Statement and Signature of Principal Investigator

I will earnestly perform the investigator's responsibility according to relevant regulations of *Good Clinical Practice*, and conduct the clinical trial in compliance with the protocol.

I will, in accordance with the procedures specified in China's GCP, record the clinical trial data as required in this protocol, and ensure that the data of this clinical trial are entered into case report forms in a real, accurate, timely and legal manner. I agree to accept the monitoring or auditing by the clinical research associate (CRA) or auditor dispatched by the Sponsor or the inspection by the drug regulatory authorities to ensure the quality of clinical trial.

I will be responsible for making medical decisions related to the clinical trial to ensure that patients are treated as soon as possible for adverse events occurring during the trial. I have understood the correct procedures and requirements for reporting of serious adverse events and if adverse events or serious adverse events have been identified in the clinical study, I will report them in accordance with the requirements in the protocol.

Study site:

Address:

Postcode:

Principal Investigator

Name: ....(Print)

Signature: ....   Date: DD/MM/YYYY

Synopsis

**Sponsor/Company:**<br>Guangzhou HealthQuest Pharma Inc.
**Investigational drug:**<br>HQP1351, olverembatinib
**Chemical name of investigational drug:**<br>3-[(1H-pyrazolo[3,4-b]pyridin-5-yl)ethynyl]-4-methyl-N-{4-[(4-methylpiperazin-1-yl)methyl]-3-(trifluoromethyl)phenyl]benzamide
**Study Title:**<br>A Phase I Clinical Study of the Safety, Pharmacokinetics and Pharmacodynamics of HQP1351 in Patients with Advanced Gastrointestinal Stromal Tumors and Other Solid Tumors
**Study site: Multicentre**
**Estimated recruitment duration (year):**<br>4 to 5 years
**Phase:**<br>Phase I
**Objectives:**<br>**Primary objective:**<br>• To assess the safety and tolerability of HQP1351 in different dose groups in the treatment of patients with advanced gastrointestinal stromal tumors (GIST) or other solid tumors, and determine the recommended Phase II dose (RP2D).<br>**Secondary objectives:**<br>• To preliminarily assess the efficacy of HQP1351 in patients with advanced GIST or other solid tumors.<br>• To assess the pharmacokinetics (PK) of HQP1351.<br>**Exploratory objective:**<br>• To explore biomarkers related to the efficacy of HQP1351 monotherapy.
**Study design:**<br>This is an open-label, multicenter, Phase I clinical trial which is designed to determine the safety and tolerability of HQP1351 in patients with advanced GIST and other solid tumors,

to determine the RP2D, and to assess the preliminary efficacy of HQP1351 in patients with GIST and other solid tumors.

As of February 2021, HQP1351 has undergone a total of 9 clinical studies, and preliminary results from the Phase I and Phase II clinical trials that have been conducted show a favorable safety profile up to dose of 50 mg QOD. HQP1351 (olverembatinib tablets) has been granted conditional marketing authorization from the National Medical Products Administration on November 24, 2021 (approval number: NMPA H20210048). It is indicated for the treatment of "adult patients with tyrosine kinase inhibitor (TKI)-resistant chronic phase chronic myeloid leukemia (CML-CP) or accelerated-phase CML (CML-AP) harboring the T315I mutation as confirmed by a validated diagnostic test", and the recommended dose is 40 mg QOD.

This study is initially designed as a Phase I study with standard 3+3 dose escalation. Based on the study results of HQP1351 SJ-0002 in CML patients and the consensus reached at the SJ-0003 kick-off meeting with the investigator, this study will explore the safety in the 30 mg, 40 mg, and 50 mg dose groups in parallel, and 10 subjects will be enrolled by means of randomization at each dose level using the IWRS. After preliminary adult data are available, adolescent subjects with advanced and/or metastatic wild-type GIST will be included. The dosage for adolescents is based on the adult dose and the selected dose will be adjusted according to their body weight. If the subject's body weight is ≥ 40 kg, the dose of 40 mg QOD will be given based on the adult dose. If the subject's body weight is ≥ 30 kg and < 40 kg, the dose of 30 mg QOD will be given. If the subject's body weight is ≥ 20 kg and < 30 kg, 20 mg QOD will be given. Based on the safety data from 9 clinical studies that have been conducted with HQP1351 and the safety and efficacy data of 36 patients already enrolled in this study, 40 mg QOD is selected as the dosage for non-randomized adult subjects after discussion between the investigator and the sponsor.

Subjects enrolled in the Phase I study with standard 3+3 dose escalation who have received at least one cycle of HQP1351 after this protocol amendment takes effect, and have not experienced related or possibly related Grade ≥ 2 toxicity nor have tumor progression, may receive a higher dose of HQP1351 upon their own informed consent after discussion between the investigator and the sponsor. The subject will continue to be followed up according to the revised protocol, but will not be included in the number of randomized subjects.

Eligible subjects after screening will orally receive HQP1351 tablets every 2 days continuously in 28-day cycles until PD, intolerable toxicity, death, or termination of treatment for any other reason. If a subject is assessed to have PD according to the RECIST v1.1 criteria, but the investigator determines that the subject will benefit from continuing the study, then the subject may continue to take HQP1351 and undergo safety follow-up yet no more efficacy follow-up after PD is documented. During medication, subjects will not

receive other antitumor study drugs, marketed drugs, or treatment methods other than HQP1351. Symptomatic (including those directed at controlling symptoms resulting from the patient's malignancy) and supportive treatments are allowed.

In the standard 3+3 dose escalation part of this study, blood samples from subjects in Cycle 1 will be collected for the PK analysis of HQP1351. In the randomized part, 1-3 study sites will be selected to collect the blood samples of the first 3 enrolled subjects in each dose group in Cycle 1 for the PK analysis of HQP1351. If for any reason complete PK samples are not obtained from the first 3 subjects, they will be replaced by subjects subsequently enrolled in the dose group until complete PK samples are obtained from 3 subjects. PK blood samples will be collected from all non-randomized adult subjects receiving a fixed dose of 40 mg QOD for PK analysis. PK blood samples will be collected from all adolescent subjects for PK analysis.

Blood and tumor tissue samples will be collected from all subjects for biomarker assessment.

All subjects will undergo efficacy assessment every 8 weeks during treatment, along with assessment of safety information such as AEs and laboratory tests.

Number of subjects (planned):

About 50-60 subjects are expected to be included in this study.

Inclusion criteria:

Subjects will be eligible for the study if they meet all of the following criteria:

1. Aged ≥ 12 years, male or female.
2. Advanced and/or metastatic GIST or other solid tumors, confirmed by histology and/or cytology. Among them, GIST patients must have primary resistance to imatinib (tumor progression within 6 months after first-line use of imatinib, or immunohistochemical presence of SDHB expression deletion or NF1 mutation) or have failed treatment with imatinib or imatinib plus at least one other TKI (following initial imatinib or other TKI treatment for more than 6 months, the subject has tumor progression again after achieving a response or stable disease).
3. ECOG score ≤ 2.
4. Life expectancy of ≥ 3 months.
5. Hematologic function as outlined below:
    - Absolute neutrophil count (ANC) ≥ 1.5 $\times 10^9$/L (ANC achieved by colony-stimulating factors will not be allowed);
    - Platelet count ≥ 100 × 10^9/L (baseline platelet count achieved by blood transfusion or thrombopoietic growth factor will not be allowed);

- Hemoglobin ≥ 90 g/L

  Note: If hemoglobin is stable for 14 days after transfusion (reduced by no more than 10 g/L), transfusion is allowed, and the use of erythropoiesis-stimulating agents (ESAs) is allowed;
- Serum albumin ≥ 30.0 g/L;
- Serum lipase ≤ 1.5 × upper limit of normal (ULN);
- Serum amylase ≤ 1.5 × ULN;

6. Hepatic and renal function:
   - Serum creatinine ≤ 1.5 × ULN; or serum creatinine > 1.5×ULN, and creatinine clearance ≥ 50 mL/min;
   - Serum total bilirubin ≤ 1.5 × ULN;
   - Aspartate aminotransferase (AST) and alanine aminotransferase (ALT) ≤ 2.5 × ULN (≤ 5 × ULN in the presence of liver metastasis);
7. Cardiac function index:
   - Troponin (I or T) ≤ ULN;
   - Ejection fraction (EF) > 40%;
   - QTc interval on electrocardiogram (corrected by Bazett's formula): male ≤ 450 ms and female ≤ 470 ms.
8. Women of childbearing potential should have a negative serum pregnancy test within 7 days prior to the first dose of the study drug.
9. Men and women of childbearing potential (postmenopausal women must be amenorrheic for at least 12 months to be considered of non-childbearing potential) and their partners are willing to take protocol-specified effective contraceptive measures from signing the informed consent from to at least 30 days after the last dose of the investigational product.
10. Able to understand and voluntarily sign the written ICF before any study-specific procedures.
11. Subjects must be willing and able to complete study procedures and follow-up examination.

Exclusion criteria:

Subjects who meet any of the following criteria are not eligible for this study:

1. Subjects who have received antitumor cytotoxic chemotherapy, biologic drug therapy (such as monoclonal antibodies), immunotherapy (such as interferon), or radiotherapy

within 28 days prior to the first dose or less than 5 times the half-life.

2. Subjects who have received tyrosine kinase inhibitor (TKI) treatment within 14 days prior to the first dose.
3. Subjects who have received other study drugs within 14 days prior to the first dose.
4. Subjects who have not recovered from adverse events (except alopecia) (> NCI-CTCAE v4.03 grade 1) due to prior treatment.
5. Malabsorption syndrome or other diseases affecting oral drug absorption.
6. Clinically significant, uncontrolled, or active cardiovascular diseases, including but not limited to history of myocardial infarction; history of unstable angina; history of congestive heart failure or left ventricular ejection fraction (LVEF) below the lower limit of normal within 6 months; history of atrial arrhythmia judged by the investigator to be of important clinical significance; history of ventricular arrhythmia, etc.
7. Hypertension inadequately controlled by antihypertensive drugs (systolic pressure >150 mmHg or diastolic pressure >90 mmHg).
8. Subjects who need to take drugs that has a known potential to cause ECG QT prolonged.
9. Pulmonary arterial systolic pressure >35 mmHg as indicated by Echocardiography.
10. Subjects who have serious cardiovascular diseases during prior TKI use.
11. Hypertriglyceridemia that is difficult to control.
12. Major surgery, open biopsy (except intravenous catheter placement or bone marrow biopsy), or major traumatic injury within 14 days prior to the administration of study drug.
13. Arterial thrombosis or embolic events such as cerebrovascular accidents (including transient ischemic attacks), or pulmonary embolism or venous thrombosis events within 6 months prior to the administration of study drug, such as deep vein thrombosis within 3 months prior to the administration of study drug.
14. Subjects who require concomitant immunosuppressive therapy, other than corticosteroids prescribed for a short course of therapy.
15. Subjects with brain metastases.
16. Patients with other primary malignant neoplasm within recent 3 years (patients who are cured for ≥ 5 years, or with completely resected non-melanoma skin cancer, or successfully treated carcinoma in situ, or prostatic carcinoma under control will be included).
17. Active symptomatic viral infections (including known HIV infections, viral hepatitis (B or C, etc.), syphilis, etc.).

18. Subjects who are known to be allergic to any components of the investigational drug or its analogues.
19. Female subjects who are pregnant or breastfeeding, or who expect to become pregnant during the study period.
20. Subjects who have any symptom or disease that, according to the opinions of the investigator or sponsor, would comprise their safety or interfere with the safety assessment of the investigational drug.

Dosing regimen

HQP1351 Tablets will be orally administered at a dose of 10 mg/tablet every two days in a 28-day cycle. Randomized subjects will randomly receive 30 mg QOD, 40 mg QOD, and 50 mg QOD dosing regimens. Non-randomized adult subjects will receive a 40 mg QOD dosing regimen. The dosage for adolescents is based on the adult dose and the selected dose will be adjusted according to their body weight. If the subject's body weight is ≥ 40 kg, the dose of 40 mg QOD will be given based on the adult dose. If the subject's body weight is ≥ 30 kg and < 40 kg, the dose of 30 mg QOD will be given. If the subject's body weight is ≥ 20 kg and < 30 kg, 20 mg QOD will be given.

Duration of treatment:

Subjects will receive treatment until PD, observation of intolerable toxicity, death, or termination of treatment for any other reason. If a subject is assessed to have PD according to the RECIST v1.1 criteria, but the investigator determines that the subject will benefit from continuing the study, then the subject may continue to take HQP1351 and undergo safety follow-up yet no more efficacy follow-up after PD is documented.

Study endpoints

Primary endpoints:

- Safety and tolerability assessment will include AEs, physical examination, vital signs, electrocardiogram (ECG) parameters, ECOG performance status score, clinical laboratory tests, and other data.

Secondary endpoints:

- To preliminarily assess the efficacy of HQP1351 in patients with gastrointestinal stromal tumors, including overall response rate (investigator-assessed ORR, as per RECIST 1.1), clinical benefit rate (CBR, defined as CR+PR+SD ≥ 16 weeks), duration of response (DOR), progression-free survival (PFS), and overall survival (OS).
- To assess the pharmacokinetics (PK) of HQP1351.

Exploratory endpoint:

- Mutations of *KIT, PDGFRa*, and other tumor-related genes and/or expression profiles associated with the efficacy of HQP1351.

Statistical Methods:

The latest version of SAS software will be adopted to summarize and tabulate data. Appropriate descriptive statistics (i.e., mean, standard deviation, median and interquartile range, etc.) will be used based on data distribution. Changes from baseline in safety and tumor efficacy measures at each time point during the treatment period and the follow-up period will be compared and assessed. If applicable, the difference in dose level will be analyzed by parametric or non-parametric significance tests.

PK analysis

Appropriate, standard nonlinear analysis software (e.g., Pharsight Corporation WinNonlin®) will be used to analyze PK parameters such as total clearance (CL), half-life ($t_{1/2}$), apparent volume of distribution (V_d), steady-state blood concentration (C_{ss}), area under the blood concentration-time curve (AUC), and peak blood concentration (C_{max}), etc.

Efficacy Analyses

All efficacy evaluations (ORR, CBR, DOR, PFS, and OS) will be analyzed according to the investigator's evaluation. Assessments are required to be performed in accordance with RECIST v1.1. The ORR for all responses (including partial response (PR) and complete response (CR)) must be confirmed by a repeat assessment not less than 4 weeks and theoretically not later than 5 weeks after the response criteria are met for the first time. All efficacy analyses will be based on the Full Analysis Set (FAS), and efficacy analyses based on the Per Protocol Set (PPS) will also be performed as supporting analysis data. All efficacy data will be listed.

Table of Contents

Synopsis 5
Table of Contents 12
List of Abbreviations and Definitions of Terms 19
1 Study Background 21
1.1 Pre-Clinical Pharmacodynamic Studies 22
1.2 Safety Pharmacology Studies 25
1.3 Pre-clinical Pharmacokinetic Studies and Metabolism Studies 25
1.4 Preclinical Toxicology Studies 27
1.5 HQP1351 Clinical Overview 28
1.5.1 Clinical pharmacokinetics 28
1.5.2 Clinical safety 29
1.6 Risk-benefit and Ethical Assessment 30
2 Study Objectives 32
2.1 Primary Objective 32
2.2 Secondary Objectives 32
2.3 Exploratory Objective 32
3 Trial Population 33
3.1 Inclusion Criteria 33
3.2 Exclusion Criteria 34
3.3 Discontinuation of Study Treatment/Withdrawal from the Study 35
3.3.1 Termination of study treatment 35
3.3.2 Withdrawal from the Study 36
3.4 Patient Re-screening Policy 36
3.5 Site Closure 36
4 Clinical Study Plan 37
4.1 Study Design 37
4.2 Intolerable Toxicity 38
4.3 Dose Delay and/or Dose Reduction 39
4.3.1 Dose interruption 39

4.3.2 Dose adjustment 39

4.3.3 Principles for management of serum troponin increased 43

4.3.4 Treatments for patients who develop allergic reactions on or after Day 1 43

4.3.5 Guidance for hypertension management during study drug administration 45

4.4 End of Study and Efficacy and Safety Analysis 45

4.5 Premature Termination of Study 45

5 Study Drug 46

5.1 CMC Introduction to HQP1351 46

5.1.1 Study drug materials and dosage forms 46

5.1.2 Packaging and labeling 46

5.1.3 Storage and handling 46

5.1.4 Usage 46

5.2 Drug Management 46

5.3 Treatment Compliance 47

5.3.1 Subject diary 47

5.3.2 Tablet counting 47

5.4 Randomization and Blinding 47

5.5 Protocol Deviations 48

6 Prophylactic Medications and Concomitant Medications/Treatments 49

6.1 Pre-treatment Drugs 49

6.2 Concomitant Medications and Concomitant Therapies 49

6.3 Prohibited Medications and Therapies 49

6.4 Potential Drug Interactions 50

7 Assessment Schedule and Procedure of the Study 51

7.1 Informed Consent 51

7.2 Subject Enrollment and Allocation to Treatment 52

7.2.1 Subject screening number and randomization number 52

7.2.2 Subject screening visit 52

7.2.3 Information collection from screen failures 54
7.3 Cycle 1 Visit 54
7.3.1 C1D1 visit 54
7.3.2 C1D2 visit (only for subjects with PK blood samples collected) 55
7.3.3 C1D3 visit (only for subjects with PK blood samples collected) 55
7.3.4 C1D8 visit 55
7.3.5 C1D15 visit 56
7.3.6 C1D17 (only for subjects with PK blood samples collected) 56
7.3.7 C1D19 (only for subjects with PK blood samples collected) 56
7.3.8 C1D22 visit 57
7.3.9 C1D27 visit (only for subjects with PK blood samples collected) 57
7.3.10 C1D28 visit (only for subjects with PK blood samples collected) 57
7.4 Cycle 2 Visit 57
7.4.1 C2D1 visit 57
7.4.2 C2D8 visit 58
7.4.3 C2D15 visit 58
7.4.4 C2D22 visit 59
7.5 Visit on Cycle 3 and Subsequent Cycles 59
7.5.1 Day 1 visit of Cycle 3 and subsequent cycles 59
7.5.2 C3D15 and C4D15 visits 60
7.6 EOT Visit 60
7.7 EOT Visit ....**Error! Bookmark not defined.**
7.7.1 Day 30 visit after the last dose 61
7.7.2 Efficacy follow-up and survival follow-up after the EOT 61
7.8 PK Studies 62
7.9 Exploratory Biomarker Study 63
8 Efficacy Assessments 68
8.1 Response Evaluation Criteria in Solid Tumors (RECIST) 68
8.1.1 Measurable lesions 68
8.1.2 Non-measurable lesions 68

8.1.3 Special considerations regarding lesion measurability 69
8.1.4 Measurement of lesions 69
8.1.5 Method of assessment 70
8.1.6 Tumor response evaluation 71
8.1.7 Response criteria 72
8.1.8 Evaluation of non-target lesions 73
8.1.9 Evaluation of best overall response 75
8.1.10 Frequency of tumor re-evaluation 77
8.1.11 Response confirmation 78
9 Adverse events (AEs) and serious adverse events (SAEs) 79
9.1 Definitions 79
9.1.1 Adverse events (AEs) 79
9.1.2 Serious adverse events (SAEs) 79
9.2 Collection and Reporting of AEs 80
9.2.1 Time frame for collection of AEs 80
9.2.2 Follow-up of AEs 80
9.2.3 AEs based on examinations and tests 80
9.2.4 Treatment failure or PD 81
9.2.5 Severity grading of AEs 81
9.2.6 Correlation between an AE and the study drug 82
9.3 Reporting SAEs 82
9.4 Reporting Requirements after the End of Clinical Trial 83
9.5 Guideline for Supportive Therapy 83
9.6 Overdose 84
9.6.1 Definition of overdose 84
9.6.2 Reporting drug overdoses to the sponsor 84
9.7 Pregnancy 84
9.7.1 Period of pregnancy information collection 84
9.7.2 Measures to be taken if pregnancy occurs 84

9.7.3 Measures to be taken if a male patient's female partner becomes pregnant 85

9.7.4 Warnings and precautions for use.....85

10 Statistical Analysis.....86

10.1 Sample Size Calculation.....86

10.2 Study Endpoints.....86

10.2.1 Primary endpoints.....86

10.2.2 Secondary endpoints.....86

10.2.3 Exploratory endpoints.....87

10.3 Analysis Sets.....87

10.4 Statistical Analysis Method.....88

10.4.1 Demographic and baseline data.....88

10.4.2 Efficacy analyses.....88

10.4.3 Safety data.....88

10.4.4 PK analysis.....89

11 Quality Control and Management.....90

11.1 Quality Control and Assurance.....90

11.2 Monitoring of Clinical Study.....90

11.3 Quality Management Examinations.....90

12 Ethical Requirements.....92

12.1 Independent Ethics Committee (IEC).....92

12.2 Ethical Conduct of the Study.....92

12.3 Written Informed Consent.....92

13 Data Processing and Document Retention.....94

13.1 Case Report Form (CRF).....94

13.2 Data Collection.....94

13.3 Preservation of Original Documents.....95

13.4 Record Retention.....95

14 Responsibilities of All Parties and Information Disclosure.....97

14.1 Duties of Each Party.....97

14.2 Information Disclosure 97

14.2.1 Ownership 97

14.2.2 Confidentiality 97

14.2.3 Publication 97

15 Procedure 98

15.1 Regulatory Approval 98

15.2 Protocol Amendment 98

15.3 Compliance with and Deviation from the Protocol 98

15.4 Policy for Paper Publication 99

15.5 Clinical Study Report 99

15.6 Contract and Financial Details 99

15.7 Insurance, Indemnity and Compensation 99

15.8 Termination of the Study 99

15.9 Management of Study Site Documents 99

16 Expected Progress 101

17 Reference 102

18 Appendices 103

Appendix 1: National Cancer Institute-Common Terminology Criteria for Adverse Events v4.03 (NCI CTCAE v4.03) 103

Appendix 2: ECOG performance status score 104

Appendix 3: Cockcroft-Gault Formula for Calculation of Creatinine Clearance 105

Appendix 4: ECG QTcB Interval Calculation (Bazett's Formula) 106

List of Tables

Table 1: Emergency Contact Information 2
Table 2: Dose delay and adjustment due to hematologic toxicities 40
Table 3: Dose delay and adjustment due to hepatic function abnormal 41
Table 4: Dose delay and adjustment due to general toxicities 42
Table 5a: PK blood sample collection time points (adult subjects) 62
Table 6b: PK blood sample collection time points (adolescent subjects) 63
Table 7: Sampling Time Point for Exploratory Biomarker 64
Table 8: Schedule of Assessments 65
Table 9: Time point response-patients with target (+/- non-target) disease 76
Table 10: Time point response-patients with non-target lesions only 76

List of Abbreviations and Definitions of Terms

Abbreviations and Terminologies

Abbreviations or Terminologies	Interpretation
AE	Adverse event
ANC	Absolute neutrophil count
ALP	Alkaline phosphatase
ALT	Alanine aminotransferase
AST	Aspartate aminotransferase
AUC	Area Under the Curve
BUN	Blood urea nitrogen
Ca	Calcium
NMPA	National Medical Products Administration
C_{max}	Maximum plasma concentration
CPK	Creatine phosphokinase
C_{ss}	Plasma concentration at steady state
CI	Confidence interval
CRF	Case Report Form
CT	Computed tomography
CTCAE	Common Terminology Criteria for Adverse Events
CBR	Clinical benefit rate
DLT	Dose-limiting toxicity
DOR	Duration of response
ECG	Electrocardiogram
ECOG	Eastern Cooperative Oncology Group
EF	Ejection fraction
eGFR	Estimated glomerular filtration rate
FDA	Food and Drug Administration
g	Gram
GCP	Good Clinical Practice
GIST	Gastrointestinal stromal tumor
Hb	Hemoglobin
HBV	Hepatitis B virus
hCG	Human chorionic gonadotropin
HCV	Hepatitis C virus
HCT	Hematocrit
HDPE	High-density polyethylene
HIAA	Hydroxyindolacetic acid
HIV	Human Immunodeficiency Virus
H_2	Histamine type 2 receptor

Abbreviations or Terminologies	Interpretation
IB	Investigator's Brochure
ICF	Informed Consent Form
IC50	Half-maximal inhibitory concentration
ICH	The International Council for Harmonisation of Technical Requirements for Pharmaceuticals for Human Use
IEC	Independent Ethics Committee
IHC	Immunohistochemistry
INR	International normalized ratio
IRB	Institutional Review Board
LD	Longest diameter
LDH	Lactate dehydrogenase
LFT	Liver function test
LLN	Lower limit of normal
mg	Milligram
m^2	Square meter
MRI	Magnetic resonance imaging
MTD	Maximum tolerated dose
Na	Sodium
NCI	National Cancer Institute
ORR	Objective Response Rate
OS	Overall survival
PD	Progressive disease
PFS	Progression-free survival
PK	Pharmacokinetics
RBC	Red Blood Cell
RP2D	Recommended phase 2 dose
SAE	Serious Adverse Event
SD	Stable disease
SDV	Source data verification
TEAE	Treatment-emergent adverse event
TKI	Tyrosine kinase inhibitor
ULN	Upper Limit of Normal
WBC	White blood cell count
WHO	World Health Organization

1 Study Background

Gastrointestinal stromal tumors (GIST) are non-epithelial mesenchymal tumors and the most common soft tissue sarcomas of the digestive tract. The majority of GISTs harbor a mutation in either KIT or PDGFRA [1]. The annual incidence of GISTs in the United States is estimated to be 3-7 per million people [2]. The annual incidence reported in Europe, South Korea and Hong Kong is even higher to be 15-20 per million people [3]. There is a lack of accurate epidemiological data in China, but there are reports that the incidence should be similar to that in Western countries (10-20 per million people) [4]. GISTs can originate from any part of the gastrointestinal tract, but the stomach (60%) and small intestine (30%) are the most common primary sites [5]. Duodenal (4%-5%) and rectal (4%) primary GISTs are less common, with a very small number of GISTs originating from the esophagus (< 1%) and colon and appendix (1%-2%) [5]. The median age of GIST patients is 60-65 years old. Common symptoms include early satiety, abdominal discomfort such as abdominal pain or abdominal distension, hemorrhage of digestive tract, anemia, and GI tract obstruction, etc. Surgery is the primary treatment option for patients with localized or potentially resectable GISTs, and approximately 60% of GIST patients can be cured by surgery. 40% of patients eventually develop metastasis following surgical treatment [3]. Imatinib is the first-line treatment for patients with metastatic GISTs, but secondary resistance is very common.

GISTs were originally thought to be refractory tumors insensitive to both conventional radiotherapy and chemotherapy, and the median survival time of metastatic GIST before imatinib is available on the market was only 12-18 months [6]. New discoveries in the biology of GISTs and the application of small-molecule tyrosine kinase inhibitors (TKIs) have revolutionized the treatment options for local and progressive GISTs. The successful application of small-molecule TKIs, represented by imatinib mesylate, in the treatment of relapsed and metastatic GISTs and GISTs with a significant risk of recurrence has greatly improved the survival of GIST patients. In 2002, the FDA approved imatinib for the first-line treatment of patients with unresectable and/or metastatic KIT-positive GISTs[7]. Although most patients with advanced GISTs benefit from imatinib treatment, some patients develop imatinib resistance. In 2006, the FDA approved sunitinib for the treatment of GIST patients with progression or intolerance after imatinib treatment [8]. In 2016, regorafinib, a multikinase inhibitor targeting KIT, PDGFR, and VEGFR, was approved by the FDA for the treatment of locally progressive, unresectable, or metastatic GIST patients who had previously received imatinib and sunitinib therapy [9]. Imatinib, sunitinib and regorafenib have been approved in China for the first-, second- and third-line treatment of patients with unresectable and/or metastatic GISTs, respectively. For patients with GISTs who have progressed after treatment with approved drug, the guidelines suggest them participating in a clinical study of new drugs [10].

A number of small-molecule targeted drugs are being developed for GIST indications in the world. Ponatinib, a third-generation Bcr-Abl inhibitor developed by Ariad Pharmaceuticals,

has shown clinical benefits and an acceptable safety profile in a completed Phase II study in GIST patients [NCT01874665] [11]. Ponatinib is currently being explored in a new Phase II study targeting GISTs, which is designed to explore the efficacy of ponatinib in patients with KIT exon mutations who failed imatinib treatment, and to explore the efficacy in patients with KIT exon 13 mutations [NCT03171389]. Ponatinib has also shown inhibitory activity against Kit, PDGFR, FGFR, and RET, and is being extensively studied in Phase II clinical trials for the treatment of solid tumors including advanced lung cancer, cholangiocarcinoma, glioblastoma, and head and neck cancer.

HQP1351 (olverembatinib, formerly known as GZD824, D824) is a highly effective, oral, third-generation multi-target tyrosine kinase inhibitor developed by Guangzhou HealthQuest Pharma Inc. with global independent intellectual property rights. It is a small-molecule TKI developed by using rational design strategy and branded medicine design, optimization, screening and evaluation technologies such as computer-aided design and X-ray drug-target binding analysis. HQP1351 could effectively bind to wild-type and various drug-resistant mutant Bcr-Abl, especially T315I mutations in preclinical trials, and is the only original Class 1 targeted antitumor drug that can be used to treat first- and second-generation TKI-resistant CML patients in China. As of February 2021, a total of 9 clinical studies of HQP1351 have been conducted. In addition, it has demonstrated a high binding effect on several other kinases, including Kit, b-RAF, DDR1, FGFR, Flt3, PDGFR, RET, Src, Tie1 and Tie2, suggesting that this compound may be effective against a variety of tumors. HQP1351 has shown an extensive inhibitory effect on the proliferation of a variety of mutant GIST cells, and could effectively inhibit primary and secondary drug-resistant *c-KIT* mutations, as well as c-KIT-independent GIST cell growth. These *in vitro* activities suggest that HQP1351 may be effective in GIST patients who have failed TKI therapy.

1.1 Pre-Clinical Pharmacodynamic Studies

HQP1351 (olverembatinib, formerly known as GZD824, D824) is highly effective in inhibiting wild-type c-KIT kinase and various mutant c-KIT kinases, which have been identified as targets for GISTs. HQP1351 showed a good inhibitory effect on L576P mutation, V559D mutation, V559D, and T670I double mutation, with an inhibition rate of > 90%. The effect on A829P, D816H and D816V was slightly weak, and the inhibition rate was 20%-40%. It is better than ponatinib, the latest third-generation Bcr-Ab1 kinase inhibitor marketed abroad, and effectively overcomes the defect that ponatinib has poor efficacy in treating P-loop mutations. In addition, in the KINOME*scan*™ kinase binding screening assay including 442 kinases, HQP1351 can efficiently bind to wild-type and drug-resistant mutant Bcr-Abl. It also showed a high inhibitory effect on some other kinases, including Flt3, PDGFR, TRK, b-RAF, DDR1, FGFR, RET, Src, Tie1, and Tie2, suggesting that this compound may be effective against a variety of tumors.

The inhibitory effect of HQP1351 on the proliferation of a total of 5 lines of KIT-mutant gastrointestinal stromal tumor cells (GIST 48B, GIST 48, GIST 430, GIST 882 and GIST T1)

was detected by CCK-8 method *in vitro*. In imatinib-sensitive GIST T1 and GIST 882 cell lines, the inhibitory effect of HQP1351 was similar to that of ponatinib and imatinib. The IC_{50} of HQP1351, ponatinib and imatinib in GIST T1 cells was 0.027±0.021 μM, 0.021±0.000 μM and 0.027±0.005 μM, respectively. The corresponding IC_{50} in GIST 882 cells was 0.036±0.007 μM, 0.021±0.016 μM, and 0.0415±0.008 μM, respectively. In imatinib-resistant GIST 48 (exon11& exon 17) and GIST 430 (exon 11 & exon 13) cell lines, HQP1351 had an IC_{50} of 0.041±0.041 μM and 0.091±0.021 μM, respectively, significantly superior to imatinib (IC_{50}=0.355±0.124 μM and 1.620±0.318 μM) and comparable to ponatinib (IC_{50}=0.027±0.031 μM and 0.134±0.057 μM). It is particularly noteworthy that GIST 48B, a derivative of GIST 48, is a *c-KIT*-mutant cell (Mu, exon 13 & exon 17) but does not express c-KIT, and is a c-KIT independent GIST cell, which is resistant to imatinib and ponatinib to varying degrees. In contrast, HQP1351 showed a good proliferation-inhibitory effect on GIST 48B cells with an IC_{50} of 0.133±0.030 μM, and its effect was 5-10 times stronger than that of ponatinib and 30 times stronger than that of imatinib.

In vitro test results showed that HQP1351 strongly inhibited the growth of a variety of human blood and solid tumor cells, including K562, Ku812 and Sup-B15 cells (with an IC_{50} of 0.2 nM, 0.13 nM and 2.5 nM, respectively). The inhibitory activity of HQP1351 against Bcr-Abl positive leukemia cells was significantly stronger than that of cytotoxic drug Taxol. The results showed that imatinib was ineffective against both breast and lung cancer (IC_{50} > 10 μM); however, HQP1351 showed a good proliferation-inhibitory effect in clinically extensively drug-resistant triple negative breast cancer (TNBC) cells and in lung cancer cells with FGFR1 amplification. The most sensitive cells were TNBC MDA-MB-231 cells (with an IC_{50} of 0.009±0.005 μM), followed by TNBC SUM-159 and H520 lung cancer cells (FGFR1 amp) (with an IC_{50} of 0.081±0.025 μM and 0.036±0.006 μM, respectively). Compared with ponatinib, the effect of HQP1351 was 1-5 times stronger in cells other than HCC1428, H520 and BT-20 cells, including MCF7, BT-474, HCC1954, SUM-159, MDA-MB-231 and HCC827.

The antitumor mechanism of HQP1351 has been preliminarily explored. Studies have confirmed the inhibitory effect of HQP1351 on KIT signaling pathways at the cellular level. In imatinib-sensitive GIST T1 and GIST882 and imatinib-resistant GIST 430 cells, HQP1351 significantly inhibited the phosphorylation levels of p-c-KIT, p-AKT and p-ERK1/2, and was much superior to imatinib and slightly superior to ponatinib in terms of inhibitory effect at the same concentration. Imatinib-resistant GIST 48 cells were c-KIT exon 11 and 17 mutants. HQP-1351 inhibited the expression of c-KIT and its downstream STAT3 phosphorylated protein, and showed an inhibitory effect superior to that of ponatinib.

Multiple *in vivo* pharmacodynamic tests showed that HQP1351 monotherapy showed a significant dose-time dependent growth-inhibitory effect in a variety of animal models of GIST human gastrointestinal stromal tumor with different *c-KIT* mutations, including imatinib-sensitive GIST T1 and GIST 882, imatinib-resistant GIST48 and GIST430, and MDA-MB-

231 triple negative breast cancer xenografts. The minimal effective dose of HQP1351 given every other days (qod) to GIST models was 20 mg/kg, and the T/C (%) after the end of dosing was 30.8%-69.8%. HQP1351 when given every other day for 3 weeks (qod × 3wks) had a better tumor growth-inhibitory effect than the dosing regimen (qod × 2wks). HQP1351 when given every other day (qod), every third day (q3d) or once a week (qw) for 3 weeks, could effectively inhibit tumor growth, with statistically significant differences ($P<0.05$). In the MDA-MB-231 model, the minimal effective dose was 20 mg/kg, and the T/C (%) was 34.0% ($P<0.01$). In addition, co-administration with docetaxel could enhance the efficacy of docetaxel for TNBC. HQP1351 showed a better antitumor effect than ponatinib in GIST T1, GIST 882 and GIST 48 models under the same dose and dosing conditions. HQP1351 showed no significant inhibition of weight gain or drug discontinuation due to health conditions, except for about 10% weight loss in some tests under the 60 mg/kg qod 3 wks dosing regimen. The results showed that HQP1351 monotherapy given every other day (QOD) had a good growth-inhibitory effect in various *c-KIT*-mutant GIST and triple negative breast cancer xenograft models.

In vivo pharmacodynamic (PD-PK) tests confirmed that following oral administration of HQP1351 at dose levels of 20, 40 and 60 mg/kg QOD for 21 consecutive days, a dose-related inhibitory effect on phosphorylation of KIT, SRC and its downstream associated proteins AKT and STAT3 in GIST 48 tumors was detected 4 hours after the last dose. At the same dose of 60 mg/kg, its inhibitory effect was comparable to that of ponatinib. Accordingly, the concentration of HQP1351 in tumor tissues 4 hours after the last dose of 20, 40 and 60 mg/kg was 1,379, 864 and 1,774 ng/ml, respectively, while the concentration of ponatinib (60 mg/kg) in tumor tissues was higher (4,325 ng/mL), about twice that of HQP1351. The 4 h plasma concentration of HQP1351 at dose levels of 20, 40 and 60 mg/kg was 122, 112 and 203 ng/ml, respectively, and that of ponatinib was 359 ng/ml, about twice that of HQP1351. These results indicated that although the concentration of HQP1351 in plasma and tumor tissues was lower than that of ponatinib, its inhibitory effect on KIT, SRC, AKT and STAT3 signaling pathways was not inferior to that of ponatinib. In addition, the concentration of HQP1351 in plasma and tumor tissues did not increase dose-dependently across different dose groups, indicating that concentration reached a saturation state to some extent.

In a separate PK/PD test conducted in the GIST T1 model, following administration of HQP1351 at dose levels of 20, 40 and 60 mg/kg once a day for 3 consecutive days, an inhibitory effect on phosphorylation of KIT and SRC in tumor tissues could be detected in each dose group 4 hours after the last dose, and the effect lasted until 48 hours. HQP1351 showed the strongest inhibitory effect at the dose of 60 mg/kg, which was comparable to that of ponatinib at the same dose level. Accordingly, in tumor tissues, the drug could be detected 4 hours post-dose, and even 48 hours post-dose. Its concentrations in tumor tissues were dose-dependent, and the exposures at 48 hours post-dose at 20, 40, and 60 mg/kg was 8,950, 12,319 and 20,984 hr*ng/mL, respectively. The exposure of ponatinib 60 mg/kg in tumor tissues was higher

(44,787 hr*ng/mL), about twice that of HQP1351. Similarly, in plasma, the drug was still detectable 48 hours after HQP1351 administration. The $AUC_{0-\infty}$ at 20, 40 and 60 mg/kg was 1,122, 1,739 and 3,406 h*ng/mL, respectively, showing dose correlation; the C_{max} was 249, 216 and 213 ng/mL, respectively, without significant dose correlation. The concentration of ponatinib was below the detection limit after 48 hours, the $AUC_{0-\infty}$ was 2,828 h*ng/mL, and the C_{max} was 411 ng/mL, which was about twice that of HQP1351. However, its AUC_{last} was comparable to that of HQP1351. These results suggested that HQP1351 had similar inhibitory effects on phosphorylation of c-KIT, AKT, STAT3 and SRC, although the exposure of HQP1351 in tumor tissues was lower than that of ponatinib at the same dose level. In summary, compared with the ponatinib marketed abroad, HQP1351 effectively overcomes the ineffectiveness of the marketed drug against KIT kinase A-Loop-mutant resistant GIST, and has shown a good growth-inhibitory effect *in vivo* against *KIT*-mutant resistant GIST and KIT-independent GIST, as well as other solid tumors, such as breast cancer.

1.2 Safety Pharmacology Studies

Study results showed that HQP1351 had no inhibitory activity on hERG potassium channels. The safe dose of HQP1351 granules by single intragastric administration on the cardiovascular and respiratory function of Beagle dogs and the safe dose on the function of the central nervous system in SD rats were both equal to or more than 1.5 mg/kg.

1.3 Pre-clinical Pharmacokinetic Studies and Metabolism Studies

The PK of HQP1351, including absorption, tissue distribution, metabolism, and excretion characteristics, were investigated in rats and Beagle dogs.

After intragastric administration of HQP1351 to rats under fasting condition, the mean time to peak plasma concentration (T_{max}) was 6-8 h, and plasma elimination half-life ($t_{1/2}$) was 3-6 h. The absolute bioavailability of HQP1351 in rats was 14.00%, 40.54%, and 36.02% after intragastric administration of 0.5, 1.0 and 1.5 mg/kg, respectively. Following intragastric administration of HQP1351 to Beagle dogs under fasting conditions, the mean T_{max} was 1.67 - 2.83 h, and $t_{1/2}$ was 6.62 - 9.72 h. The absolute bioavailability of HQP1351 in Beagle dogs was 5.11%, 12.89% and 14.45% after intragastric administration of 0.75, 1.5 and 2.25 mg/kg, respectively. Following intragastric administration of HQP1351 to dogs for 7 consecutive days, there was no obvious accumulation of plasma exposure to HQP1351 in rats and dogs.

After 24 hours following oral administration of HQP1351 granules to SD rats, the $AUC_{(0-24h)}/C_{max}$ ratio in each dose group (0.5, 1 and 2 mg/kg/day) was 1:3.75:13.16 and 1:3.61:15.50, respectively; after continuous administration for 28 days, the $AUC_{(0-24h)}/C_{max}$ ratio in each dose group (0.5, 1 and 2 mg/kg/day) was 1:2.34:7.72 and 1:1.63:5.03, respectively. The mean in vivo C_{max} and $AUC_{(0-24)}$ generally increased with dose (0.5 -2 mg/kg/day). After administration for 28 consecutive days, $AUC_{(0-24h)D28}$/ $AUC_{(0-24h)D1}$ of HQP1351 in 0.5, 1, and 2 mg/kg/day groups was 3.05, 1.90, and 1.79, respectively. In the 0.5 mg/kg/day dose group, the corresponding AUC_{0-24h} and C_{max} on Day 28 were 40.83 ng•h/mL and 4.8 ng/mL in male

animals, and 51.22 ng•h/mL and 5.26 ng/mL in female animals, respectively. In the 1 mg/kg/day dose group, the corresponding $AUC_{0\text{-}24h}$ and C_{max} on Day 28 were 89.58 ng•h/mL and 7.2 ng/mL in male animals, and 125.35 ng•h/mL and 9.36 ng/mL in female animals, respectively. In the 2 mg/kg/day dose group, the corresponding $AUC_{0\text{-}24h}$ and C_{max} on Day 28 were 409.43 ng•h/mL and 28.67 ng/mL in male animals, and 300.93 ng•h/mL and 25.22 ng/mL in female animals, respectively. *In vivo* exposure increased to a certain extent in each dose group after multiple doses. After the first dose and administration for 28 consecutive days, $AUC_{(0\text{ - }24h)}$ of female animals was slightly higher than that of male animals except $AUC_{(0\text{ - }24h)}$ of male animals was higher than that of female animals in the high-dose group after the last dose.

HQP1351 was administered to Beagle dogs at 0.75 mg/kg/day and 1.5 mg/kg/day once daily for 28 consecutive days and at 3 mg/kg/day once daily for 21 consecutive days, respectively. In the dose range of 0.75 - 3 mg/kg/day, $AUC_{(0\text{ - }24h)}$ and C_{max} of HQP1351 in Beagle dogs increased with dose. After the first dose, the $AUC_{(0\text{-}24h)}/C_{max}$ ratios of plasma HQP1351 in each dose group (0.75, 1.5, and 3 mg/kg/day) were 1:3.90:9.93 and 1:4.93:11.19 in female Beagle dogs, and 1:5.43:10.54 and 1:6.07:14.92 in male Beagle dogs, respectively. After 28 days of continuous administration, the $AUC_{(0\text{-}24h)\ D28}/AUC_{(0\text{-}24h)\ D1}$ in the 0.75 and 1.5 mg/kg/day dose groups was 2.39 and 0.65 in female Beagle dogs, and 0.86 and 0.84 in male Beagle dogs, respectively. After 21 days of continuous administration, the $AUC_{(0\text{-}24h)\ D21}/AUC_{(0\text{-}24h)\ D1}$ in the 3 mg/kg/day dose group was 0.63, and the corresponding $AUC_{(0\text{-}24h)}$ and C_{max} were 70.68 h·ng/mL and 10.28 ng/mL in female Beagle dogs, respectively; the $AUC_{(0\text{-}24h)\ D21}/AUC_{(0\text{-}24h)\ D1}$ in the 3 mg/kg/day group was 1.20, and the corresponding $AUC_{(0\text{-}24h)}$ and C_{max} were 262.07 h·ng/mL and 26.85 ng/mL in male Beagle dogs, respectively. There was no significant accumulation or decrease in systemic exposure after multiple doses of the test article. On Day 28, the corresponding $AUC_{(0\text{-}24h)}$ and C_{max} at 0.75 mg/kg/day dose group were 23.81 h·ng/mL and 3.22 ng/mL in female animals, and 12.58 h·ng/mL and 1.83 ng/mL in male animals, respectively. On Day 28, the corresponding $AUC_{(0\text{-}24h)}$ and C_{max} at 1.5 mg/kg/day dose group were 31.75 h·ng/mL and 3.57 ng/mL in female animals, and 74.14 h·ng/mL and 9.67 ng/mL in male animals, respectively. After administration at 1.5 mg/kg for 28 consecutive days, exposure to HQP1351 in male Beagle dogs was significantly higher than that in female animals, with statistically significant difference ($P \leq 0.05$). There was no significant sex difference in drug exposure in Beagle dogs across other dose groups ($P > 0.05$).

No significant difference was observed in binding rate of HQP1351 to plasma protein of human, rats and dogs at different concentrations or among species. Following oral administration of 1.0 mg/kg HQP1351 to rats, HQP1351 was widely distributed in the tissues, and the main distribution tissues were lung, liver, and kidney, and the concentrations of HQP1351 in lung, liver and kidney was much higher than that in plasma.

HQP1351 had an inhibitory effect on CYP2C9 and CYP2C19 in the test concentration range (0 - 50 μM) and no significant inhibitory effect on CYP1A2, CYP3A4, and CYP2D6.

HQP1351 had a potential to induce mRNA expression of CYP1A2 and CYP3A4 at concentrations of 0.563 and 5.63 μM, and had a potential to induce mRNA expression of CYP2B6 at all test concentrations. Human recombinant enzyme assays further demonstrated that CYP3A4 and CYP2C9 are the major metabolic enzymes that catalyze the oxidative metabolism of HQP1351.

HQP1351 was relatively stable in liver microsomes of human, monkey, dog, and rat. Less than 20% of unchanged drug was oxidized NADPH-dependently, with similar major metabolic pathways, including N-demethylation, N-oxidation of methylpiperazine ring and monohydroxylation of the right part of amide bond. After incubation of HQP1351 for 60 min in mouse liver microsomes, there was less than 10% of unchanged drug, and N-demethylation metabolite was mainly detected. 12 types of metabolites were detected in rats after intragastric administration of HQP1351. Unchanged drug and metabolites were mainly excreted into feces via biliary excretion, the N-demethylation was the major metabolic pathway. Within 0 to 72 h after intragastric administration of HQP1351 in male rats, the total cumulative excretion of unchanged drug and metabolites in fecal and urine samples accounted for 18.6% and 0.150% of the administered dose, respectively, amounting to 18.7%, among which the unchanged drug accounted for 13.2%; The total cumulative excretion of the unchanged drug and its metabolites in bile of bile-duct-cannulated rats from 0 to 48 h accounted for 0.207% of the administered dose. The total cumulative excretion of unchanged drug and metabolites in fecal and urine samples of female rats accounted for 3.40% and 0.180% of the administered dose, respectively, amounting to 3.58%, among which the unchanged drug accounted for 2.24%; The total cumulative excretion of the unchanged drug and its metabolites in bile of bile-duct-cannulated rats from 0 to 48 h accounted for 0.271% of the administered dose. The difference between female and male rats was primarily due to much more excretion in feces of male rats than that of female rats, about 4.51 times that of female rats.

1.4 Preclinical Toxicology Studies

The results of acute toxicity test showed that: the maximum tolerated dose (MTD) of HQP1351 by single intragastric administration in SD rats was 15 mg/ kg, and the lethal dose was 45 mg/ kg; the MTD of HQP1351 by single oral administration in Beagle dogs was 20 mg/kg and the lethal dose was 60 mg/ kg.

According to long-term toxicity test, in 28-day repeat-dose toxicity study (28-day recovery period) in rats, early animal death was observed following administration of HQP1351 granules at 2 mg/kg/day on a daily basis for 28 consecutive days. Total protein, albumin and globulin of female animals decreased and fibrinogen of male and female animals increased. The main target organs for toxicity were gastrointestinal tract, thymus, spleen, lymph nodes, Peyer's patches, bone marrow, liver, thyroid gland and parathyroid glands. After 4-week drug withdrawal for recovery, surviving animals in this group completely recovered to normal. In the 0.5 mg/kg/day and 1 mg/kg/day groups, neutrophils and monocytes increased and lymphocytes decreased; in the ≥ 0.5 mg/kg/day group, activated partial thromboplastin time

shortened was observed in male animals; in the ≥ 1 mg/kg/day group, prothrombin time shortened was observed in female animals; after 4-week drug withdrawal, surviving animals completely recovered to normal. The no observed adverse effect level (NOAEL) was 1.0 mg/kg/day.

In a 28-day repeat-dose toxicity study in Beagle dogs (28-day recovery period), red blood cells, hemoglobin, hematocrit, platelets, white blood cells, neutrophils, and fibrinogen increased in 3 mg/kg/day dose group. Total protein and globulin decreased, A/ G, fibrinogen, creatine kinase increased in the 1.5 mg/kg/day dose group. In addition, lymphocytes decreased was observed in males and white blood cell and neutrophils increased were observed in females. In 1.5 mg/kg/day dose group, white blood cells and lymphocytes increased were observed in male animals and globulins decreased and A/G increased were observed in female animals. After the recovery period, all of the above changes recovered to normal. Intragastric HQP1351 3 mg/kg/day on a daily basis for 21 consecutive days resulted in animal death in an early period. The main target organs for toxicity were stomach, intestine, thymus, spleen, lymph nodes and Peyer's patches, sternal bone marrow, lip and tongue mucosa, and toxicities were reversible to a certain extent. After 4-week drug withdrawal, all of the above symptoms of surviving animals in this group recovered to normal. The NOAEL was 1.5 mg/kg/day.

Genotoxicity test results showed that HQP1351 had no teratogenicity on chromosomes of Chinese hamster lung fibroblasts, no mutagenicity to salmonella typhimurium and no genotoxicity on bone marrow cells.

1.5 HQP1351 Clinical Overview

As of February 2021, HQP1351 has undergone a total of 9 clinical studies, including 3 Phase I studies (SJ-0002, SJ-0003 and HQP1351CU101), 3 clinical pharmacology studies [HQP1351LC104, HQP1351XC105 and HQP1351XC106 (in healthy volunteers)] and 3 Phase II pivotal studies (HQP1351CC201, HQP1351CC202 and HQP1351CC203). All studies were conducted in China except for HQP1351CU101 and HQP1351XC106 which were conducted in the United States. The Phase I clinical study of HQP1351 for GIST (SJ-0003) is ongoing in Chinese mainland. A total of 36 GIST subjects were enrolled as of January 31, 2021, and treated with HQP1351 orally QOD in 4 dose groups: 3 in the 20 mg dose group, 11 in the 30 mg dose group, 11 in the 40 mg dose group, and 11 in the 50 mg dose group, in 28-day cycles. All subjects were TKI-resistant GIST subjects who had previously been heavily treated. The Phase I study is currently actively enrolling subjects. Of the 36 GIST subjects, 28 have completed at least one efficacy assessment. The results are as follows: 10 subjects had stable disease (SD) and 18 subjects had progressive disease (PD).

1.5.1 Clinical pharmacokinetics

In the Phase I CML study of HQP1351 (SJ-0002), the PK of HQP1351 were assessed in a total of 65 CML patients receiving HQP1351 at dose levels of 1-60 mg every other day under fed conditions. Within the dose range of 1-60 mg, the median T_{max} of HQP1351 was 4-8 h after a

single oral administration, and the mean $T_{1/2}$ was 17.5-36.5 h. Given once every other day, HQP1351 reached steady state on Day 7 of administration. The average accumulation ratio of AUC_{0-48h} and C_{max} was 1.15-1.98 and 0.91-1.66, respectively, indicating moderate accumulation of HQP1351 after continuous multiple administrations every other day.

AUC and C_{max} of HQP1351 after a single dose (1-60 mg dose range) and after multiple doses (1-50 mg dose range) increased in an approximately dose-proportional manner.

1.5.2 Clinical safety

Preliminary results of Phase I and II clinical trials show that HQP1351 has a favorable safety profile at the dose level of 50 mg QOD. The plasma exposure (AUC and C_{max}) of HQP1351 after a single dose (1-60 mg dose range) and after multiple doses (1-50 mg dose range) increased in an approximately dose-proportional manner.

Safety data were obtained from a total of 269 CML patients in 5 CML studies with safety and efficacy as primary or secondary endpoints. The data cutoff date for SJ-0002, CC201, and CC202 was 12 months after enrollment of the last patient; the data cutoff dates for CC203 and CU101 were May 25, 2021, and January 31, 2021, respectively. The mean treatment period of study drug was 13.1 (range: 0.2; 38.9) months. Approximately 99% of subjects experienced at least one treatment-emergent adverse event (TEAE), and most TEAEs were mild or moderate and could be resolved with or without interruption, dose reduction, or discontinuation of the study drug. The most common TEAEs (≥ 20%) included hematological abnormalities (reduced platelet count, white blood cell count decreased/neutrophil count decreased, and anemia), skin pigmentation, liver function test abnormal (ALT increased, AST increased, GGT increased, or blood bilirubin increased), metabolic disorder (hypertriglyceridemia, hypocalcemia), and others (pyrexia, proteinuria and blood creatine phosphokinase increased). 10% of patients withdrew from the study due to AEs. 26% of patients experienced SAEs, but no deaths due to AEs were reported. Based on the safety profile of HQP1351 in CML, reduced platelet count and anemia were the most frequently reported SAEs related to the study drug, and were identified as important risks of HQP1351 in the treatment of CML patients. Based on available data, HQP1351 is well tolerated in CML patients and has a positive benefit/risk ratio for CML patients who are resistant or intolerant to multiple TKIs. Data were obtained from 36 GIST subjects in the SJ-0003 study, including 3, 11, 11, and 11 subjects enrolled in 4 designated dose cohorts (20 mg QOD, 30 mg QOD, 40 mg QOD, and 50 mg QOD), and the safety analysis was based on a combination of the 4 dose groups. As of January 31, 2021, the mean duration of treatment for GIST patients was 2.5 (range 0.1-13.1 months) months. All subjects (100%) experienced at least one TEAE, and most TEAEs were mild or moderate and could be resolved or recovered with or without interruption, dose reduction, or discontinuation of the study drug. Among them, 5 (14%) subjects discontinued treatment due to AEs. The most common hematological AEs (≥ 20% TEAEs) in GIST subjects included white blood cell count increased (63.9%), neutrophil count increased (52.8%), and anemia (41.7%). The most common non-hematological AEs (≥ 20% TEAEs) included constipation (44.4%), asthma (38.9%), aspartate

aminotransferase increased (33.3%), hyperuricemia (33.3%), hypoproteinemia (27.8%), alanine aminotransferase increased (25.0%), serum creatinine increased (25.0%), abdominal pain (22.2%), and C-reactive protein increased (22.2%). Ten (28%) subjects experienced at least one serious adverse event (SAE), and GI tract obstruction, as the most commonly reported SAE in GIST patients, was due to PD of GIST and probably unrelated to HQP1351. Four (11%) subjects experienced fatal AEs, 1 due to renal failure, 1 due to intestinal perforation and septic shock arising from intestinal obstruction, 1 due to death unexplained (presumed by the investigator to be in the terminal stage of the disease), and 1 due to PD, all of which were assessed by the investigator as unlikely related to the drug. Available clinical data suggest that HQP1351 when dosed at 30 mg, 40 mg, and 50 mg is well tolerated in patients with CML and GIST.

Based on the safety data from 9 clinical studies that have been conducted with HQP1351 and the safety and efficacy data of 36 patients already enrolled in this study, 40 mg QOD is selected as the dosage for adult subjects after discussion between the investigator and the sponsor, and no randomization at 30 mg, 40 mg or 50 mg QOD will be performed.

1.6 Risk-benefit and Ethical Assessment

Available clinical data suggest that HQP1351 is well tolerated in patients with GIST at dose levels of 30 mg, 40 mg, and 50 mg and is effective for the treatment of advanced GIST, with a positive benefit/risk ratio.

As of August 2021, 6 wild-type GIST subjects were enrolled, all of whom had previously been treated with ≥ 1 targeted drug (3 had previously been treated with 3 drugs, 2 with 2 drugs, and 1 with a targeted drug). The preliminary efficacy assessment was PR in 1 subject (archived in Cycle 9, which remained PR when followed up to Cycle 13), SD in 3 subjects (followed up for more than 11 cycles, up to 31 cycles), SD in 1 subject when followed up to Cycle 3, and PD in 1 subject on Day 52 of medication, proving certain efficacy for wild-type GIST. Pediatric children with metastatic GIST are very rare, and more than 85% of them are wild-type with primary resistance to imatinib and no standard treatment available, facing the predicament of no medicine available for treatment. Given that the National Medical Products Administration encourages the inclusion of adolescents in tumor clinical trials as much as possible, after the preliminary adult data (currently there are safety data and preliminary PK data and efficacy data from more than 300 subjects) are available, it is planned to include adolescent wild-type GIST subjects over 12 years old without standard treatment in clinical trials for adult so as to enable these adolescent GIST patients to have the opportunity to enter clinical trials, obtain their clinical data as soon as possible, and solve the problem of no medicine available for these extremely rare patients.

More information about the known and expected benefits and risks of HQP1351 can be found in the relevant section of the Investigator's Brochure (IB) for HQP1351. The sponsor, CRA, and investigators will conduct the study in accordance with the study protocol, the Good

Clinical Practice (GCP), the International Council for Harmonization of Technical Requirements for Pharmaceuticals for Human Use (ICH) guidelines, and applicable regulatory requirements.

2 Study Objectives

2.1 Primary Objective

To assess the safety and tolerability of HQP1351 in different dose groups in the treatment of patients with advanced GIST or other solid tumors, and determine the RP2D.

2.2 Secondary Objectives

- To preliminarily assess the efficacy of HQP1351 in patients with advanced GIST or other solid tumors.
- To assess the PK of HQP1351.

2.3 Exploratory Objective

- To explore biomarkers related to the efficacy of HQP1351 monotherapy.

3 Trial Population

3.1 Inclusion Criteria

Subjects will be eligible for the study if they meet each of the following criteria:

1. Aged ≥ 12 years, male or female.
2. Advanced and/or metastatic GIST or other solid tumors, confirmed by histology and/or cytology. Among them, GIST patients must have primary resistance to imatinib (tumor progression within 6 months after first-line use of imatinib, or immunohistochemical presence of SDHB expression deletion or NF1 mutation) or have failed treatment with imatinib or imatinib plus at least one other TKI (following initial imatinib or other TKI treatment for more than 6 months, the subject has tumor progression again after achieving a response or stable disease).
3. ECOG score ≤ 2.
4. Life expectancy of ≥ 3 months.
5. Hematologic function as outlined below:
   - Absolute neutrophil count (ANC) ≥ 1.5 $\times 10^9$/L (ANC achieved by colony-stimulating factors will not be used);
   - Platelet count ≥ 100 × 10^9/L (baseline platelet count achieved by blood transfusion or thrombopoietic growth factor will not be used);
   - Hemoglobin ≥ 90 g/L

     Note: If hemoglobin is stable for 14 days after transfusion (a decrease of no more than 10 g/L), transfusion is allowed. The use of erythropoiesis-stimulating agents (ESAs) is allowed;
   - Serum albumin ≥ 30.0 g/L;
   - Serum lipase ≤ 1.5 × upper limit of normal (ULN);
   - Serum amylase ≤ 1.5 × ULN.
6. Hepatic and renal function:
   - Serum creatinine ≤ 1.5 × ULN; or serum creatinine > 1.5×ULN, and creatinine clearance ≥ 50 mL/min;
   - Serum total bilirubin ≤ 1.5 × ULN;
   - Aspartate aminotransferase (AST) and alanine aminotransferase (ALT) ≤ 2.5 × ULN (≤ 5 × ULN in the presence of liver metastasis).
7. Cardiac function parameters:
   - Troponin (I or T) ≤ ULN;

- Ejection fraction (EF) > 40%;
- QTc interval on electrocardiogram (corrected by Bazett's formula): male ≤ 450 ms and female ≤ 470 ms.

8. Women of childbearing potential should have a negative serum pregnancy test within 7 days prior to the first dose of the study drug.
9. Men and women of childbearing potential (postmenopausal women must be amenorrheic for at least 12 months to be considered of non-childbearing potential) and their partners are willing to take protocol-specified effective contraceptive measures from signing the informed consent from to at least 30 days after the last dose of the investigational product.
10. Able to understand and voluntarily sign the written ICF before any study-specific procedures.
11. Subjects must be willing and able to complete study procedures and follow-up examinations.

3.2 Exclusion Criteria

Subjects who meet any of the following criteria are not eligible for this study:

1. Subjects who have received antitumor cytotoxic chemotherapy, biologic drug therapy (such as monoclonal antibodies), immunotherapy (such as interferon), or radiotherapy within 28 days prior to the first dose or less than 5 times the half-life.
2. Subjects who have received tyrosine kinase inhibitor (TKI) treatment within 14 days prior to the first dose.
3. Subjects who have received other study drugs within 14 days prior to the first dose.
4. Subjects who have not recovered from adverse events (except alopecia) (> NCI-CTCAE v4.03 grade 1) due to prior treatment.
5. Malabsorption syndrome or other diseases affecting oral drug absorption.
6. Clinically significant, uncontrolled, or active cardiovascular diseases, including but not limited to history of myocardial infarction; history of unstable angina; history of congestive heart failure or left ventricular ejection fraction (LVEF) below the lower limit of normal within 6 months; history of atrial arrhythmia judged by the investigator to be of important clinical significance; history of ventricular arrhythmia, etc.
7. Hypertension inadequately controlled by antihypertensive drugs (systolic pressure >150 mmHg or diastolic pressure >90 mmHg).
8. Subjects who need to take drugs that has a known potential to cause ECG QT prolonged.
9. Pulmonary arterial systolic pressure >35 mmHg as indicated by Echocardiography.
10. Subjects who have serious cardiovascular diseases during prior TKI use.

11. Hypertriglyceridemia that is difficult to control.
12. Major surgery, open biopsy (except intravenous catheter placement or bone marrow biopsy), or major traumatic injury within 14 days prior to the administration of study drug.
13. Arterial thrombosis or embolic events such as cerebrovascular accidents (including transient ischemic attacks), or pulmonary embolism or venous thrombosis events within 6 months prior to the administration of study drug, such as deep vein thrombosis within 3 months prior to the administration of study drug.
14. Subjects who require concomitant immunosuppressive therapy, other than corticosteroids prescribed for a short course of therapy.
15. Subjects with brain metastases.
16. Patients with other primary malignant neoplasm within recent 3 years (patients who are cured for ≥ 5 years, or with completely resected non-melanoma skin cancer, or successfully treated carcinoma in situ, or prostatic carcinoma under control will be included).
17. Active symptomatic viral infections (including known HIV infections, viral hepatitis (B or C, etc.), syphilis, etc.).
18. Subjects who are known to be allergic to any components of the investigational drug or its analogues.
19. Female subjects who are pregnant or breastfeeding, or who expect to become pregnant during the study period.
20. Subjects who have any symptom or disease that, according to the opinions of the investigator or sponsor, would comprise their safety or interfere with the safety assessment of the investigational drug.

3.3 Discontinuation of Study Treatment/Withdrawal from the Study

All subjects may discontinue the study treatment or withdraw from study at any time. If a subject terminates study treatment or withdraws from the study, the investigator should record the causes for study treatment discontinuation or study withdrawal in the eCRF, and complete the visit specified by the protocol.

3.3.1 Termination of study treatment

The causes for subjects' discontinuation of study treatment may include:

- AE;
- Lost to follow-up;
- Death;
- Subjects with poor compliance are not suitable for continuing the study, as judged by the

investigator.

- The subject withdraws the ICF;
- New anticancer treatment is started;
- Termination of the study by the sponsor;
- PD;
- Others (e.g., the subject can no longer benefit from the study treatment when continuing the study at the discretion of investigator).

For subjects terminating study treatment, see Sections 7.6 and 7.7 for treatment termination and follow-up procedures. For subjects who terminate study treatment due to AEs, investigators will follow up them until the AEs are resolved or stable.

3.3.2 Withdrawal from the Study

All follow-up procedures will be stopped if a subject withdraws from the study. Reasons for withdrawal from the study may include:

- Death;
- Lost to follow-up;
- The subject withdraws the ICF for participation in the study (including follow-up period);
- The sponsor terminates the study.

3.4 Patient Re-screening Policy

For subjects who do not meet the protocol requirements during their initial screening, once their informed consent is obtained, re-screening is allowed (only 1 re-screening can be performed). When subjects are re-screened at the same site, the subject screening number must be the same as the original. Reasons for subjects not being enrolled after initial screening will be recorded in the medical record. For re-screened subjects at the same site, the data from the initial screening will not be recorded in the EDC.

3.5 Site Closure

The sponsor, Ethics Committee, drug clinical trial institution and investigator can suspend or terminate the clinical trial according to the risk/benefit assessment of clinical trial, inform mutually and describe the reasons.

If the sponsor suspends or terminates the study prematurely, the sponsor will promptly inform all participating investigators, drug clinical trial institutions, Ethics Committees and provincial drug regulatory authorities, National Medical Products Administration, and clarify the reasons.

4 Clinical Study Plan

4.1 Study Design

This is an open-label, multicenter, Phase I clinical trial which is designed to determine the safety and tolerability of HQP1351 in patients with advanced GIST and other solid tumors, to determine RP2D, and to assess the preliminary efficacy of HQP1351 in patients with advanced GIST and other solid tumors. Patients with histologically and/or cytologically proved, advanced and/or metastatic GIST or other solid tumors will be enrolled. Among them, GIST patients must have primary resistance to imatinib or have failed treatment with imatinib plus at least one other TKI. In some rare cases, GIST patients who have failed imatinib treatment alone may also be included in this study if considered to be eligible for enrollment after careful judgment by the investigator.

As of February 2021, HQP1351 has undergone a total of 9 clinical studies, and preliminary results from the Phase I and Phase II clinical trials that have been conducted show a favorable safety profile in the dose range of 50 mg QOD. HQP1351 (olverembatinib tablets) has been granted conditional marketing authorization from the National Medical Products Administration on November 24, 2021 (approval number: NMPA H20210048). It is indicated for the treatment of "adult patients with tyrosine kinase inhibitor (TKI)-resistant chronic phase chronic myeloid leukemia (CML-CP) or accelerated-phase CML (CML-AP) harboring the T315I mutation as confirmed by a validated diagnostic test", and the recommended dose is 40 mg QOD.

The study is initially designed as a Phase I study with standard 3+3 dose escalation. Based on the study results of HQP1351 SJ-0002 in CML patients and the consensus reached at the SJ-0003 kick-off meeting with the investigator, this study will explore the safety in the 30 mg, 40 mg, and 50 mg dose groups in parallel, and 10 subjects will be enrolled by means of randomization at each dose level using the IWRS. After preliminary adult data are available, adolescent subjects with advanced and/or metastatic wild-type GIST will be included. The dosage for adolescents is based on the adult dose and the selected dose will be adjusted according to their body weight. If the subject's body weight is ≥ 40 kg, the dose of 40 mg QOD will be given based on the adult dose. If the subject's body weight is ≥ 30 kg and < 40 kg, the dose of 30 mg QOD will be given. If the subject's body weight is ≥ 20 kg and < 30 kg, 20 mg QOD will be given. Based on the safety data from 9 clinical studies that have been conducted with HQP1351 and the safety and efficacy data of 36 patients already enrolled in this study, 40 mg QOD is selected as the dosage for non-randomized adult subjects after discussion between the investigator and the sponsor.

HQP1351 tablets will be administered orally to eligible subjects once every 2 days in a 28-day cycle. Subjects will receive treatment until PD, intolerable toxicity, death, or termination of treatment for any other reason. If a subject is assessed to have PD according to the RECIST v1.1 criteria, but the investigator determines that the subject will benefit from continuing the

study, then the subject may continue to take HQP1351 and undergo safety follow-up yet no more efficacy follow-up after PD is documented. During medication, subjects will not receive other antitumor study drugs, marketed drugs, or treatment methods other than HQP1351. Symptomatic (including those directed at controlling symptoms resulting from the patient’s malignancy) and supportive treatments are allowed.

In the standard 3+3 dose escalation part of this study, blood samples from subjects in Cycle 1 will be collected for the PK analysis of HQP1351. In the randomized part, 1-3 study sites will be selected to collect the blood samples of the first 3 enrolled subjects in each dose group in Cycle 1 for the PK analysis of HQP1351. If for any reason complete PK samples are not obtained from the first 3 subjects, they will be replaced by subjects subsequently enrolled in the dose group until complete PK samples are obtained from 3 subjects. PK blood samples will be collected from all non-randomized adult subjects receiving a fixed dose of 40 mg QOD for PK analysis. PK blood samples will be collected from all adolescent subjects for PK analysis.

Biological samples will be collected from all subjects for relevant biomarker testing and assessment.

All subjects will undergo efficacy assessment every 8 weeks during treatment, along with assessment of safety information such as AEs and laboratory tests.

Subjects enrolled in the Phase I study with standard 3+3 dose escalation who have received at least one cycle of HQP1351 after this protocol amendment takes effect, and have not experienced related or possibly related Grade ≥ 2 toxicity nor have tumor progression, may receive a higher dose of HQP1351 upon their own informed consent after discussion between the investigator and the sponsor. The subject will continue to be followed up according to the protocol, but will not be included in the number of randomized subjects.

4.2 Intolerable Toxicity

Intolerable toxicity is defined as:

- An AE that may affect the subject's ability to continue study-specific procedures or is not in the subject's best interest, at the discretion of the investigator or the Sponsor.
- Toxicity requiring more than twice dose reductions of HQP1351.
- HQP1351 allergy and serious allergic reactions (e.g., hypotension, bronchospasm, and/or rash generalised/erythema).

Subjects who experience intolerable toxicity will permanently discontinue the study unless it is in the subject's best interest to continue the study after the investigator has evaluated the subject’s risks and benefits and discussed with the Sponsor.

4.3 Dose Delay and/or Dose Reduction

4.3.1 Dose interruption

Treatment may be interrupted for up to 2 weeks (14 days) to allow study drug-related toxicity to recover to baseline or Grade ≤ 1. For non-treatment-related or unexpected toxicity, whether to discontinue treatment should be decided after discussion between the investigator and the sponsor. If HQP1351 is discontinued for more than 2 weeks (14 days), the subject will be withdrawn from the study. The subject can stay in the study to receive low-dose HQP1351 treatment (determined upon discussion by the principal investigator and the sponsor) if it is considered that the subject can benefit from continuous study treatment.

4.3.2 Dose adjustment

AEs that occur during this study will be graded according to National Cancer Institute-Common Terminology Criteria for Adverse Events (NCI-CTCAE) (v4.03). According to the correlation of AEs with the study drug and grade, the investigator will take different actions with the study drug, including maintaining the original treatment regimen, withholding dosing, reducing the dose, and terminating the treatment. Up to twice dose reductions are allowed, each to the next lower dose already explored. Any adult subject requiring a reduction of HQP1351 dose below 30 mg QOD should permanently discontinue the study treatment. The study drug can be resumed when the study drug-related toxicities resolve to grade 1 or below.

Dose adjustment may not be performed for grade 1/2 toxicities related to the study drug that can be treated by supportive treatment or don’t influence normal daily activities of subjects, unless QTc prolongation, clinically significant pancreatitis, or other clinical conditions that affect subject's safety at the investigator’s discretion. For intolerable (symptomatic or influencing subject’s normal daily activities) or persistent (unable to be handled with the best supportive care and recovered to grade 1 or baseline after up to 2-week of drug withdrawal) grade 1/2 toxicities related to the investigational product, whether to make dose adjustment will be determined by discussion between the PI and the sponsor.

If a subject experiences grade 3 or 4 investigational drug-related toxicities, the investigational drug should be interrupted immediately. A maximum of 2 weeks (14 days) of drug interruption is permitted to resolve toxicities to Grade 1 or below, or baseline level. The investigational drug can be resumed when the toxicities resolve to Grade 1 or below, or baseline level. Each subject may undergo a maximum of twice dose reductions throughout the study period, and dose increases are generally no longer allowed after dose reductions. Please refer to Table 2-4 for guidance on dose adjustment for study drug-related toxicity.

Table 2: Dose delay and adjustment due to hematologic toxicities

Toxicity / NCI-CTCAE grade	Actions to be taken
**Platelets decreased**	
Grade 1 (≥75 × 10^9/L)	No dose adjustment required.
Grade 2 (≥50 × 10^9/L - <75 × 10^9/L)	1. Interrupt HQP1351 administration until toxicity resolves to Grade ≤ 1.<br>2. Restart HQP1351 administration at the original dose level.
Grade 3 (≥25 × 10^9/L - <50 × 10^9/L)	1. Interrupt HQP1351 administration until toxicity resolves to Grade ≤ 1.<br>2. Restart HQP1351 administration at the original dose level.<br>3. If Grade 3 toxicity recurs, interrupt HQP1351 administration until toxicity resolves to Grade ≤1, and restart HQP1351 administration at a reduced dose level.
Grade 4 (<25 × 10^9/L)	1. Interrupt HQP1351 administration until toxicity resolves to Grade ≤ 1.<br>2. Restart HQP1351 treatment at a reduced dose level.<br>3. If Grade 4 toxicity recurs, stop HQP1351 treatment.
**Neutrophils reduced:**	
Grade 1 (≥1.5 × 10^9/L)	No dose adjustment required.
Grade 2 (≥1.0 - <1.5 × 10^9/L)	No dose adjustment required.
Grade 3 (≥0.5 - <1.0 × 10^9/L)	1. Interrupt HQP1351 administration until toxicity resolves to ≥ 1.0 x 10^9/L.<br>2. Restart HQP1351 administration at the original dose level.<br>3. If Grade 3 toxicity recurs, interrupt HQP1351 administration until toxicity resolves to ≥ 1.0 × 10^9/L.<br>a) If the duration is less than or equal to 7 days, resume administration at the original dose level.<br>b) If the duration is greater than 7 days, resume HQP1351 administration at a reduced dose level.
Grade 4 (<0.5 × 10^9/L)	1. Interrupt HQP1351 administration until toxicity resolves to ≥ 1.0 × 10^9/L.<br>2. Restart HQP1351 administration at a reduced dose level.<br>3. If Grade 4 toxicity recurs, interrupt HQP1351 administration until toxicity resolves to ≥ 1.0 × 10^9/L, and resume administration at a reduced dose level.<br>4. A maximum of two dose reductions is allowed.
**Febrile neutropenia**	
Grade 3: ANC < 1.0 × 10^9/L accompanied by single body temperature >38.3°C or body temperature ≥ 38°C for more than 1 hour	1. Interrupt HQP1351 administration until ANC recovers to ≥ 1.0 × 10^9/L and there is no fever.<br>2. Restart HQP1351 administration at a reduced dose level.<br>3. If febrile neutropenia recurs, stop HQP1351 administration.
Grade 4: life-threatening, urgent intervention indicated	Stop HQP1351 administration.
**Anemia (Hemoglobin)**	

Toxicity / NCI-CTCAE grade	Actions to be taken
Grade 1 (≥ 10.0 - LLN g/dL)	No dose adjustment is required.
Grade 2 (≥ 8.0 -<10.0 g/dL)	No dose adjustment is required.
Grade 3 (<8.0 g/dL)	1. Interrupt HQP1351 administration until toxicity resolves to Grade ≤ 2.<br>**2.** Restart HQP1351 administration at the original dose level.
Grade 4: life-threatening, urgent intervention indicated	Stop HQP1351 administration.

Table 3: Dose delay and adjustment due to hepatic function abnormal

Toxicity / NCI-CTCAE grade	Actions to be taken
**Total bilirubin increased (without ALT/AST increased)**	
Grade 1	1. Monitor liver function once a week.<br>2. No dose adjustment is required.
Grade 2: Total bilirubin > 1.5 × ULN and ≤ 3 × ULN	1. Increase the frequency of liver function monitoring (once every 2-3 days) until it recovers to baseline level.<br>2. Dose adjustment:<br>a) If toxicity resolves to Grade ≤ 1 within 14 days (inclusive), resume HQP1351 administration at the original dose level.<br>b) If toxicity resolves to Grade ≤ 1 beyond 14 days, or toxicity recurs, resume HQP1351 administration at a reduced dose level.<br>c) If toxicity recurs after two dose reductions, stop HQP1351 administration.
Grade 3: Total bilirubin > 3 × ULN-10.0 × ULN	1. Increase the frequency of liver function monitoring (once every 2-3 days) until it recovers to baseline level.<br>2. Dose adjustment:<br>a) If toxicity resolves to Grade ≤ 1 within 14 days (inclusive), resume HQP1351 administration at a reduced dose level.<br>b) If toxicity resolves to Grade ≤ 1 beyond 14 days, or toxicity recurs, stop HQP1351 administration.
Grade 4: Total bilirubin > 10.0 ULN	Stop HQP1351 administration.
Confounding variables and/or other causes of total bilirubin increased should be excluded before dose interruption/reduction. They include, but are not limited to, evidence of obstruction (e.g., typical ALP and GGT increased due to gallbladder or bile duct disease), hyperbilirubinemia (i.e., direct bilirubin ≤ 1 × ULN) due to indirect bilirubin increased as a result of hemolysis or Gilbert syndrome, drug treatment, viral hepatitis, alcoholic or autoimmune hepatitis, and hepatotoxic drugs. For patients with Gilbert syndrome, these dose adjustments apply only to changes in direct bilirubin.	
**AST/ALT increased (without > 2 × ULN total bilirubin increased)**	
Consistent with baseline grade, or increase from baseline Grade 0 to Grade 1	1. Monitor liver function once every 2 weeks.<br>2. No dose adjustment is required.
Grade 2 (>3.0 - 5.0 × ULN)	1. Increase the frequency of liver function monitoring (once every 2-3 days) until it recovers to baseline level.

Toxicity / NCI-CTCAE grade	Actions to be taken
	2. Dose adjustment:<br>a) If toxicity resolves to Grade ≤ 1 within 14 days (inclusive), resume HQP1351 administration at the original dose level.<br>b) If toxicity resolves to Grade ≤ 1 beyond 14 days, or toxicity recurs, resume HQP1351 administration at a reduced dose level.<br>c) Stop HQP1351 administration if toxicity recurs after two dose reductions or if toxicity resolves to baseline level beyond 14 days.
Grade 3 (>5.0 - 20.0 × ULN)	1. Increase the frequency of liver function monitoring (once every 2-3 days) until it recovers to baseline level.<br>2. Dose adjustment:<br>a) If toxicity resolves to ≤ baseline level within 14 days (inclusive), resume HQP1351 administration at a reduced dose level.<br>b) Stop HQP1351 administration if toxicity resolves to ≤ baseline beyond 14 days or toxicity recurs.
Grade 4 (>20 × ULN)	Stop HQP1351 administration.
**AST/ALT increased with total bilirubin increased**	
For patients with normal ALT, AST, and total bilirubin levels at baseline: AST or ALT > 3.0 × ULN, and total bilirubin > 2 × ULN, no evidence of cholestasis<br>or<br>For patients with AST or ALT or total bilirubin increased at baseline: baseline [AST or ALT > 2 × baseline and > 3.0 × ULN] or [AST or ALT 8.0 × ULN], whichever is lower, combined with [total bilirubin > 2 × baseline **and** > 2.0 × ULN]	1. Stop HQP1351 administration.

Table 4: Dose delay and adjustment due to general toxicities

Toxicity / NCI-CTCAE grade	Actions to be taken
**Administration related reaction**	Please refer to 4.3.4.
**Other Toxicities**	
Grade 1	1. Monitor as clinically required.<br>2. No dose adjustment is required.
Grade 2 or 3, clinically significant but uncontrollable with supportive care	1. Interrupt HQP1351 administration for up to 2 weeks (14 days) until toxicity resolves to Grade ≤ 1.<br>2. Resume HQP1351 administration at a reduced dose level and monitor as needed.<br>3. A maximum of two dose reductions is allowed.

Toxicity / NCI-CTCAE grade	Actions to be taken
	4. If Grade 2 or 3 toxicity recurs after two dose reductions, stop HQP1351 administration, and follow up according to the protocol.
Grade 4	Stop HQP1351 administration, and follow up according to the protocol.

4.3.3 Principles for management of serum troponin increased

If a subject develops serum troponin increased during treatment with HQP1351, the following measures are recommended:

- To evaluate cardiac function of the subject and recommend to perform the following examinations:
  - Serial serum troponin test
  - Echocardiogram
- TEE or PET scan to exclude direct heart injury due to tumor (when appropriate).
- Consult the Sponsor to determine whether subjects who continue the study will benefit the most.

Any AEs or SAEs caused by serum troponin increased should be reported as described in 9.2. For subjects with persistent heart injury during treatment with HQP1351, the Sponsor does not recommend special treatments based on the mechanism of action of HQP1351, but recommends routine supportive care and will handle with reference to the advice of internal medicine specialists and cardiologists.

4.3.4 Treatments for patients who develop allergic reactions on or after Day 1

Potential allergic reactions or other inflammatory symptoms may be observed in subjects after HQP1351 is orally administered. Signs and symptoms of inflammation or allergy include: Fever, chills, chilliness, allergic reaction, rash, urticaria, measles, dizziness, hypotension, edema, bronchospasm, tachypnoea, flu-like symptoms, and other reactions.

Patients with symptoms and signs of allergy or similar reactions should be treated according to principles of the institution.

Prophylaxis should be given before each subsequent dose of treatment cycles to patients with allergy or similar reactions, including:

- 500 to 1,000 mg acetaminophen administered orally 30 minutes before dosing;
- 12.5 to 25 mg promethazine administered orally or intravenously 30 minutes before dosing;
- H1-receptor antagonists (e.g., loratadine) 30 minutes before dosing;

- Consider corticosteroids administered orally or intravenously according to the principles of the institution.
- The investigator will decide whether prophylactic administration of H1-receptor antagonists and/or corticosteroids is required. The investigator and the Sponsor will decide whether long-term prophylactic treatment is required in the cycles after allergic reactions are observed.

4.3.5 Guidance for hypertension management during study drug administration

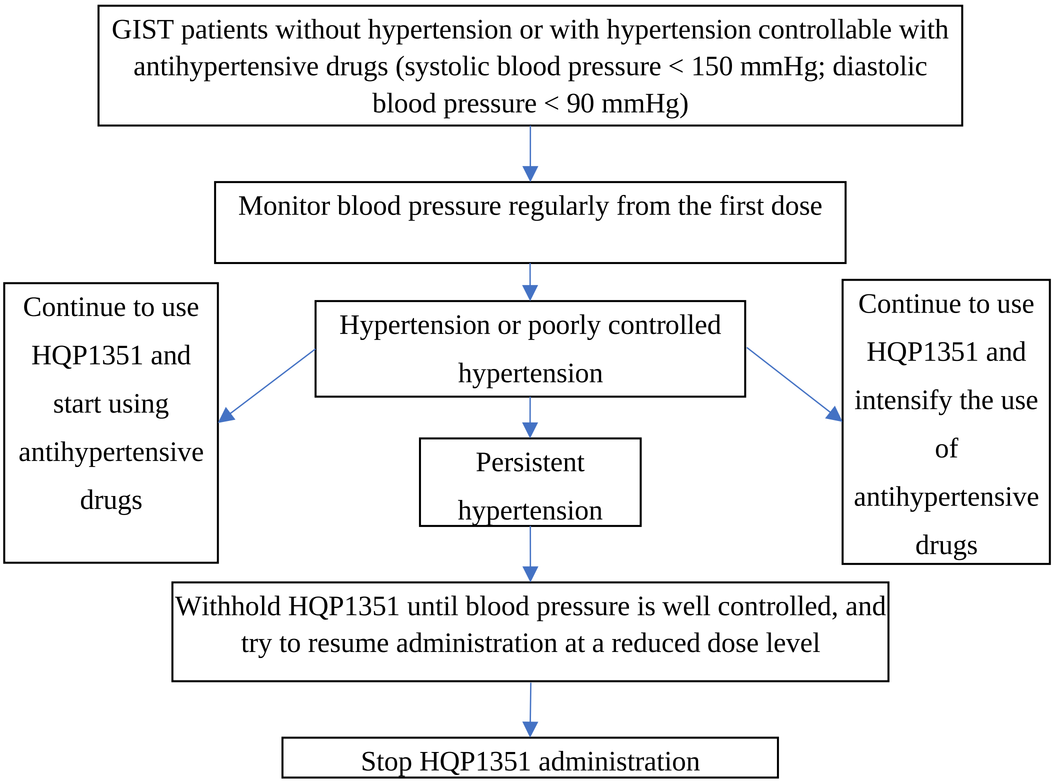

Refer to the Chinese Guidelines for the Prevention and Treatment of Hypertension for the use of antihypertensive drugs.

4.4 End of Study and Efficacy and Safety Analysis

End of study means that all subjects have withdrawn from the study due to PD or other reasons. A final analysis of safety, efficacy and PK data will be performed at the end of study.

In this study, the recommended Phase II dose will be determined by the participating investigator and the sponsor based on safety, tolerability, PK, and available efficacy data.

The data cutoff date is the date on which each subject withdraws from the study due to PD or other reasons, and all data for all subjects acquired prior to this cutoff date will be analyzed.

4.5 Premature Termination of Study

The sponsor can terminate the study at any time for any reason. If it is necessary to terminate the study, the patient should be timely notified to have end-of-treatment (EOT) visit as far as possible, and EOS assessment should be performed in subjects who withdraw prematurely. The investigator must be informed of other procedures to be followed, so as to ensure full consideration of the interests of subjects. The investigator will be responsible for notifying IRB and/or EC of premature termination of the trial.

5 Study Drug

5.1 CMC Introduction to HQP1351

5.1.1 Study drug materials and dosage forms

HQP1351 for clinical trials is tablets, and each tablet contains 10 mg of HQP1351.

Chemical name: 3-[(1H-pyrazolo[3,4-b]pyridin-5-yl)ethynyl]-4-methyl-N-{4-[(4-methylpiperazin-1-yl)methyl]-3-(trifluoromethyl)phenyl]benzamide

Please refer to the Investigator's Brochure for additional information about the chemical and pharmaceutical properties of HQP1351.

5.1.2 Packaging and labeling

The drug is stored in high density polyethylene (HDPE) bottles. Study drug labels will be in Chinese and comply with applicable legal requirements. The label should contain drug storage conditions, but does not contain information about the subject.

5.1.3 Storage and handling

Clinical drugs must be stored in a secure place. See the label for details.

Drug manager must monitor the clinical drug storage area of the site to ensure that the temperature is within the appropriate storage temperature range specified in this protocol or the drug label attached to this protocol. Temperature monitoring document should be preserved.

5.1.4 Usage

HQP1351 Tablets will be orally administered every two days (QOD), in a 28-day cycle. Subjects should take a prescribed dose of drug with meals and 250 ml water at the same time each day. During administration, subjects should maintain a normal diet.

If the study drug is not taken within 4 hours of the specified time, the dose will not be taken but be considered to be missed. This should be recorded in subject diary and reasons should be provided. If the study drug is vomited, it should not be re-administered. It should be considered a missed dose and recorded on the subject's diary with reasons indicated. If a subject delays administration or adjusts the dose for any reasons, the drug should be taken at the originally scheduled date and time.

5.2 Drug Management

On Day 1 of each cycle, subjects will be given the amount of study drug required for up to one cycle.

The investigator should designate a special person to receive, handle and store the study drug. The study drug should be stored in a safe place where only investigator and/or designated personnel who meets the condition can enter. The investigator should adequately manage all used and unused HQP1351, including the receipt record (quantity and conditions) of HQP1351

each shipment, inventory records at the study site, and individual dispensing record of patient. The receipt record should document the quantity received from the Sponsor. The dispensing form of study drug should document the quantity dispensed to patients, including drug batch number, dispensing date, subject number, initials of subject and initials of dispensing person. All unused HQP1351 dispensed to the subject must be returned to the investigator and recorded in the dispensing record (including the quantity of returned study drug, date, and receiving person).

Subjects must return all unused drugs and empty drug containers to the investigator. The CRA of the study will review all HQP1351 supply, use and returning records. Study sites should retain all HQP1351 and containers of used drugs until the CRA informs and instructs the site on how to dispose of and/or destroy all unused HQP1351. Any destruction of HQP1351 should be documented. If possible, after accounting, the sponsor can entrust the hospital to destroy the drugs according to standard procedure for destruction. The sponsor will count any unused investigational products and then entrust the hospital to destroy on the spot.

5.3 Treatment Compliance

5.3.1 Subject diary

Subject diary card will be dispensed to each subject who takes the investigational drug at each visit and collected and reviewed at the next visit.

For a missed or vomited dose, subjects should be instructed not to take an additional dose in an attempt to make up for the missed or vomited dose. Subjects should be instructed to indicate on diary card that they didn’t take the drug and state the reason(s).

5.3.2 Tablet counting

Remaining investigational drugs will be calculated. Subjects will be assessed for compliance with administration of the study drug. If the compliance is < 90%, the sponsor should be notified. For subjects with compliance less than 90%, the investigator and the sponsor will jointly decide whether the subjects continue participating in this study.

5.4 Randomization and Blinding

The randomized part of this study will adopt a randomized and open-label design. The non-randomized part of this study will adopt a non-randomized and open-label study design.

In the randomized part, this study will use the Interactive Web Response System (IWRS) for central randomization. Each subject will be randomly assigned to a 30 mg, 40 mg, or 50 mg dose group. The randomization list used for central randomization will be generated by SAS 9.4 using block randomization and uploaded to the IWRS database. To ensure a balanced number of subjects across dose groups in each phase, the same randomization list will be used at all sites.

Since the randomized part of this study only involves randomization, it is a non-blind open-

label trial, and it is not necessary to blind the investigational products according to the generated randomization list.

5.5 Protocol Deviations

Any deviation from the protocol procedure should be justified in the source documents. According to EC policy and procedure requirements, all serious deviations that may affect patient's safety, study drug use or the evaluation of safety, efficacy and tolerability parameters should be reported to the sponsor (or designated personnel) and EC of study site as soon as possible.

6 Prophylactic Treatment and Concomitant Medications/Treatments

All concomitant medications, concomitant therapies, and concomitant clinical procedures during the period from the subject's signing of the written ICF to the visit 30 days after the last dose of HQP1351 should be recorded in the CRF.

6.1 Pre-treatment Drugs

Information on all prior anticancer treatments (chemotherapy, radiotherapy, immunotherapy, biotherapy, etc.) received by the subject should be collected and recorded in the CRF.

Prophylactic medications should be avoided before Cycle 1. If an AE occurs in Cycle 1, prophylactic drugs may be administered in subsequent cycles.

6.2 Concomitant Medications and Concomitant Therapies

After the start of study treatment, at any time during the study and within 30 days after the last dose, all treatments received by the patient will be considered concomitant therapies. Any treatments for AEs related to the study treatment or unresolved AEs received by patients (including those who withdrew from study treatment) should also be considered as concomitant therapies. In addition to the prohibited therapies or medications, other drugs may be used at the investigator's discretion after discussion and approval by the investigator and the sponsor, but the type, dose, route of administration and treatment cycles should be recorded truthfully in the CRF.

Appropriate routine supportive treatment (including blood transfusions) can be given to subjects with clinical indications according to clinical practice standard.

6.3 Prohibited Medications and Therapies

Patients should be treated according to clinical indications meeting medical standards and at the investigator's discretion. However, the following concomitant medications and therapies are not allowed:

- Surgery;
- Other anti-cancer therapies, including but not limited to: chemotherapeutic agents, immunotherapy (such as vaccines), biological response modifiers, radiotherapy, and/or systemic hormone therapy.
- Immune support drugs, except for 4 weeks before the first planned dose of the investigational drug, a stable dose of corticosteroids may be used.
- Traditional Chinese medicine claimed to have anti-tumor activity;
- Other medications that should be prohibited at the investigator's discretion.

If the subject's clinical condition requires a prohibited medication or treatment, the investigational drug should be discontinued and the subject should withdraw from the trial.

6.4 Potential Drug Interactions

There is no specific data on drug interactions of HQP1351 in humans. *In vitro* studies showed that HQP1351 had a certain inhibitory effect on CYP2C9 and CYP2C19 within the concentration range of 0 - 50 μM and no significant inhibitory effect on CYP1A2, CYP3A4, and CYP2D6. HQP1351 had a potential to induce mRNA expression of CYP1A2 and CYP3A4 at concentrations of 0.3 and 3 μM, and had a potential to induce mRNA expression of CYP2B6 at all test concentrations. CYP3A4 and CYP2C9 are the major metabolic enzymes that catalyze the oxidative metabolism of HQP1351. Based on the available PK data, when HQP1351 is co-administered with CYP2B6 substrate or the inhibitors or inducers of CYP2C9 and CYP3A4, there may be drug-drug interactions. Therefore, the investigator is required to avoid using medicines such as CYP2B6 substrate, strong CYP2C9 inducers or inhibitors and moderate to strong CYP3A4 inducers or inhibitors during the study.

7 Assessment Schedule and Procedure of the Study

The study procedures to be performed for each subject enrolled in the study are described below and detailed in Table 7.

Study procedures should be performed according to the schedule as far as possible. When many procedures are specified at the same time point, PK sample collection should be performed preferably before other procedures. The exact time of the PK procedure must be documented in the subject's medical record. Any irregular procedures necessary for urgent safety-related assessments should take precedence over all routine scheduled procedures.

All laboratory safety tests required in the protocol must be carried out in the laboratory of the study site as far as possible, and if there are special circumstances, the investigator needs to submit an application and decide after discussion with the sponsor. All laboratory results must be recorded in the CRF, and reference ranges used by each laboratory must be provided.

Subjects with any CTCAE grade 3 or 4 laboratory results at withdrawal from the study must be followed up until it is recovered to CTCAE grade 1 or baseline, unless these indexes are unlikely to be improved due to underlying diseases. An unscheduled visit or examination is required if clinically indicated.

Any deviation from the protocol procedures should be justified in the source documents. Any assessment that may influence the safety and efficacy parameters should be notified to the sponsor and the site's Ethics Committee as soon as possible.

7.1 Informed Consent

The investigator must obtain written informed consent form before each potential patient enters the clinical study. Written informed consent form signed and dated by both the patient and the investigator conducting informed discussion must be obtained.

If the subject is incapable of expressing his/her consent, his/her legal representative will give consent on his/her behalf. If both the subject and his/her legal representative are unable to read, an impartial witness must be present to give assistance and witness during the process of informed consent.

The information in the informed consent form should be expressed in a language subjects can understand.

The initial written ICF, all subsequent revised written ICF and written documents must be approved/consented by the IRB/IEC prior to use. Patients or their legal representatives must be timely informed of any new information (if any) related to patients’ willingness to continue the trial.

The informed consent process including signing date must be recorded in the source documents of patients.

7.2 Subject Enrollment and Allocation to Treatment

7.2.1 Subject screening number and randomization number

After signing the informed consent form, the subject will be assigned with a unique screening number. Numbering rule for screening number: This number is composed of 5 digits. The first 2 digits are the number of each site, and the last 3 digits are the number of each subject in the screening order assigned by each site. For example, the screening number for the first subject screened at site 01 is 01001. Once assigned, the screening number cannot be repeatedly used by another subject for any reason.

In the randomization part, the eligibility of a subject who has completed all screening procedures will be determined after the investigator reviews the inclusion/exclusion criteria. After the study site enters the subject’s screening number and date of birth into the IWRS central randomization system, the system will assign the subject to the corresponding treatment group and assign a unique randomization number.

7.2.2 Subject screening visit

Prior to entering the study, subjects who have given informed consent will be assessed to ensure that they meet the criteria (see 3.1 and 3.2).

The following assessments must be completed within 28 days prior to the first dose of the study drug:

- The signing of the written informed consent form.
- Demographics: including age, gender, ethnicity and race.
- Diagnosis, tumor staging and objective disease assessment: including diagnosis, staging, histology/cytology, and lesion sites included in the study, including immunohistochemical testing of SDHB;
- Previous anti-tumor treatments (surgery, radiotherapy, drugs, etc.) (supporting documents of trial-related disease and medical history): all previous anti-tumor treatments, including all surgeries, chemotherapies, biotherapies, immunotherapies and radiotherapies, used for the treatment of tumors or as palliative treatment before the start of the study drug;
- Accompanying and past related medical history (including non-tumor history, history of drug allergy and history of drug dependence, etc.)
- Physical examination: The physical examination will include examination of the general condition, skin and mucous membranes, neck (including thyroid), skull, eyes, ears, nose, mouth and throat, thorax and breasts, lungs, heart, abdomen, back (including spine), lymph nodes, limbs, and basic nervous system assessment;
- Height and weight;
- Vital signs: blood pressure, pulse, respiratory rate and body temperature.

- Complete blood count with differential: hemoglobin, hematocrit, red blood cell count, white blood cell count and differential count, and platelet count. White blood cell differential count include neutrophil, lymphocyte, monocyte, eosinophil, and basophil counts (reported as absolute counts).
- Serum chemistry: C-reactive protein (CRP), sodium, potassium, magnesium, calcium, phosphorus, chloride, bicarbonate or carbon dioxide, albumin, urea or blood urea nitrogen, uric acid, creatinine, total bilirubin, AST, ALT, glucose, alkaline phosphatase, and triglycerides.
- HBV, HCV, HIV, treponema pallidum screening.
- Coagulation test: prothrombin time (PT) and activity, partial thromboplastin time (PTT)/activated partial thromboplastin time (APTT), fibrin degradation product, fibrinogen quantification, and D-dimer.
- Lipase and amylase.
- Urinalysis: Urine glucose, protein, bilirubin, urobilinogen, specific gravity, occult blood, pH value, ketone bodies, nitrite, white blood cells, turbidity, and color. A microscopic analysis will be performed if there is any abnormality in urinalysis.
- Tumor imaging assessment
- Collection of samples related to exploratory biomarker.
- AEs
- Pretreatment medications: All medications and clinically significant non-drug treatments (including physiotherapy, oxygen therapy, and blood transfusion) administered within 28 days prior to the first dose of the study drug must be recorded on the Prior Medications or Surgical and Medical Procedures Page of the CRF, and the CRF should be continuously updated if there are any changes in medications

The following assessments must be completed within 7 days prior to the first dose of the study drug:

- ECOG performance status (See Appendix 1).
- Pregnancy test: Serum pregnancy test (β-HCG) will be performed for all women of childbearing potential.
- Echocardiography: The main parameters of echocardiography are left ventricular ejection fraction and heart structure;
- Troponin I or T.
- 12-lead ECG
- Reviewing inclusion/exclusion criteria.

- AEs
- Pre-treatment medications.

All laboratory safety tests required in the protocol must be carried out in the laboratory of the study site as far as possible, and if there are special circumstances, the investigator needs to submit an application and decide after discussion with the sponsor. All laboratory results must be recorded in the CRF, and reference ranges used by each laboratory must be provided. Investigators should review laboratory data, verify the data that significantly deviate from or beyond the acceptable clinical ranges, timely evaluate abnormal data and indicate whether the data are clinically significant.

7.2.3 Information collection from screen failures

Subjects who do not complete all screening assessments due to dropout or withdrawal for any reason will be considered "screen failures". These subjects will not be assigned to the treatment group, while only their relevant source files will be entered and recorded in the CRF. The investigator will maintain screening records for all subjects in order to assess the screening number and characteristics of the excluded subjects and the reasons for their exclusion. Subjects who fail screening will be replaced.

The following CRF must be completed for screen failures:

- Screening phase allocation page (including reasons for not starting treatment);
- Informed consent;
- Demographics;
- Inclusion / exclusion criteria

7.3 Cycle 1 Visit

7.3.1 C1D1 visit

The following items must be assessed prior to the first dose of HQP1351 *(If laboratory results are available within 7 days prior to the first dose of HQP1351, it is not required to repeat the test)*:

- Vital signs: blood pressure, pulse, respiratory rate and body temperature.
- Physical examination: Disease-related signs can be directly examined.
- Body weight.
- ECOG performance status (See Appendix 1).
- Complete blood count with differential: hemoglobin, hematocrit, red blood cell count, white blood cell count and differential count, and platelet count. White blood cell differential count include neutrophil, lymphocyte, monocyte, eosinophil, and basophil counts (reported as absolute counts).

- Serum chemistry: C-reactive protein (CRP), sodium, potassium, magnesium, calcium, phosphorus, chloride, bicarbonate or carbon dioxide, albumin, urea or blood urea nitrogen, creatinine, uric acid, total bilirubin, AST, ALT, glucose, alkaline phosphatase, and triglycerides.
- Coagulation test: prothrombin time (PT) and activity, partial thromboplastin time (PTT)/activated partial thromboplastin time (APTT), fibrin degradation product, fibrinogen quantification, and D-dimer.
- Lipase and amylase
- Urinalysis: Urine glucose, protein, bilirubin, urobilinogen, specific gravity, occult blood, pH value, ketone bodies, nitrite, white blood cells, turbidity, and color. A microscopic analysis will be performed if there is any abnormality in urinalysis.
- 12-lead ECG
- Serum troponin I or T level.
- Collection of PK blood samples
- AEs
- Concomitant medications (from the first dose to 30 days after the last dose)

7.3.2 C1D2 visit (only for subjects with PK blood samples collected)

HQP1351 is not taken on C1D2, and the following assessments must be completed:

- Vital signs: blood pressure, pulse, respiratory rate and body temperature.
- Collection of PK blood samples
- AEs and concomitant medications

7.3.3 C1D3 visit (only for subjects with PK blood samples collected)

The following assessments must be completed prior to administration of HQP1351:

- Vital signs: blood pressure, pulse, respiratory rate and body temperature.
- Collection of PK blood samples.
- AEs
- Concomitant medications

7.3.4 C1D8 visit

HQP1351 is not taken on C1D8, and the following assessments must be completed:

- Vital signs: blood pressure, pulse, respiratory rate and body temperature.
- Complete blood count with differential: hemoglobin, hematocrit, red blood cell count, white blood cell count and differential count, and platelet count. White blood cell

differential count include neutrophil, lymphocyte, monocyte, eosinophil, and basophil counts (reported as absolute counts).

- Serum chemistry: C-reactive protein (CRP), sodium, potassium, magnesium, calcium, phosphorus, chloride, bicarbonate or carbon dioxide, albumin, urea or blood urea nitrogen, creatinine, uric acid, total bilirubin, AST, ALT, glucose, and alkaline phosphatase.
- AEs and concomitant medications

7.3.5 C1D15 visit

The following assessments must be completed prior to administration of HQP1351:

- Vital signs: blood pressure, pulse, respiratory rate and body temperature.
- Complete blood count with differential: hemoglobin, hematocrit, red blood cell count, white blood cell count and differential count, and platelet count. White blood cell differential count include neutrophil, lymphocyte, monocyte, eosinophil, and basophil counts (reported as absolute counts).
- Serum chemistry: C-reactive protein (CRP), sodium, potassium, magnesium, calcium, phosphorus, chloride, bicarbonate or carbon dioxide, albumin, urea or blood urea nitrogen, creatinine, uric acid, total bilirubin, AST, ALT, glucose, alkaline phosphatase, and triglycerides.
- Lipase and amylase
- 12-lead ECG
- Serum troponin I or T level.
- Collection of PK blood samples.
- AEs
- Concomitant medications

7.3.6 C1D17 (only for subjects with PK blood samples collected)

The following assessments must be completed prior to administration of HQP1351:

- Vital signs: blood pressure, pulse, respiratory rate and body temperature.
- Collection of PK blood samples.

7.3.7 C1D19 (only for subjects with PK blood samples collected)

The following assessments must be completed prior to administration of HQP1351:

- Vital signs: blood pressure, pulse, respiratory rate and body temperature.
- Collection of PK blood samples.

7.3.8 C1D22 visit

HQP1351 is not taken on C1D22, and the following assessments must be completed:

- Vital signs: blood pressure, pulse, respiratory rate and body temperature.
- Complete blood count with differential: hemoglobin, hematocrit, red blood cell count, white blood cell count and differential count, and platelet count. White blood cell differential count include neutrophil, lymphocyte, monocyte, eosinophil, and basophil counts (reported as absolute counts).
- Serum chemistry: C-reactive protein (CRP), sodium, potassium, magnesium, calcium, phosphorus, chloride, bicarbonate or carbon dioxide, albumin, urea or blood urea nitrogen, creatinine, uric acid, total bilirubin, AST, ALT, glucose, alkaline phosphatase, and triglycerides.
- AEs and concomitant medications

7.3.9 C1D27 visit (only for subjects with PK blood samples collected)

The following assessments must be completed prior to administration of HQP1351:

- Vital signs: blood pressure, pulse, respiratory rate and body temperature.
- Collection of PK blood samples.
- AEs and concomitant medications

7.3.10 C1D28 visit (only for subjects with PK blood samples collected)

HQP1351 is not taken on C1D28, and the following assessments must be completed:

- Vital signs: blood pressure, pulse, respiratory rate and body temperature.
- Collection of PK blood samples.
- AEs
- Concomitant medications

7.4 Cycle 2 Visit

7.4.1 C2D1 visit

The following assessments must be completed prior to administration of HQP1351:

- Vital signs: blood pressure, pulse, respiratory rate and body temperature.
- Physical examination: Disease-related signs can be directly examined.
- Body weight.
- ECOG performance status
- Complete blood count with differential: hemoglobin, hematocrit, red blood cell count, white blood cell count and differential count, and platelet count. White blood cell

differential count include neutrophil, lymphocyte, monocyte, eosinophil, and basophil counts (reported as absolute counts).

- Serum chemistry: C-reactive protein (CRP), sodium, potassium, magnesium, calcium, phosphorus, chloride, bicarbonate or carbon dioxide, albumin, urea or blood urea nitrogen, creatinine, uric acid, total bilirubin, AST, ALT, glucose, alkaline phosphatase, and triglycerides.
- Coagulation test: prothrombin time (PT) and activity, partial thromboplastin time (PTT)/activated partial thromboplastin time (APTT), fibrin degradation product, fibrinogen quantification, and D-dimer.
- Lipase and amylase
- Urinalysis: Urine glucose, protein, bilirubin, urobilinogen, specific gravity, occult blood, pH value, ketone bodies, nitrite, white blood cells, turbidity, and color. A microscopic analysis will be performed if there is any abnormality in urinalysis.
- 12-lead ECG
- Serum troponin I or T level.
- Collection of PK blood samples.
- AEs
- Concomitant medications

7.4.2 C2D8 visit

HQP1351 is not taken on C2D8, and the following assessments must be completed:

- Vital signs: blood pressure, pulse, respiratory rate and body temperature.
- Complete blood count with differential: hemoglobin, hematocrit, red blood cell count, white blood cell count and differential count, and platelet count. White blood cell differential count include neutrophil, lymphocyte, monocyte, eosinophil, and basophil counts (reported as absolute counts).
- AEs and concomitant medications

7.4.3 C2D15 visit

The following assessments must be completed prior to administration of HQP1351:

- Vital signs: blood pressure, pulse, respiratory rate and body temperature.
- Complete blood count with differential: hemoglobin, hematocrit, red blood cell count, white blood cell count and differential count, and platelet count. White blood cell differential count include neutrophil, lymphocyte, monocyte, eosinophil, and basophil counts (reported as absolute counts).

- Serum chemistry: C-reactive protein (CRP), sodium, potassium, magnesium, calcium, phosphorus, chloride, bicarbonate or carbon dioxide, albumin, urea or blood urea nitrogen, creatinine, uric acid, total bilirubin, AST, ALT, glucose, alkaline phosphatase, and triglycerides.
- Lipase and amylase
- 12-lead ECG
- Serum troponin I or T level.
- AEs
- Concomitant medications

7.4.4 C2D22 visit

HQP1351 is not taken on C2D22, and the following assessments must be completed:

- Vital signs: blood pressure, pulse, respiratory rate and body temperature.
- Complete blood count with differential: hemoglobin, hematocrit, red blood cell count, white blood cell count and differential count, and platelet count. White blood cell differential count include neutrophil, lymphocyte, monocyte, eosinophil, and basophil counts (reported as absolute counts).
- AEs and concomitant medications

7.5 Visit on Cycle 3 and Subsequent Cycles

7.5.1 Day 1 visit of Cycle 3 and subsequent cycles

The following assessments must be completed prior to administration of HQP1351:

- Vital signs: blood pressure, pulse, respiratory rate and body temperature.
- Physical examination: Disease-related signs can be directly examined.
- Body weight.
- ECOG performance status
- Complete blood count with differential: hemoglobin, hematocrit, red blood cell count, white blood cell count and differential count, and platelet count. White blood cell differential count include neutrophil, lymphocyte, monocyte, eosinophil, and basophil counts (reported as absolute counts).
- Serum chemistry: C-reactive protein (CRP), sodium, potassium, magnesium, calcium, phosphorus, chloride, bicarbonate or carbon dioxide, albumin, urea or blood urea nitrogen, creatinine, uric acid, total bilirubin, AST, ALT, glucose, alkaline phosphatase, and triglycerides.
- Coagulation test: The test will be performed on Day 1 of Cycles 3, 5, 7, and 9, and every

3 months starting from Cycle 9.

- Lipase and amylase: These tests will be performed on Day 1 of Cycles 3, 5, 7, and 9 and once every 3 months since Cycle 9.
- Urinalysis: The test will be performed on Day 1 of Cycles 3, 5, 7, and 9, and once every 3 months since Cycle 9.
- 12-lead ECG and serum troponin I or T levels: The tests will be performed on Day 1 of Cycles 3, 5, 7, and 9, and every 3 months starting from Cycle 9.
- Echocardiography: The examination will be performed on Day 1 of Cycles 3, 5, 7, and 9, and every 3 months starting from Cycle 9. Unscheduled echocardiogram, continuous ultrasound cardiogram and/or other examinations may be performed in the event of a heart murmur or other abnormality in the patient's heart.
- Tumor imaging assessment: CT scans and imaging assessments will be performed within 7 days before Day 1 of odd-numbered cycles (e.g., Cycles 3, 5, 7, 9, etc.).
- Collection of samples related to exploratory biomarkers (Only Cycle 3)
- AEs and concomitant medications

7.5.2 C3D15 and C4D15 visits

The following assessments must be completed prior to administration of HQP1351:

- Vital signs: blood pressure, pulse, respiratory rate and body temperature.
- Complete blood count with differential: hemoglobin, hematocrit, red blood cell count, white blood cell count and differential count, and platelet count. White blood cell differential count include neutrophil, lymphocyte, monocyte, eosinophil, and basophil counts (reported as absolute counts).
- Serum chemistry: C-reactive protein (CRP), sodium, potassium, magnesium, calcium, phosphorus, chloride, bicarbonate or carbon dioxide, albumin, urea or blood urea nitrogen, creatinine, uric acid, total bilirubin, AST, ALT, glucose, alkaline phosphatase, and triglycerides.
- Lipase and amylase
- AEs and concomitant medications

7.6 EOT Visit

EOT visit should be performed within 14 days after the subject's last dose of HQP1351.

If a subject terminates treatment for the reason listed in section 3.3, the investigator should make the subject complete the EOT visit as far as possible and document the reason for treatment discontinuation in the subject's source documents.

During EOT visit, the following assessments must be completed:

- Vital signs: blood pressure, pulse, respiratory rate and body temperature.
- Physical examination: A complete detailed physical examination will be performed.
- ECOG performance status
- Complete blood count with differential
- Serum chemistry
- Coagulation test
- Lipase and amylase
- Urinalysis
- 12-lead ECG
- Serum troponin I or T level
- Echocardiography
- Collection of samples related to exploratory biomarker.
- AEs
- Concomitant medications
- Tumor imaging assessment (if it is less than 4 weeks from the last imaging assessment, no repeat examination is required)

7.7 Visit after EOT

7.7.1 Day 30 visit after the last dose

Safety assessments will be performed approximately 30 days after the last dose of HQP1351, including vital signs, physical examination, ECOG physical status, complete blood count with differential, serum chemistry, AEs, and concomitant medications.

7.7.2 Efficacy follow-up and survival follow-up after the EOT

7.7.2.1 Efficacy follow-up

Subjects who discontinue HQP1351 treatment before PD will be assessed for tumor response every 8 weeks until objective progressive disease or the start of other antitumor therapy.

7.7.2.2 Survival follow-up

After completion of the 30-day safety follow-up or PD follow-up, subsequent antitumor treatment information and survival status will be collected every 3 months until death, lost to follow-up or withdrawal of informed consent.

7.8 PK Studies

The PK analysis will be fully entrusted to Drug Metabolism Research Center, Shanghai Institute of Materia Medica, Chinese Academy of Sciences (address: 6/F, No. 501, Haike Road, Pudong New Area, Shanghai, 201203).

Whole blood collection will be performed by an authorized blood collection personnel or trained medical staff according to standard procedures for venipuncture of the site.

In the standard 3+3 dose escalation part of this study, blood samples from subjects in Cycle 1 will be collected for the PK analysis of HQP1351. In the randomized part, 1-3 study sites will be selected to collect the blood samples of the first 3 enrolled subjects in each dose group in Cycle 1 for the PK analysis of HQP1351 (if not successfully collected from the first 3 subjects, PK samples will be collected from subsequent subjects until PK samples are obtained from 3 subjects). PK blood samples will be collected from all non-randomized adult subjects receiving a fixed dose of 40 mg QOD. Approximately 4 ml of blood sample will be collected at each PK time point for adult subjects. See Table 5a for the time point of PK blood sample collection. PK blood samples will be collected from all adolescent subjects. About 2 ml of blood sample needs to be collected at each PK time point. The PK blood collection time points are shown in Table 5b.

Prior to blood collection, the collection tubes and storage tubes will be prepared and labeled (indicating protocol number, subject initials, subject number, group, time point and date of blood sampling).

The collected whole blood samples should be centrifuged within 15 min (4°C, 1800×g, 10 min), and the start time and end time of centrifugation should be recorded. If a sample cannot be centrifuged in time, it can be refrigerated at 2-8 ℃ for a maximum of 30 min. If there are special circumstances, please make remarks.

Table 5a: PK blood sample collection time points (adult subjects)

**Time Point**	**Pre-dose**	**0.5 hr**	**1hr**	**2hr**	**4hr**	**6hr**	**8hr**	**12hr**	**24 hr**	**48 hr**
**Time window**	**- 1hr**	**± 2 min**	**±5 min**	**±10 min**	**±15 min**	**±15 min**	**±30 min**	**±30 min**	**± 1hr**	**± 1hr**
Cycle 1 Day 1	X[1]	X	X	X	X	X	X	X	X	X[2]
Cycle 1 Day 15	X[1]									
Cycle 1 Day 17	X[1]									
Cycle 1 Day 19	X[1]									
Cycle 1 Day 27	X[1]	X	X	X	X	X	X	X	X	X[3]

[1] Within 1 hour prior to each dose of HQP1351, all time points are calculated from the administration end time of HQP1351.
[2] Blood sample 48 hours after administration of HQP1351on C1D1 will be collected within 1 hour before administration on C1D3.
[3] Blood sample 48 hours after administration of HQP1351 on C1D27 will be collected within 1 hour before administration on C2D1.

Table 6b: PK blood sample collection time points (adolescent subjects)

Time Point	Pre-dose	1hr	2hr	4hr	6hr	8hr	24 hr	48 hr
**Time window**	**- 1hr**	**±5 min**	**±10 min**	**±15 min**	**±15 min**	**±30 min**	**± 1hr**	**± 1hr**
Cycle 1 Day 1	X[1]	X	X	X	X	X	X	X[2]
Cycle 1 Day 27	X[1]	X	X	X	X	X	X	X[3]

[1] Within 1 hour prior to each dose of HQP1351, all time points are calculated from the administration end time of HQP1351.
[2] Blood sample 48 hours after administration of HQP1351on C1D1 will be collected within 1 hour before administration on C1D3.
[3] Blood sample 48 hours after administration of HQP1351 on C1D27 will be collected within 1 hour before administration on C2D1.

7.9 Exploratory Biomarker Study

1) Tumor tissue samples will be collected from all subjects at baseline (i.e., from the screening period until the first dose of HQP1351 in Cycle 1), at PD, or at the EOT for assessment of mutations and/or expression profiles of KIT, PDGFRa, and other tumor-associated genes.
   - Tumor tissue sample collection at baseline: Newly acquired tissues should be preferred, and archival tissue samples (paraffin-embedded blocks or paraffin sections) may be provided if newly acquired tissues are not available.
   - Tumor tissue sample collection at PD or at the EOT: Tumor tissue samples will be collected by fresh puncture or tissue core biopsy (approximately > 3 mm) from patients who voluntarily cooperate with tumor biopsy.
2) In this study, 10 ml of whole blood will be collected from all subjects for ctDNA sequencing at baseline, during treatment (before administration on C3D1), at PD, or at the EOT to assess changes in KIT, PDGFRa, and other tumor-associated gene mutation ratio (MAF).

Sample collection time point for exploratory biomarker analysis is shown in Table 6. Detailed methods for collection, processing, labeling and transportation of samples for exploratory biomarker study are presented in the Central Laboratory's Manual for Study Reference.

Table 7: Sampling Time Point for Exploratory Biomarker

	Baseline (Day -28 to pre-dose on C1D1)	Pre-dose on C3D1	At PD or at the EOT
Tumor tissue	X[1、2]		X[2]
ctDNA (10 mL of whole blood)	X	X[3]	X

1. Newly acquired tissues should be preferred, and archival tissue samples (paraffin-embedded blocks or paraffin sections) may be provided if newly acquired tissues are not available.
2. Optional.
3. All blood sampling should be performed before HQP1351 administration.

Table 8: Schedule of Assessments

**Study stage**	**Screening period**		**Cycle 1**										**Cycle 2**				**Cycles 3, 4 and cycles thereafter**		**EOT**	**Day 30 visit after the last dose**	**Efficacy and survival follow-up after EOT**
Procedure	-28~-1	-7~-1	**D1**	**D2[24]**	**D3[24]**	**D8**	**D15**	**D17[24]**	**D19[24]**	**D22**	**D27[24]**	**D28[24]**	**D1**	**D8**	**D15**	**D22**	**D1**	**D15[21]**	Within 14 days after the last dose		
Time window (day)	NA	NA	NA	NA	NA	±1	±2	±2	±2	±2	±2	NA	±1	±3	±3	±3	±3	±3		±7	±5
Informed consent[1]	X																				
Demographics[2]	X																				
Relevant medical history/history of present illness	X																				
Diagnosis and tumor staging and objective disease assessment[3]	X																				
Prior anti-tumor therapy[4]	X																				
Height and weight[5]	X		X										X				X		X		
Vital signs[6]	X		X	X	X	X	X	X	X	X	X	X	X	X	X	X	X	X	X	X	
Physical examination[7]	X		X										X				X		X	X	
ECOG performance status		X	X										X				X		X	X	
Complete blood count with differential[8]	X		X[19]			X	X			X			X	X	X	X	X	X	X	X	
Serum chemistry[9]	X		X[19]			X	X			X			X		X		X	X	X	X	
HBV, HCV, HIV, TP screening[10]	X																				
Coagulation test[11]	X		X[19]										X				X[20]		X		
Amylase, lipase	X		X[19]				X						X		X		X[20]	X	X		
Urinalysis[12]	X		X[19]										X				X[20]		X		
12-lead ECG		X	X[19]				X						X		X		X[20]		X		
Troponin I or T		X	X[19]				X						X		X		X[20]		X		
Echocardiography[13]		X															X[20]		X		
Pregnancy test[14]		X																			

Study stage	Screening period		Cycle 1										Cycle 2				Cycles 3, 4 and cycles thereafter		EOT	Day 30 visit after the last dose	Efficacy and survival follow-up after EOT
Procedure	-28~-1	-7~-1	**D1**	**D2[24]**	**D3[24]**	**D8**	**D15**	**D17[24]**	**D19[24]**	**D22**	**D27[24]**	**D28[24]**	**D1**	**D8**	**D15**	**D22**	**D1**	**D15[21]**	Within 14 days after the last dose		
Time window (day)	NA	NA	NA	NA	NA	±1	±2	±2	±2	±2	±2	NA	±1	±3	±3	±3	±3	±3		±7	±5
Tumor imaging assessment**[15]**	X																X**[20]**		X		X
Collection of samples related to exploratory biomarker-whole blood**[16]**	X																X[16]		X		
Collection of samples related to exploratory biomarker-tumor tissue**[16]**	X																		X		
PK sample collection (Phase Ib only)[17]			X	X	X		X	X	X		X	X	X								
Pre-treatment/concomitant medications		X	X	X	X	X	X			X	X	X	X	X	X	X	X	X	X	X	
Recording of AEs/SAEs[18]	**X**	X	X	X	X	X	X			X	X	X	X	X	X	X	X	X	X	X	X[22]
Survival follow-up[23]																					X

1. Written informed consent must be obtained before each potential patient enters the clinical study.
2. Demographics include age, gender, ethnicity and race.
3. Diagnosis and tumor staging and objective disease assessment: including diagnosis, staging, histology/cytology, and lesion sites included in the study, including immunohistochemical testing of SDHB.
4. Previous anti-tumor treatments (surgery, radiotherapy, drugs, etc.) (supporting documents of trial-related disease and medical history): all previous anti-tumor treatments, including all surgeries, chemotherapies, biotherapies, immunotherapies and radiotherapies, used for the treatment of tumors or as palliative treatment before the start of the study drug.
5. At the screening visit only, height will be recorded.
6. Vital signs include blood pressure, pulse, respiratory rate and body temperature. Measurements of vital signs prior to administration of HQP1351 on C1D1 are baseline values.
7. Physical examination: A thorough physical examination including the general condition, skin and mucous membranes, neck (including thyroid), skull, eyes, ears, nose, mouth and throat, thorax and breasts, lungs, heart, abdomen, back (including spine), lymph nodes, and limbs and basic nervous system assessment will be performed at the screening visit, end of study/withdrawal visit (within 14 days after the last dose), and at the visit approximately 30 days after the last dose. At other visits, signs related to the disease may be directly examined.
8. Complete blood count with differential: hemoglobin, hematocrit, red blood cell count, white blood cell count and differential count, and platelet count. White blood cell differential count include neutrophil, lymphocyte, monocyte, eosinophil, and basophil counts (reported as absolute counts).
9. Serum chemistry: C-reactive protein (CRP), sodium, potassium, magnesium, calcium, phosphorus, chloride, bicarbonate or carbon dioxide, albumin, urea or blood urea nitrogen, creatinine, uric acid, total bilirubin, AST, ALT, glucose, alkaline phosphatase, and triglycerides.
10. HIV/HBV/HCV screening: HBsAg\anti-HCV antibody\anti-HIV1/2 antibody.
11. Coagulation test: prothrombin time (PT) and activity, partial thromboplastin time (PTT)/activated partial thromboplastin time (APTT), fibrin degradation product, fibrinogen quantification, and D-dimer.

12. Urinalysis: Urine glucose, protein, bilirubin, urobilinogen, specific gravity, occult blood, pH value, ketone bodies, nitrite, white blood cells, turbidity, and color. A microscopic analysis will be performed if there is any abnormality in urinalysis.
13. Echocardiography: The main parameters of echocardiography are left ventricular ejection fraction and heart structure.
14. Premenopausal women of childbearing potential must have a negative pregnancy test within 7 days before the treatment.
15. Tumor imaging assessment: Assessments of tumor response will be performed during the screening period (within 3 weeks prior to the first dose), within 7 days prior to Day 1 of Cycles 3, 5 and odd-numbered cycles thereafter, and at the EOT (if it is > 28 days from the last assessment). All responses [PR and CR] must be confirmed by a repeat assessment not less than 4 weeks and theoretically not later than 5 weeks after the response criteria are met for the first time. During the follow-up period after discontinuation, subjects who have discontinued HQP1351 before PD will be assessed for tumor response every 8 weeks (visit time window +/-5 days) until PD or the start of other antitumor therapy, unless the subject voluntarily withdraws from the study. The same assessment method should be used at baseline and subsequent visits. If a subject experiences dose adjustment or interruption, the frequency of imaging examinations or scheduled imaging dates should not be changed. If there are clinical indications, a bone scan may be performed during screening.
16. The baseline collection time is from the screening period to the first dose of HQP1351 in Cycle 1. Sample collection time point for exploratory biomarker analysis is shown in Table 6.
17. PK blood samples will be collected from the first 3 adult subjects in each dose group, and PK blood samples will be collected from all non-randomized adult subjects receiving a fixed dose of 40 mg QOD. PK samples collected from adult subjects at each time point will be about 4 ml. PK sampling time points are shown in Table 5a. PK blood samples will be collected from all adolescent subjects. About 2 ml of blood sample needs to be collected at each PK time point. The PK blood collection time points are shown in Table 5b.
18. AEs will be collected from the time when the subject signs the ICF to 30 days after the last dose.
19. If the laboratory results at screening are within 7 days prior to the first dose of HQP1351, the test will not be repeated on C1D1.
20. Coagulation test, lipase and amylase, urinalysis, ECG, serum troponin levels and echocardiography: The tests will be performed on Day 1 of Cycles 3, 5, 7 and 9, and every 3 months from Cycle 9. Unscheduled echocardiogram, continuous ultrasound cardiogram and/or other examinations may be performed in the event of a heart murmur or other abnormality in the patient's heart.
21. The Day 15 visit will not be performed in Cycle 5 and subsequent cycles.
22. Any AE or SAE known to the investigator that is believed to be related to the investigational drug 30 days after the last dose of HQP1351 or at any time after the end of the clinical study.
23. After completion of the 30-day safety follow-up or PD follow-up, subsequent antitumor treatment information and survival status will be collected every 3 months until death, lost to follow-up or withdrawal of informed consent.
24. Only for subjects with PK blood samples collected.

8 Efficacy Assessments

Assessments of tumor response will be performed during the screening period (within 4 weeks before the first dose), every 8 weeks after the first treatment (allowing imaging collection up to 7 days before the scheduled efficacy assessment), and at the EOT visit (if it is > 28 days from the last assessment). In any case, if the dosing regimen is adjusted, the visit schedule for efficacy assessment will remain unchanged in accordance with Table 7.

During the follow-up period after discontinuation, subjects without PD will be assessed for tumor response every 8 weeks (visit time window +/-5 days) until PD or the start of other antitumor therapy, unless the subject voluntarily withdraws from the study.

8.1 Response Evaluation Criteria in Solid Tumors (RECIST)

The response in solid tumor patients will be evaluated once every 2 cycles (i.e. 8 weeks) as per the latest version of Response Evaluation Criteria in Solid Tumors: Revised RECIST v1.1 (Eisenhauer, 2009) [15].

8.1.1 Measurable lesions

Tumor lesions: Must be accurately measured in at least one dimension (longest diameter in the plane of measurement is to be recorded) with a minimum size of:

- 10 mm by CT scan (CT scan slice thickness no greater than 5 mm);
- 10 mm caliper measurement by clinical exam (lesions which cannot be accurately measured with calipers should be recorded as non-measurable);
- 20 mm by chest X-ray;
- 15 mm by traditional techniques (medical photography [skin or mouth lesions], palpation, plain film X-rays, CT or MRI). The shortest axis of measurable lesions should be at least double of the reconstruction interval (e.g., if reconstruction interval is 10 mm, the shortest axis of measurable lesions should be 20 mm) by CT scan; or
- ≥ 10 mm by spiral CT scan.

Malignant lymph nodes: To be considered pathologically enlarged and measurable, a lymph node must be ≥ 15 mm in short axis when assessed by CT scan (CT scan slice thickness recommended to be no greater than 5 mm). At baseline and in follow-up, only the short axis will be measured and followed.

8.1.2 Non-measurable lesions

All other lesions, including small lesions (longest diameter < 10 mm or short axis of pathological lymph nodes ≥ 10 mm and < 15 mm) as well as non-measurable lesions. Non-measurable lesions include:

- Meningeal disease;

- Ascites;
- Pleural or pericardial effusion;
- Inflammatory breast diseases;
- Lymphangitic involvement of skin or lung;
- Abdominal masses/abdominal organomegaly that cannot be identified by physical examination or measured by imaging techniques.

8.1.3 Special considerations regarding lesion measurability

Bone lesions, cystic lesions, and lesions previously treated with local therapy require particular comment.

8.1.3.1 Bone lesions

- Bone scan, PET scan or plain films are not considered adequate imaging techniques to measure bone lesions. However, these techniques can be used to confirm the presence or disappearance of bone lesions;
- Lytic bone lesions or mixed lytic-blastic lesions, with identifiable soft tissue components, that can be evaluated by cross sectional imaging techniques such as CT or MRI can be considered as measurable lesions if the soft tissue component meets the definition of measurability described above;
- Blastic bone lesions are non-measurable.

8.1.3.2 Cystic lesions:

- Lesions that meet the criteria for radiographically defined simple cysts should not be considered as malignant lesions (neither measurable nor non-measurable) since they are, by definition, simple cysts;
- “Cystic lesions” thought to represent cystic metastases can be considered as measurable lesions, if they meet the definition of measurability described above. However, if noncystic lesions are present in the same patient, these are preferred for selection as target lesions.

8.1.3.3 Locally treated lesions

Tumor lesions situated in a previously irradiated area, or in an area subjected to other loco-regional therapy, are usually not considered measurable unless there has been demonstrated progression in the lesion (defined as the longest diameter increased by ≥ 20% after the last radiotherapy).

8.1.4 Measurement of lesions

All measurements should be recorded in metric notation, using calipers if clinically assessed. All baseline evaluations should be performed as close as possible to the treatment start and

never more than 28 days before the beginning of the treatment.

8.1.5 Method of assessment

The same method of assessment and the same technique should be used to characterize each identified and reported lesion at baseline and during follow-up. Imaging-based evaluation should always be done rather than clinical examination unless the lesion(s) being followed cannot be imaged but are assessable only by clinical examination.

8.1.5.1 CT and MRI

CT is the best currently available and reproducible method to measure lesion(s) selected for response assessment. This guideline has defined measurability of lesions on CT scan based on the assumption that CT slice thickness is 5 mm or less. When CT scans have slice thickness greater than 5 mm, the minimum length for a measurable lesion should be twice the slice thickness. MRI is also acceptable in certain situations (e.g., for body scans). More details concerning the use of both CT and MRI for assessment of objective tumor response evaluation are provided in the literature published by Eisenhauer in 2009[11].

8.1.5.2 Clinical lesion

Clinical lesions will only be considered measurable when they are superficial and ≥ 10 mm in diameter as assessed using calipers (e.g., skin nodules). For the case of skin lesions, documentation by colour photography including a ruler to estimate the size of the lesion is suggested. As noted above, when lesions are evaluated by both clinical exam and imaging, imaging evaluation should be undertaken since it is more objective and may also be reviewed at the end of the study.

8.1.5.3 Chest X-ray

Chest CT is preferred over chest X-ray, particularly when progression is an important endpoint, since CT is more sensitive than X-ray, particularly in identifying new lesions. However, lesions on chest X-ray may be considered measurable if they are clearly defined and surrounded by aerated lung.

8.1.5.4 Ultrasound

Ultrasound is not useful in assessment of lesion size and should not be used as a method of measurement. Ultrasound examinations cannot be reproduced in their entirety for independent review at a later date and, because they are operator dependent, it cannot be guaranteed that the same technique and measurements will be taken from one assessment to the next. If new lesions are identified by ultrasound in the course of the study, confirmation by CT or MRI is advised. If there is concern about radiation exposure at CT, MRI may be used instead of CT in selected instances.

8.1.5.5 Endoscopy and laparoscopy

The utilization of these techniques for objective tumor evaluation is not advised. However, they

can be useful to confirm complete pathological response when biopsies are obtained or to determine relapse.

8.1.5.6 Tumor markers

Tumor markers alone cannot be used to assess objective tumor response. If markers are above the upper limit of normal at baseline, however, they must normalize for a patient to be considered in complete response.

8.1.5.7 Cytology and histology

These techniques can be used to differentiate between PR and CR if applicable (for example, residual lesions in tumor types such as germ cell tumors, where known residual benign tumors can remain). When effusions are known to be a potential adverse effect of treatment (e.g. with certain taxane compounds or angiogenesis inhibitors), the cytological confirmation of the neoplastic origin of any effusion that appears or worsens during treatment can be considered if the measurable tumor has met criteria for response or SD in order to differentiate between response (or SD) and PD.

8.1.6 Tumor response evaluation

8.1.6.1 Assessment of overall tumor burden and measurable disease

To assess objective response or future progression, it is necessary to estimate the overall tumor burden at baseline and use this as a comparator for subsequent measurements. Measurable disease is defined as the presence of at least one measurable lesion. The study doesn't require the presence of measurable lesions at baseline. For this trial, if the patient does not have a measurable lesion (according to RECIST v1.1 criteria), the response of non-measurable lesions will be evaluated according to the specifications in 8.1.2 (e.g., present or absent or new non-measurable lesion), and the assessment of the target lesion will not be applicable.

8.1.6.2 Baseline documentation of "target" and "non-target" lesions

When more than one measurable lesion is present at baseline, all lesions up to a maximum of five lesions total (and a maximum of two lesions per organ) representative of all involved organs should be identified as target lesions and will be recorded and measured at baseline (this means in instances where patients have only one or two organ sites involved a maximum of two and four lesions respectively will be recorded).

Target lesions should be selected on the basis of their size (lesions with the longest diameter), be representative of all involved organs, but in addition should be those that lend themselves to reproducible repeated measurements. It may be the case that, on occasion, the largest lesion does not lend itself to reproducible measurement in which circumstance the next largest lesion which can be measured reproducibly should be selected.

Lymph nodes merit special mention since they are normal anatomical structures which may be visible by imaging even if not involved by tumor. As noted above, pathological lymph nodes

which are defined as measurable nodes and may be identified as target lesions must meet the criterion of a short axis of ≥ 15 mm by CT scan. Only the short axis of these nodes will contribute to the baseline sum. The short axis of the node is the diameter normally used by radiologists to judge if a node is involved by solid tumor. Nodal size is normally reported as two dimensions in the plane in which the image is obtained (for CT scan this is almost always the axial plane; for MRI the plane of acquisition may be axial, saggital or coronal). The smallest of these measures is the short axis. For example, an abdominal node which is reported as being 20 mm × 30 mm has a short axis of 20 mm and qualifies as a malignant, measurable node. In this example, 20 mm should be recorded as the node measurement. All other pathological nodes (those with short axis ≥ 10 mm but < 15 mm) should be considered non-target lesions. Nodes that have a short axis of < 10 mm are considered non-pathological and should not be recorded or followed.

A sum of the diameters (longest for non-nodal lesions, short axis for nodal lesions) for all target lesions will be calculated and reported as the baseline sum of diameters. If lymph nodes are to be included in the sum, then as noted above, only the short axis is added into the sum. The baseline sum diameters will be used as reference to further characterize any objective tumor regression in the measurable dimension of the disease.

All other lesions (or lesion sites) including pathological lymph nodes can be identified as non-target lesions and do not need to be measured, but they should also be recorded at baseline. These lesions should be recorded as "present", "absent", or in rare cases "unequivocal progression". In addition, it is possible to record multiple non-target lesions involving the same organ as a single item on the case record form.

8.1.7 Response criteria

This section provides the definitions of the criteria used to determine objective tumor response for target lesions.

8.1.7.1 Evaluation of target lesions

- Complete response (CR): Disappearance of all target lesions. Any pathological lymph nodes (whether target or non-target) must have reduction in short axis to < 10 mm.
- Partial response (PR): At least a 30% decrease in the sum of diameters of target lesions, taking as reference the baseline sum diameters.
- Progressive disease (PD): At least a 20% increase in the sum of diameters of target lesions, taking as reference the smallest sum on study (this includes the baseline sum if that is the smallest on study). In addition to the relative increase of 20%, the sum must also demonstrate an absolute increase of at least 5 mm (the appearance of one or more new lesions is also considered progression).
- Stable disease (SD): Neither sufficient shrinkage to qualify for PR nor sufficient increase to qualify for PD, taking as reference the smallest sum diameters while on study.

8.1.7.2 Special notes on the assessment of target lesions of lymph node

Lymph nodes: Actual short axis measurement should be recorded (measured in the same anatomical plane as the baseline examination), even if the nodes regress to below 10 mm. This means that when lymph nodes are included as target lesions, the 'sum' of lesions may not be zero even if complete response criteria are met, since a normal lymph node is defined as having a short axis of < 10 mm. Target nodal lesions considered target lesions will be recorded in a separate section where, in order to qualify for CR, each node must achieve a short axis <10 mm. For PR, SD and PD, the actual short axis measurement of the nodes is to be included in the sum of target lesions.

8.1.7.3 Target lesions that become 'too small to measure'

While on study, all lesions (nodal and non-nodal) recorded at baseline should have their actual measurements recorded at each subsequent evaluation, even when very small (e.g., 2 mm). However, sometimes lesions or lymph nodes which are recorded as target lesions at baseline become so faint on CT scan that the radiologist may not feel comfortable assigning an exact measure and may report them as being 'too small to measure'. When this occurs it is important that a value be recorded on the CRF. If it is the opinion of the radiologist that the lesion has likely disappeared, the measurement should be recorded as 0 mm. If the lesion is believed to be present and is faintly seen but too small to measure, a default value of 5 mm should be assigned. (Note: It is less likely that this rule will be used for lymph nodes since they usually have a definable size when normal and are frequently surrounded by fat such as in the retroperitoneum; however, if a lymph node is believed to be present and is faintly seen but too small to measure, a default value of 5 mm should be assigned in this circumstance as well). This default value is derived from the 5 mm CT slice thickness (but should not be changed with varying CT slice thickness). The measurement of these lesions is potentially non-reproducible, therefore providing this default value will prevent false responses or progressions based upon measurement error. To reiterate, however, if the radiologist is able to provide an actual measure, that should be recorded, even if it is below 5 mm.

8.1.7.4 Lesions that split or coalesce on treatment

When non-nodal lesions 'fragment', the longest diameters of the fragmented portions should be added together to calculate the target lesion sum. Similarly, as lesions coalesce, a plane between them may be maintained that would aid in obtaining maximal diameter measurements of each individual lesion. If the lesions have truly coalesced such that they are no longer separable, the vector of the longest diameter in this instance should be the maximal longest diameter for the 'coalesced lesion'.

8.1.8 Evaluation of non-target lesions

This section provides the definitions of the criteria used to determine the tumor response for the group of non-target lesions. While some non-target lesions may actually be measurable,

they need not be measured and instead should be assessed only qualitatively at the time points specified in the protocol.

- Complete response (CR): Disappearance of all non-target lesions and normalization of tumor marker level. All lymph nodes must be non-pathological in size (<10 mm short axis).
- Non-CR/Non-PD: Persistence of one or more non-target lesion(s) and/or maintenance of tumor marker level above the normal limits.
- Progressive disease (PD): Unequivocal progression of existing non-target lesions (Note: the appearance of one or more new lesions is also considered progression).

8.1.8.1 Special notes on assessment of progression of non-target lesion

The concept of progression of non-target disease requires additional explanation as follows:

When the patient also has measurable non-target disease

In this setting, to achieve 'unequivocal progression' on the basis of the non-target disease, there must be an overall level of substantial worsening in non-target disease such that, even in presence of SD or PR in target disease, the overall tumor burden has increased sufficiently to merit discontinuation of therapy. A modest 'increase' in the size of one or more non-target lesions is usually not sufficient to quality for unequivocal progression status. The designation of overall progression solely on the basis of change in non-target disease in the face of SD or PR of target disease will therefore be extremely rare.

When the patient has only non-measurable disease

The same general concepts apply here as noted above, however, in this instance there is no measurable disease assessment to factor into the interpretation of an increase in non-measurable disease burden. Because worsening in non-target disease cannot be easily quantified (by definition: if all lesions are truly non-measurable) a useful test that can be applied when assessing patients for unequivocal progression is to consider if the increase in overall disease burden based on the change in non-measurable disease is comparable in magnitude to the increase that would be required to declare PD for measurable disease: i.e. an increase in tumor burden representing an additional 73% increase in 'volume' (which is equivalent to a 20% increase diameter in a measurable lesion). Examples include that pleural effusion increases from trace to large, lymphatic involvement spreads from primary to distal site or may be described as necessary to change in treatment in the protocol. If 'unequivocal progression' is seen, the patient should be considered to have had overall PD at that point. While it would be ideal to have objective criteria to apply to non-measurable disease, the very nature of that disease makes it impossible to do so, therefore the increase must be substantial.

8.1.8.2 New lesions

The appearance of new malignant lesions denotes disease progression; therefore, some

comments on new lesions are important. There are no specific criteria for the identification of new radiographic lesions; however, the finding of a new lesion should be unequivocal: i.e. not attributable to differences in scanning technique, change in imaging modality or findings thought to represent something other than tumor (for example, some ‘new’ bone lesions may be simply healing or flare of pre-existing lesions). This is particularly important when the patient's baseline lesions show partial or complete response. For example, necrosis of a liver lesion may be reported on a CT scan report as a 'new' cystic lesion, which it is not.

A lesion identified on a follow-up study but not found at baseline is considered a new lesion and will indicate disease progression. An example of this is the patient who has visceral disease at baseline and while on study has a CT or MRI brain ordered which reveals metastases. The patient’s brain metastases are considered to be evidence of PD even if he/she did not have brain imaging at baseline.

If a new lesion is equivocal, for example because of its small size, continued therapy and follow-up evaluation will clarify if it represents truly new disease. If repeat scans confirm there is definitely a new lesion, then progression should be declared using the date of the initial scan.

While FDG-PET response assessments need additional study, it is sometimes reasonable to incorporate the use of FDG-PET scanning to complement CT scanning in assessment of progression (particularly possible ‘new’ disease). New lesions on the basis of FDG-PET imaging can be identified according to the following algorithm:

- Negative FDG-PET at baseline, with a positive FDG-PET at follow-up is a sign of PD based on a new lesion.
- No FDG-PET at baseline and a positive FDG-PET at follow-up:
    a. If the positive FDG-PET at follow-up corresponds to a new site of disease confirmed by CT, this is PD.
    b. If the positive FDG-PET at follow-up is not confirmed as a new site of disease on CT, additional follow-up CT scans are needed to determine if there is truly progression occurring at that site (if so, the date of PD will be the date of the initial abnormal FDG-PET scan). If the positive FDG-PET at follow-up corresponds to a pre-existing site of disease on CT that is not progressing on the basis of the anatomic images, this is not PD.

8.1.9 Evaluation of best overall response

The best overall response is the best response recorded from the start of the study treatment until the EOT taking into account any requirement for confirmation. The patient’s best overall response assignment will depend on the findings of both target and non-target disease and will also take into consideration the appearance of new lesions.

8.1.9.1 Time point response

Table 8 provides a summary of the overall response status calculation at each time point for patients who have measurable disease at baseline. When patients have non-measurable (therefore non-target) disease only, Table 9 is to be used.

Table 9: Time point response-patients with target (+/- non-target) disease

Target lesions	Non-target lesions	New lesions	Overall response
CR[1]	CR	No	CR
CR	Non-CR/non-PD[2]	No	PR
CR	Not evaluated	No	PR
PR[3]	Non-PD or not all evaluated	No	PR
SD[4]	Non-PD or not all evaluated	No	SD
Not all evaluated	Non-PD	No	NE[5]
PD	Any	Yes or No	PD
Any	PD	Yes or No	PD
Any	Any	Yes	PD

[1] Complete response; [2] progressive disease; [3] partial response; [4] stable disease; and [5] not evaluable

Table 10: Time point response-patients with non-target lesions only

Non-target lesions	New lesions	Overall response
CR[1]	No	CR
Non-CR/non-PD[2]	No	Non-CR or non-PD[3]
Not all evaluated	No	Not evaluable[4]
Unequivocal PD	Yes or No	PD
Any	Yes	PD

[1] Complete response; [2] Progressive disease; [3] 'Non-CR/non-PD' is preferred over 'SD' for non-target disease. Since SD is increasingly used as endpoint for assessment of efficacy in some trials so to assign this category when no lesions can be measured is not advised.

8.1.9.2 Missing assessments and non-evaluable designation

When no imaging/measurement is done at all at a particular time point, the patient is not evaluable (NE) at that time point. If only a subset of lesion measurements is made at an assessment, usually the case is also considered NE at that time point, unless a convincing argument can be made that the contribution of the individual missing lesion(s) would not change the assigned time point response. This would be most likely to happen in the case of PD. For example, if a patient had a baseline sum of 50 mm with three measured lesions and at follow-up only two lesions were assessed, but those gave a sum of 80 mm, the patient will have achieved PD status, regardless of the contribution of the missing lesion.

8.1.9.3 Best overall response: all time points

The best overall response is determined once all the data for the patient is known. The best overall response of the study is defined as the best response across all time points (for example, a patient who has SD at first assessment, PR at second assessment, and PD on last assessment has a best overall response of PR). When SD is believed to be best response, it must also meet the protocol specified minimum time from baseline. If the minimum time is not met when SD is otherwise the best time point response, the patient's best response depends on the subsequent assessments. a patient who has SD at first assessment, PD at second and does not meet minimum duration for SD, will have a best response of PD. The same patient lost to follow-up after the first SD assessment would be considered not evaluable.

8.1.9.4 Special notes on efficacy assessment

When nodal disease is included in the sum of target lesions and the nodes decrease to 'normal' size (<10 mm), they may still have a measurement reported on scans. This measurement should be recorded even though the nodes are normal in order not to overstate progression should it be based on increase in size of the nodes. As noted earlier, this means that patients with CR may not have a total sum of 'zero' on the case report form (CRF).

Patients with a global deterioration of health status requiring discontinuation of treatment without objective evidence of disease progression at that time should be reported as "symptomatic deterioration". Every effort should be made to document objective progression even after discontinuation of treatment. Symptom deterioration is not an assessment description of an objective response: it is a reason for discontinuation of treatment. The objective response status of such patients is to be determined by evaluation of target and non-target disease as shown in Table 8 and 9.

In some circumstances it may be difficult to distinguish residual disease from normal tissue. When the evaluation of complete response depends upon this determination, it is recommended that the residual lesion be investigated (fine needle aspirate/biopsy) before assigning a status of complete response. FDG-PET may be used to upgrade a response to a CR in a manner similar to a biopsy in cases where a residual radiographic abnormality is thought to represent fibrosis or scarring.

For equivocal findings of progression (e.g. very small and uncertain new lesions; cystic changes or necrosis in existing lesions), treatment may continue until the next scheduled assessment. If at the next scheduled assessment, progression is confirmed, the date of progression should be the earlier date when progression was suspected.

8.1.10 Frequency of tumor re-evaluation

Frequency of tumor re-evaluation by imaging while on treatment and follow-up (if applicable) is once every 2 cycles (56 days). Patients whose efficacy is assessed as CR, PR, or SD will be followed every 2 cycles after discontinuation of treatment until PD, initiation of a new anti-

tumor therapy, or withdrawal of consent, whichever occurs first. Some non-target organs may be evaluated less frequently. For example, bone scan re-evaluation may only be required if the target lesion is confirmed as CR or if progression of the bone lesion is suspected.

8.1.11 Response confirmation

All responses [PR and CR] must be confirmed by a repeat assessment not less than 4 weeks and theoretically not later than 5 weeks after the response criteria are met for the first time.

9 Adverse events (AEs) and serious adverse events (SAEs)

The investigator is responsible for observing and recording AEs (including SAEs) and other safety information as required by the protocol.

9.1 Definitions

9.1.1 Adverse events (AEs)

An adverse event (AE) is an untoward medical condition or worsening of a pre-existing medical condition that occurs after the use of a drug, whether related to the study drug or not. An AE may be a diagnosis or, in the absence of a diagnosis, a symptom (e.g., nausea, chest pain), a sign (e.g., tachycardia, hepatomegaly), or an abnormal test result (e.g., laboratory test, ECG, etc.). If a diagnosis is later available, the diagnosis will be applied instead of symptoms, signs, or abnormal test results.

The term AE includes both serious and non-serious adverse events.

9.1.2 Serious adverse events (SAEs)

An AE is considered as an SAE when it meets one or more of the following criteria:

- Resulting in death,
- Life-threatening--Note: "Life-threatening" refers to any event that, in the opinion of the investigator, causes the subject to be at risk of death immediately due to the reaction, and does not include an event that has a potential to cause death if it is more severe. For example, drug-induced hepatitis that does not result in liver failure cannot be considered life-threatening because it may be fatal.
- Resulting in hospitalization or prolongation of existing hospitalization--Note: In general, inpatient hospitalization specifically refers to that patients or subjects stay (usually involving at least one stay overnight) in the hospital or emergency room and require observation and/or treatment, which is not performed in the physician's office or outpatient department. Complications that occur during hospitalization are AEs. This event is serious if a complication prolongs the time for the primary treatment, or meets other criteria for seriousness. When in doubt as to whether “hospitalization” occurred or is necessary, the AE is considered serious. Hospitalization for a condition that has already existed and does not worsen from baseline is not considered an AE. Hospitalization or prolongation of current hospitalization due to elective surgery, routine clinical procedures, social reasons, or personal convenience reasons need not be recorded as an AE. However, if the event meets the AE definition, it still needs to be reported as a “serious” or “non-serious” AE according to the usual criteria.
- Resulting in persistent or significant disability/incapacity--Note: The term disability is a substantial disruption of the patient’s ability to carry out normal life functions.
- Congenital anomaly/birth defect.

- Important medical event: Based on reasonable medical and scientific judgment, events that may not be immediately life-threatening or result in death or hospitalization but might jeopardize the patient or might require intervention to prevent one of the other outcomes listed in the definition above. For example, rescue therapy administered due to allergic bronchospasm in the emergency room or at home, serious hematologic disorder or convulsions not resulting in hospitalization, those leading to drug dependence or abuse, etc.

9.2 Collection and Reporting of AEs

All AEs required to be collected by the protocol must be recorded in the CRF.

9.2.1 Time frame for collection of AEs

AEs/SAEs in this study will be collected from the patient’s giving informed consent until 30 days after the last dose.

9.2.2 Follow-up of AEs

After the initial AE/SAE report is submitted, the investigator is required to proactively follow up with each patient and provide further information to the sponsor.

From the perspective of protecting subject's safety, the investigator must follow up all AEs until the final outcome or a stable condition. Follow-up information on AEs obtained after the end of the study (e.g. after database lock) does not necessarily need to be recorded in the CRF. If necessary, the sponsor reserves the right to obtain additional follow-up information of subjects whose AE is persistent at the end of the study. Follow-up information on SAEs occurring after database lock should be reported to the Sponsor’s pharmacovigilance team. The investigator should ensure that follow-up provides all supplementary information that clarifies the causality and nature of AEs or SAEs. This may include additional laboratory tests, clinical investigations or consultations with specialists in other health care facilities.

If a patient dies during the clinical trial period or during the protocol-specified follow-up period, the sponsor has the right to obtain disease course information related to the death, including histopathological results.

9.2.3 AEs based on examinations and tests

Examination and laboratory abnormalities (e.g., hematology, serum chemistry, urinalysis, ECG, physical examination, and vital signs) identified at screening will be considered present before the signing of ICF and not be recorded as AEs. Abnormalities with clinical significance at screening can be recorded as related medical histories according to the medical judgment of the investigator.

Any abnormal laboratory finding (e.g., clinical chemistry and hematology) or deterioration in vital signs that is judged by the investigator to be clinically significant will also be recorded as an AE/SAE if it meets the definition in Section 9.1. The investigator will determine if an

abnormal laboratory finding or other abnormal assessment is clinically significant with his/her medical and scientific judgment. If the abnormality is associated with clinical signs/symptoms, then the signs/symptoms should be reported as AEs/SAEs and the relevant laboratory values/vital signs as supplementary information. An examination or test abnormality that meets any of the following will generally be considered clinically significant and will be reported as an AE:

- This abnormality suggests a new or worsening disease or organ toxicity from baseline;
- This abnormality needs dose and administration adjustment of the study drug, such as change of dose, drug withdrawal, etc.;
- This abnormality requires additional active intervention, such as increase or change of concomitant medications, close observation, more frequent follow-up assessments, and further diagnostic tests, etc.

Laboratory test values with a clear relationship to progressive disease or deterioration of vital signs will not be reported as AEs/SAEs.

9.2.4 Treatment failure or PD

The “treatment failure” itself is not reported as an AE. Signs and symptoms or clinical sequelae resulting from treatment failure will be reported if they meet the definition of an AE or SAE (including description).

Specific disease progression won't be reported as an AE. Assessment of PD should be recorded in the corresponding module of the CRF, not the AE module. Signs and symptoms or clinical sequelae resulting from PD should be reported if they meet the definition of an AE or SAE.

9.2.5 Severity grading of AEs

The severity of all AEs and laboratory abnormalities will be graded according to NCI CTCAT v4.03. For AEs that cannot be graded according to NCI CTCAT v4.03, the severity of each AE will be summarized by clinical description, respectively:

- Grade 1: Mild; asymptomatic or mild symptoms; clinical or diagnostic observations only; or intervention not indicated.
- Grade 2: Moderate; minimal, local or noninvasive intervention indicated; limiting age-appropriate instrumental ADL (For example, shopping for groceries or clothes, using the telephone, managing money, etc.).
- Grade 3: Severe or medically significant but not immediately life-threatening; hospitalization or prolongation of hospitalization indicated; disabling; limiting self care ADL (For example, bathing, dressing and undressing, feeding self, using the toilet, taking medications), and not bedridden.
- Grade 4: Leading to life-threatening consequences; and emergency treatment is needed.

- Grade 5: Death related to AEs.

Attention should be paid to distinction of severity and intensity of an AE. The term “severe” is used to describe the intensity of an event, which does not necessarily have to be a serious adverse event (SAE). For example, headache may be severe in intensity (Grade 3), but not necessarily a serious adverse event, unless it meets the criteria of serious adverse events.

9.2.6 Correlation between an AE and the study drug

Investigators should evaluate the causal relationship of each case of AE to the study drug based on medical and scientific judgment with reference to the following criteria:

(1) Related: occurrence of the event has reasonable temporal relationship with administration, and the event is consistent with known property of the investigational drug, which is improved after dose reduction or interruption and recurs after re-administration and the subject's clinical condition or other treatments can not explain the event.

(2) Possibly related: occurrence of the event has reasonable temporal relationship with administration, the event is consistent with known property of study drug, and the subject's clinical condition or other treatment may also cause the event.

(3) Unlikely related: the temporal relationship between the occurrence of the event and administration is uncertain, and the event is not likely to be consistent with known property of the study drug, and the subject's clinical condition or other treatment may also cause the event.

(4) Not related: occurrence of the event has no reasonable temporal relationship with administration, the event is not consistent with known property of the study drug, and the subject's clinical condition or other treatment may also cause the event. The event lessens or disappears when the disease condition is improved or other treatment is discontinued.

9.3 Reporting SAEs

All SAEs must be reported, regardless of whether they are related to the study drug.

If any SAE is found during the study, the investigator or site staff must complete and email the “SAE Form for Investigator Reporting to Sponsor” to the sponsor within 24 hours of learning of the event (or immediately for death cases).

Contact information of Sponsor's pharmacovigilance team:

Phone: 15921794486

Email: drugsafety@healthquestpharma.com

Address: Building 5201, No. 338, Jialilue Road, Zhangjiang High-Tech Park, Pudong New District, Shanghai

- According to requirements, the sponsor or its designated agent will conduct follow-up together with clinical trial institution staff to obtain additional information and complete

reporting of the entire event;

- According to national regulations, the investigator is required to provide the Ethics Committee with suspected unexpected serious adverse reactions (SUSARs).

9.4 Reporting Requirements after the End of Clinical Trial

The investigator could report any SAE that occurs at any time after the end of study and is considered to be related/possibly related to the study drug to the Sponsor's pharmacovigilance team. Follow-up information on SAEs after database lock can also be reported to the Sponsor's pharmacovigilance team.

9.5 Guideline for Supportive Therapy

Subjects will be allowed to receive appropriate supportive measures as deemed necessary by the attending physician, including but not limited to the following:

(1) Diarrhea: Prompt treatment and appropriate supportive care should be provided for diarrhea, including use of antidiarrheal drugs according to standard operation of the study institution. Antidiarrheal drugs cannot be taken as prophylactic drugs. Patients should be instructed to start antidiarrheal drugs when the following earliest symptoms appear:

   - Shapeless or soft striped stool
   - More frequent bowel movements within one day than usual or
   - Abnormal high feces level

   If blood or mucus is present in the stool or if diarrhea is accompanied by fever, administration of antidiarrheal drugs should be delayed. In such cases, appropriate microbiological specimens should be obtained to diagnose and exclude infectious pathogens. Patients should also be advised to drink clean fluid as far as possible to prevent dehydration.

(2) Nausea/vomiting: Nausea and vomiting should be actively treated with antiemetics according to standard operation of the study institution. Patients should be encouraged to adhere to intake of plenty of oral fluid.

(3) Anemia: Anemia may be treated with blood transfusion and/or erythropoietin as clinically indicated, but which should be clearly indicated and filled in the CRF as a concomitant medication. Colony stimulating factor is prohibited in Cycle 1 of this study.

(4) Neutropenia: The neutrophil colony stimulating factors, including G-CSF, PEG-G-CSF, or GM-CSF are used in Cycle 1 of the study.

(5) Thrombocytopenia: If clinically indicated, platelets can be transfused.

(6) Dyspepsia, gastroesophageal reflux disease, peptic ulcer disease, or other gastric acid-related conditions: treatment may be provided after consultation with the sponsor if clinically indicated.

9.6 Overdose

9.6.1 Definition of overdose

It is unknown if there is a potential of abuse of HQP1351.

An overdose refers to an excessive dose of a drug that is taken intentionally or unintentionally. No specific information suggests that overdose of HQP1351 can be used during treatment. There is no specific antidote for HQP1351 overdose.

The pharmacological effects may also be persistent for a long time after serum levels of active HQP1351 are no longer present. It is unknown if dialysis is permitted for patients using HQP1351.

In case of overdose of HQP1351, stop use immediately and closely observe the subject for signs of toxicity. If clinically indicated, appropriate supportive care should be provided.

9.6.2 Reporting drug overdoses to the sponsor

All overdose with or without an AE must be reported within 24 hours to the Sponsor.

9.7 Pregnancy

The risks of HQP1351 use during pregnancy have not been assessed. Patients who may become pregnant or whose partners may become pregnant must use acceptable contraceptive methods. Female patients must have been more than 1 year after menopause or use an acceptable contraceptive method (barrier method in combination with spermicide, oral contraceptives, licensed contraceptive implants, injectable long-acting contraceptives, intrauterine devices, tubal ligation) during the study period and within 3 months after the EOT.

9.7.1 Period of pregnancy information collection

Any pregnancies that occur from the first dose of the study product to 30 days after the last dose will be reported in the appropriate form. If a pregnancy is determined more than 30 days after the last dose, the investigator may report it based on his/her clinical judgment.

9.7.2 Measures to be taken if pregnancy occurs

If a female patient becomes pregnant while participating in the study, the investigator must be notified immediately, and recommend that she immediately discontinue the study treatment. Pregnancy information should be collected and recorded in an appropriate form and submitted to the sponsor’s pharmacovigilance team by the investigator within 24 hours of learning of the patient’s pregnancy. The investigator should consult with the patient to discuss the risks of continuing the pregnancy and the possible effects on the fetus, and if the patient insists on continuing the pregnancy, the patient and the fetus should continue to be monitored until the outcome of the pregnancy is determined. Maternal and infant information should all be provided to the sponsor. In general, the follow-up will end within 6-8 weeks after the expected date of delivery. Any early termination of pregnancy should be reported.

Pregnancy itself is not regarded as an AE unless the study drug may have interfered with the effectiveness of a contraceptive medication. Extrauterine pregnancy and pregnancy resulting in congenital anomalies/birth defects, spontaneous abortion or severe complications will be reported as SAEs.

9.7.3 Measures to be taken if a male patient's female partner becomes pregnant

The investigator will attempt to collect pregnancy information of any pregnant female partner during male subject's participation in this study. The pregnancy should be recorded in an appropriate form and submitted to the sponsor by the investigator within 24 hours of learning of the partner's pregnancy. The partner will be followed up to determine the outcome of the pregnancy. Maternal and infant information should all be provided to the sponsor. In general, the follow-up will end within 6-8 weeks after the expected date of delivery. Any early termination of pregnancy should be reported.

9.7.4 Warnings and precautions for use

At the time the study protocol passes review, there is no evidence of other special warnings or precautions for use other than those covered in the Investigator's Brochure. Additional safety information collected between IB updates will be communicated in the form of an investigator circular. This information will be incorporated into the patient's ICF and, if needed, should be discussed with the patient during the study.

10 Statistical Analysis

10.1 Sample Size Calculation

In the HQP1351 SJ-0002 CML patient study, the MTD has been determined to be 50 mg. But starting from the 12 mg dose group, the drug has shown its efficacy. The study is being expanded at 30 mg, 40 mg and 50 mg to determine the dose groups with the best efficacy/safety ratio. According to the data of HQP1351 SJ-0002 CML patient study, this study has been modified accordingly to randomize subjects into the 30 mg, 40 mg and 50 mg dose groups at the same time, about 10 subjects each.

As of February 2021, HQP1351 has undergone a total of 9 clinical studies, and preliminary results from the Phase I and Phase II clinical trials that have been conducted show a favorable safety profile in the dose range of 50 mg QOD. HQP1351 (olverembatinib tablets) has been granted conditional marketing authorization from the National Medical Products Administration on November 24, 2021, with a recommended dose of 40 mg QOD. Based on the safety data from 9 clinical studies that are conducted with HQP1351 and the safety and efficacy data of 36 patients already enrolled in this study, 40 mg QOD is selected as the dosage for adult subjects after discussion between the investigator and the sponsor. After the randomization of subjects at 30 mg, 40 mg and 50 mg QOD, with about 10 subjects in each group, it is planned to enroll 10-20 subjects in the non-randomized part to further explore the safety and efficacy of the drug, and the final number of enrolled subjects will be determined after discussion between the investigator and the sponsor according to the situation.

10.2 Study Endpoints

10.2.1 Primary endpoints

Safety and tolerability assessments include AEs, SAEs, physical examination, vital signs, ECG, and clinical laboratory tests.

10.2.2 Secondary endpoints

Secondary endpoints include the PK profile of HQP1351 in vivo, and efficacy of HQP1351 in the treatment of gastrointestinal stromal tumors.

Definition of efficacy:

Objective response rate (ORR): Defined as the percentage of subjects with confirmed best overall response of complete response (CR) and partial response (PR) (evaluated as per RECIST v1.1 by the investigator).

- When calculating ORR, PR and CR confirmed prior to any other antitumor therapy should both be counted as responses, regardless of how many assessments are missing prior to response.
- According to RECIST v1.1, patients whose best overall response is “unknown” or “not evaluated” will be considered as non-responders for ORR estimation.

- Patients who develop PD and continue to receive the study treatment after PD will be considered to have PD at the time the progression is determined, and will be counted for PD and regarded as non-responders for ORR and other efficacy calculations.

Clinical benefit rate (CBR): Defined as the proportion of patients with a complete response (CR), partial response (PR), and stable disease (SD) for ≥ 16 weeks.

Duration of response (DOR): In patients with a response (PR or CR) (as per RECIST v1.1), DOR is defined as the time between the date of the first documented response (PR or CR) and the date of the first documented PD or the date of death from any cause. The censoring rules for DOR are the same as the following censoring rules for PFS.

Progression-free survival (PFS): Defined as the time between the date of the first dose of HQP1351 and the date of the first documented PD (as per RECIST v1.1) or the date of death from any cause.

Patients who do not have PD or death as of the analysis cutoff date, or who do not have PD when starting any further antitumor therapy, will be censored at the last adequate tumor assessment prior to the cutoff date, or prior to the date of antitumor therapy.

Overall survival (OS): Defined as the time interval from the first dose of HQP1351 to death due to any reason.

10.2.3 Exploratory endpoints

Mutations and/or expression profiles of KIT, PDGFRa or other and other tumor-associated genes associated with the efficacy of HQP1351.

10.3 Analysis Sets

Full analysis set (FAS)

The FAS includes patients who have received at least one dose of HQP1351 and have baseline tumor assessment data.

Per-protocol set (PPS)

The PPS consists of a subset of patients in the FAS who meet the requirements of the clinical study protocol. The PPS includes patients with adequate tumor assessment at baseline and follow-up tumor assessment > 2 weeks after initiation of treatment (unless PD has been previously observed), and no major protocol violations.

Patients with major protocol violations will be excluded from the PPS, and protocol violations leading to exclusion from the PPS will be defined in detail in the Statistical Analysis Plan.

Safety set

The SS will include all subjects who have received HQP1351 at least once.

Pharmacokinetic Analysis Set (PAS)

The PAS consists of all patients in the Full Analysis Set who have received the study treatment and provided evaluable PK data (without major deviations from clinical study protocol which are considered to significantly affect drug PK). The PAS will be used as an analysis set to report the PK data.

10.4 Statistical Analysis Method

At the end of the study, safety and efficacy data will be analyzed. After comprehensive evaluation and comparison of efficacy, safety and PK data, 1 dose will be selected for Phase II studies.

10.4.1 Demographic and baseline data

Demographic and baseline characteristics will be summarized and tabulated: continuous variables will be expressed with mean, median, standard deviation, minimum, and maximum; discrete variables will be expressed with counts and percentages. Baseline vital signs and abnormal values with clinical significance will be summarized.

10.4.2 Efficacy analyses

All efficacy evaluations (ORR, CBR, DOR, PFS, OS) will be analyzed according to the investigator's evaluation (in accordance with RECIST v1.1). The ORR for all responses [PR and CR] must be confirmed by a repeat assessment not less than 4 weeks and theoretically not later than 5 weeks after the response criteria are met for the first time. All efficacy analyses will be based on FAS, and efficacy analyses based on PPS will also be performed as supporting analysis data. All efficacy data will be listed.

The estimate of ORR and CBR will be calculated and 95% confidence interval (CI) will be provided based on an exact binomial distribution.

The Kaplan-Meier method will be used to estimate the median duration of response and median progression-free survival, and Kaplan-Meier curves will be plotted respectively.

10.4.3 Safety data

All AEs will be coded according to Medical Dictionary for Regulatory Activities (MedDRA). The frequency (number and percentage of subjects) of subjects experiencing one or more AEs will be calculated and summarized by System Organ Class (SOC) and Preferred Term (PT). All AEs, drug-related AEs (AEs assessed as related or possibly related), AEs and SAEs graded based on NCI CTCAE, and AEs resulting in study withdrawal will be classified and summarized.

AEs and outliers (patient's physical examination results, vital signs, and laboratory results) determined by the investigator to be clinically significant will be summarized and tabulated by dose group.

Deaths, SAEs, and AEs leading to discontinuation of HQP1351 treatment will be listed in detail.

10.4.4 PK analysis

Appropriate, standard nonlinear analysis software (e.g., Pharsight Corporation WinNonlin®) will be used to analyze PK parameters such as area under the blood concentration-time curve (AUC), peak blood concentration (C_{max}), half-life ($t_{1/2}$), and steady-state blood concentration (C_{ss}), etc.

11 Quality Control and Management

11.1 Quality Control and Assurance

The sponsor and the investigator should fulfill their respective responsibilities, and strictly comply with the clinical trial protocol; the sponsor should formulate and follow the standard operating procedures (SOPs), and ensure that all relevant personnel of all the procedures are in strict compliance with the requirements of GCP and SOP through monitoring, quality control inspection and auditing, cooperate closely, ensure that all the materials are scientific and reliable, and all activities are recorded.

11.2 Monitoring of Clinical Study

As per GCP, the CRA must have the direct access to the investigator's source documents to verify the consistency of the data recorded in the CRF.

CRA is responsible for regular monitoring of CRF during the study to verify protocol compliance as well as the integrity, consistency and accuracy of the data entered. The CRA should be able to access all patient records required to verify the CRF. The investigator should agree to cooperate with the CRA to ensure that all follow-up affairs identified during the monitoring visit are resolved.

During visit of study institution, the CRA will:

- Check the study progress;
- Review collected study data;
- Verify source documents;
- Identify and resolve any issues.

This is to verify that:

- The data are reliable, accurate and complete;
- Patients' safety, rights and benefits are protected;
- The study is conducted in compliance with the currently approved protocol (and any amendments), GCP, and all applicable regulatory requirements.

The investigator agrees to allow the CRA to have a direct access to all relevant documents and arranges the time of his/ her staff to discuss any issues related to the study identified by the CRA.

11.3 Quality Management Examinations

In order to ensure compliance with GCP and all applicable regulatory requirements, Guangzhou HealthQuest Pharma Inc. will perform quality control inspection and audit. Drug regulatory authorities may conduct inspection on the study. These QC/audits/inspections may occur at any time during the study or after the end of the study. If a QC, audit or inspection is

conducted, the investigator and the study institution will agree to give the QCs/auditors/inspectors direct access to all relevant documents, to arrange the time of these personnel, and to discuss with the QCs/auditors/inspectors any relevant issues identified by the examination.

12 Ethical Requirements

12.1 Independent Ethics Committee (IEC)

Prior to eligibility screening, clinical study protocols and ICFs must be submitted to the IEC for review and approval. The composition of the IEC is in line with the recommendations of the World Health Organization (WHO) and ICHE6 guidelines 23 for good clinical practice (see below).

No changes may be made to the study without IEC approval, except those necessary to eliminate immediate hazards to study subjects.

Report of study progress should be submitted as per IEC-specified frequency of annual/regular follow-up review during the clinical study.

Notice of the end of the study will be sent to the regulatory authority and the IEC within 90 days of the completion of the follow-up of the last subject. If the study ends early, the competent authorities in China should be notified within 15 days, including the reasons for the early termination. A summary report of the study results must be sent to the competent authorities and the IEC within 1 year after the study ends.

12.2 Ethical Conduct of the Study

The study will be conducted in accordance with the ethical principles of the Declaration of Helsinki (adopted by the World Medical Association (WMA) at its 18th General Assembly in Helsinki, Finland in June 1964) and its subsequent amendments.

The study also complies with the ICH E6 guidelines for good clinical practice (CPMP/ICH/135/95).

The study will consider as fully as possible the guidelines adopted by the ICH and other relevant international guidelines, recommendations and requirements, provided that they do not violate Chinese laws.

The PI or his/her designated staff member will be responsible for the care of subjects during the study. If the PI is not at the clinical study site, he/she will leave instructions and a contact phone number for the staff.

The PI or his/her designated staff member will be responsible for the medical follow-up of subjects.

If a subject refuses to follow the PI’s instructions, the PI will not be liable for any legal responsibilities for that.

12.3 Written Informed Consent

All subjects will be informed orally and in writing of the objectives, procedures, and risks of participating in the study. Subjects must sign ICF in Chinese prior to any study-specific procedures. The ICF contains information on the objectives of the study, the procedures

followed in the study, and the risks and limitations of the study, and particularly mentions possible side effects of the drug and potential interactions. In addition, it also explains the insurance coverage provided during the study. The elements covered in the ICF have been prepared in accordance with China's GCP and ICH E6 guidelines (CPMP/ICH/135/95).

13 Data Processing and Document Retention

13.1 Case Report Form (CRF)

The PI-authorized personnel will enter the data into the Case Report Forms (CRFs) as required by the protocol. The PI is responsible for ensuring that the data entered into the CRF is complete and accurate, and for entering and updating data in a timely manner. In accordance with the ICH Good Clinical Practice (ICH-GCP) that is widely used internationally, the clinical research associate (CRA) has direct access to the investigator's data records in order to verify the consistency of the data in the CRFs with the original data.

The CRA must periodically review the CRFs to ensure that the data in the CRFs are consistent with the protocol, check the integrity and authenticity of the records, and guide the PI-authorized personnel to make any required corrections or supplements. The PI and his/her authorized personnel should cooperate with the CRA to ensure the successful completion of the clinical trial.

During the study, PK and exploratory biomarker samples (blood) will be collected/gathered from the study site and analyzed by the sponsor or its authorized central laboratory. Relevant information about PK and exploratory biomarker sample collection will be entered into the CRF and two laboratory application forms by a person authorized by the PI. One copy of the application form will be forwarded to the central laboratory along with the corresponding sample and required information (including study number, subject ID, etc.), and the other copy will be retained at the study site. The CRA will review the relevant CRF for accuracy and integrity and work with site staff to adjust any disagreements as needed. The CRA will also review the application form for integrity. The biological sample data obtained by the central laboratory will be forwarded to Guangzhou HealthQuest Pharma Inc. or its designated database (or authorized CRO) as third-party data.

At the same time, during study site visits, the tasks of the CRA include verifying the progress of the study, reviewing the collected study data, verifying the documentation of the study site, and answering questions and resolving problems that arise. These efforts are aimed to ensure the authenticity, accuracy and integrity of data, protect the safety and rights of patients, and ensure that the study is conducted in accordance with the previously approved protocol and the GCP requirements.

13.2 Data Collection

During each study visit, the participating physician will make progress notes in the subject's medical record to document any significant observations. These records will contain at a minimum:

- The date of the visit and the corresponding date or visit in the study plan (e.g. screening, Day 1, Day 15, etc.).
- General condition and patient status notes, including significant medical findings. The

severity, frequency, duration, and resolution of any reported AE, and the investigator's assessment of whether the reported AE is related to the study drug.

- Concomitant medications or dose changes.
- The main reference for the completed procedures.
- The medical record (progress note) contains the signatures or initials of all the doctors who have made the record.

In addition, any contact that provides significant clinical information to the subject by telephone or other means should also be recorded in the medical record (progress note) as described above.

Information in medical records (progress notes) and other original documents will be transcribed in a timely manner to the appropriate sections of the CRF.

Changes to information in medical records (progress notes), CRFs and other original documents will be signed and dated by the investigator or his/her designee on the day when the change is made. If the reason for the change is not obvious, a brief note should be written next to the change.

13.3 Preservation of Original Documents

The original documents contain the original observation findings and all activities of the clinical study. The original documents include, but not limited to medical records (progress note), computer printouts, screening logs, and data recorded by automated instruments.

All original documents in this study will be maintained by the investigator and will be available for inspection by authorized persons. The original ICF signed by each subject will be kept on file with the records maintained by the investigator, copies of which will be provided to the subject.

13.4 Record Retention

All data obtained from the study will be considered as the assets of Guangzhou HealthQuest Pharma Inc.

Records should be maintained in accordance with the current GCP guidelines. All documents required for the study (including subject records, original documents, CRF, and study drug dispensing and return forms) must be kept on file. Relevant documents shall be retained for a longer period however if required by applicable regulatory requirements or by special regulations of the sponsor. The sponsor will be responsible for informing investigators of the specific retention duration of these documents.

The investigator will not dispose of any records related to this study without the sponsor's written permission, and will provide the sponsor with the opportunity to collect such records. The investigator must be responsible for maintaining the adequacy and accuracy of the original

paper documents of all observation reports and data obtained in this study. These documents will be subject to examination and inspection by the sponsor, its representatives and relevant regulatory authorities at any time.

If the investigator has position changes, withdraws from the study, or retires, the responsibility for maintaining the records will be transferred to other study staff who will shoulder the secondary responsibility. Such transfer must be notified to and approved by the sponsor.

14 Responsibilities of All Parties and Information Disclosure

14.1 Duties of Each Party

The sponsor, investigators, study responsible institution and participating institutions should fulfill corresponding responsibilities in accordance with *Good Clinical Practice* as well as this protocol.

14.2 Information Disclosure

14.2.1 Ownership

All the information provided by Guangzhou HealthQuest Pharma Inc. and the data and information generated as part of the study (excluding subjects' medical records) are the exclusive property of Guangzhou HealthQuest Pharma Inc.

14.2.2 Confidentiality

All the information provided by Guangzhou HealthQuest Pharma Inc. and the data and information generated as part of the study (excluding subjects' medical records) will be kept confidential by investigators and other site staff. The information related to this study is subject to the confidentiality provisions of the Clinical Study Agreement between institution and Guangzhou HealthQuest Pharma Inc.

14.2.3 Publication

Any rights of publication and representation of findings relevant to the clinical study under this protocol is subject to the terms of the Clinical Study Agreement between institution and Guangzhou HealthQuest Pharma Inc.

15 Procedure

15.1 Regulatory Approval

According to local national regulations, Guangzhou HealthQuest Pharma Inc. is responsible for obtaining the relevant regulatory approvals.

Subjects shall not participate in the study until relevant regulatory approvals are obtained. Approved copies (if available in accordance with local national regulations) will be provided to the investigator and the IEC.

15.2 Protocol Amendment

In accordance with ICH GCP E6 (R2) guidelines, investigators shall not deviate from or alter the protocol prior to receiving the sponsor's approval and the written approval of the IEC on protocol amendment, except for protocol amendment necessary for immediately eliminating current injury to subjects of the study, or logistic or administrative changes involved the study (e.g. changes in CRAs and telephone number).

Any changes to the protocol shall be handled in accordance with protocol amendment. Any potential revision shall be approved by the sponsor. Written revisions must be submitted to relevant regulatory departments and the competent IEC. Investigators may not implement relevant changes prior to obtaining the approval of IEC on protocol amendment, except for vitally necessary changes for immediately eliminating obvious current injury to subjects , which shall be notified to IEC within 5 days after carrying out changes.

All protocol amendments must be approved in writing by relevant regulatory institutions and IEC except for administrative amendments which only require a notification other than a written approval. Protocol amendment and guidance for adding amendment to the protocol will be sent to all receptors of the original protocol after being approved.

If protocol amendments, at the discretion of local IEC, investigator and / or the sponsor, have changed the study design, procedures and / or increased potential risks to subjects, an approved written ICF will be revised. The revised ICF shall be audited and approved by the sponsor, relevant regulatory institutions and IEC. In such case, informed consent of subjects enrolled into the study shall be obtained again prior to continuing the study.

15.3 Compliance with and Deviation from the Protocol

It is necessary to read through the whole study protocol and follow the instructions, except for emergency conditions requiring immediate intervention for subjects' protection, safety and health at the discretion of the investigator or a reliable, trained and professional personnel (sub-investigator) designated by the investigator.

However, the investigator or a designated staff must communicate any major protocol deviation due to an emergency, accident or error to the medical monitor as soon as possible by telephone. As a result, they can make a decision as soon as possible on whether or not to continue the

study in the subject. The decision will be recorded by the investigator, sponsor and medical monitor.

15.4 Policy for Paper Publication

Upon study completion, the study results will be co-submitted by the investigators and sponsor for publication. The investigator must undertake not to submit any part of the protocol data for publication without the prior consent of Guangzhou HealthQuest Pharma Inc.

15.5 Clinical Study Report

A final clinical study report (CSR) will be made in accordance with the principles on the structure and content of clinical study reports in ICH GCP. A final CSR will be made regardless of whether this study is completed or early terminated. The sponsor will provide a copy of the final CSR to all investigators for reference.

15.6 Contract and Financial Details

The investigator (and/or, relevant hospital administrative representatives) and the sponsor will sign a clinical study agreement prior to the start of the study to outline all responsibilities of the sponsor and the investigator related to the study. The contract should specify the method of payment (direct or indirect) for the costs of drugs, laboratories and other services necessary for the protocol.

15.7 Insurance, Indemnity and Compensation

Guangzhou HealthQuest Pharma Inc. undertakes the relevant clinical research insurance policy.

Guangzhou HealthQuest Pharma Inc. has provided insurance for the subjects participating in this study. If the subjects suffer related injuries due to their participation in this study or due to the investigational drug in this study, Guangzhou HealthQuest Pharma Inc. will bear the cost of treatment and corresponding financial compensation for subject's injury or death related to the trial in accordance with the provisions of China's *Good Clinical Practice* and relevant laws and regulations.

15.8 Termination of the Study

The sponsor can terminate the study. To ensure subjects' best interests and medical and ethical considerations, premature termination of the study can be conducted after receiving the approval of investigators and the sponsor. When terminating the study, Guangzhou HealthQuest Pharma Inc. and the investigator will ensure that the protection of subjects' rights and interests has been fully considered.

15.9 Management of Study Site Documents

The investigator is responsible for ensuring the preservation of the study site documents. Study site documents will include but are not limited to the following information:

- Investigator’s Brochure;
- The currently signed version of the protocol and any previous versions of the protocol;
- Amendments to the protocol (if applicable);
- Operation manual (if applicable);
- The current informed consent form (blank) and any previous version of the informed consent form;
- CVs of investigators and sub-investigators and copies of their respective licenses (if required by law); investigators are also required to complete all regulatory documents required by the ICH GCP and local or national regulations;
- EC approval of the protocol, informed consent form, and documentation of any amendments to the protocol and any amendments to the informed consent form;
- All necessary correspondences among the investigator, the EC and the sponsor/CRO relating to the execution of the study;
- Laboratory certificates;
- Monitoring log;
- Study drug dispensing sheets;
- Complete all signature forms for investigator duties in the CRF;
- Complete all staff signature forms summarized for the study drug responsibilities;
- Signed subject's ICF;
- Chromatograms;
- Clinical trial records;
- Data and statistical processing data;
- Scientific research record book;
- All other relevant materials related to the trial.

16 Expected Progress

Each study site will complete the clinical trial within 4-5 years after the study documents, investigational drug and trial fund are in place.

17 Reference

1. Hirota S, Isozaki K, Moriyama Y, et al. Gain-of-function mutations of c-kit in human gastrointestinal stromal tumors. Science 1998; 279:577-580.
2. Joensuu H, Hohenberger P, Corless C L. Gastrointestinal stromal tumour[J]. Lancet 2013; 382:973-83.
3. Joensuu H, Vehtari A, Riihim€aki J, et al. Risk of recurrence of gastrointestinal stromal tumour after surgery: an analysis of pooled population-based cohorts. Lancet Oncol 2012;13:265-74.
4. Søreide K, Sandvik OM, Søreide JA, Giljaca V, Jureckova A, Bulusu VR. Global epidemiology of gastrointestinal stromal tumours (GIST): A systematic review of population-based cohort studies. Cancer Epidemiol. 2016 Feb;40:39-46.
5. Miettinen M, Lasota J. Gastrointestinal stromal tumors: pathology and prognosis at different sites. Semin Diagn Pathol 2006;23:70-83.
6. Dematteo RP, Lewis JJ, Leung D, Mudan SS, Woodruff JM, Brennan MF. Two hundred gastrointestinal stromal tumors: recurrence patterns and prognostic factors for survival. Ann Surg. 2000; 231(1):51–8
7. Demetri GD, von Mehren M, Blanke CD, et al. Efficacy and safety of imatinib mesylate in advanced gastrointestinal stromal tumors. N Engl J Med 2002;347:472-480.
8. Demetri GD, van Oosterom AT, Garrett CR, et al. Efficacy and safety of sunitinib in patients with advanced gastrointestinal stromal tumour after failure of imatinib: a randomised controlled trial. Lancet 2006;368:1329-1338.
9. Demetri GD, Reichardt P, Kang YK, et al. Efficacy and safety of regorafenib for advanced gastrointestinal stromal tumours after failure of imatinib and sunitinib (GRID): an international, multicentre, randomised, placebo-controlled, phase 3 trial. Lancet 2013;381:295-302.
10. NCCN clinical practice guidelines in Oncology:Soft Tissue Sarcoma（Version 2.2017）.
11. Michael C. Heinrich, Margaret von Mehren, George D, et al. Ponatinib efficacy and safety in patients (pts) with advanced gastrointestinal stromal tumors (GIST) after tyrosine kinase inhibitor (TKI) failure Results from a phase 2 study. J Clin Oncol 33, 2015 (suppl; abstr 10535).
12. Eisenhauer EA1, Therasse P, Bogaerts J, et al. New response evaluation criteria in solid tumours: revised RECIST guideline (version 1.1).Eur J Cancer. 2009 Jan;45(2):228-47.
13. Li J, Ye Y, Wang J, et al. Chinese consensus guidelines for diagnosis and management of gastrointestinal stromal tumor. Chin J Cancer Res. 2017 Aug; 29(4): 281-293.

18 Appendices

Appendix 1: National Cancer Institute-Common Terminology Criteria for Adverse Events v4.03 (NCI CTCAE v4.03)

NCI CTCAE v4.03 is available at the following website:

https://evs.nci.nih.gov/ftp1/CTCAE/About.html

Spiral-bound NCI CTCAE v4.03 is available at written request:

Cancer Therapy Evaluation Program

9000 Rockville Pike

Executive Plaza North (mail stop)

Bethesda, MD 20892

For special consultation of common toxicity criteria, please send an email to: ncictephelp@ctep.nci.nih.gov

Appendix 2: ECOG performance status score

ECOG PS Score*

**Classification**	**ECOG**
0	Fully active, able to carry on all pre-disease performance without restriction.
1	Restricted in physically strenuous activity but ambulatory and able to carry out work of a light or sedentary nature; for example, light housework, office work.
2	Ambulatory and capable of all selfcare but unable to complete any work activities. up and about more than 50% of waking hours.
3	Capable of only limited selfcare; confined to bed or chair more than 50% of waking hours.
4	Completely disabled, completely incapable of self-caring totally confined to bed or chair.
5	Dead

* Oken,M.M, Creech, R.H, Tormey, D.C, Horton, J., Davis, T.E, McFadden, E.T., Carbone, P.P. Toxicity And Response Criteria Of The Eastern Cooperative Oncology Group. Am J Clin Oncol 5:649-655, 1982.

Appendix 3: Cockcroft-Gault Formula for Calculation of Creatinine Clearance

Female:

$$\text{CrCI (mL/min)} = 0.85 \times \frac{[140 - \text{age (years)}] \times \text{weight (kg)}}{0.818 \times \text{serum creatinine } (\mu\text{mol/L})}$$

Male:

$$\text{CrCI (mL/min)} = \frac{[140 - \text{age (years)}] \times \text{weight (kg)}}{0.818 \times \text{serum creatinine } (\mu\text{mol/L})}$$

Appendix 4: ECG QTcB Interval Calculation (Bazett's Formula)

QTcB=QT/(RR^0.5), RR is a standardized heart rate, obtained by dividing 60 by heart rate.
