# Supplementary material for: Olverembatinib, a multikinase inhibitor that modulates lipid metabolism, in advanced succinate dehydrogenase-deficient gastrointestinal stromal tumors: a phase 1b study and translational research
Source: Signal Transduct Target Ther. 2025 Nov 4;10:361. doi: 10.1038/s41392-025-02456-9 (PMC12583704; doi:10.1038/s41392-025-02456-9)

Supplementary Materials for

Olverembatinib, a multikinase inhibitor that modulates lipid metabolism, in advanced succinate dehydrogenase-deficient gastrointestinal stromal tumors:
A phase 1b study and translational research

Hai-Bo Qiu, Zhiyan Liang, Jing Yang, Ye Zhou, Zhi-Wei Zhou, Xiang-Bin Wan, Ning Li, Kai-Xiong Tao, Yong Li, Xin Wu, Chen Yang, Zi Chen, Hengbang Wang, Lichuang Men, Yan Xiong, Lihui Liu, Dajun Yang, Yifan Zhai, and Rui-Hua Xu

Correspondence to: [dyang@ascentage.com](mailto:dyang@ascentage.com), [yzhai@ascentage.com](mailto:yzhai@ascentage.com), [xurh@sysucc.org.cn](mailto:xurh@sysucc.org.cn)

**This PDF file includes:**

Original films of western blots


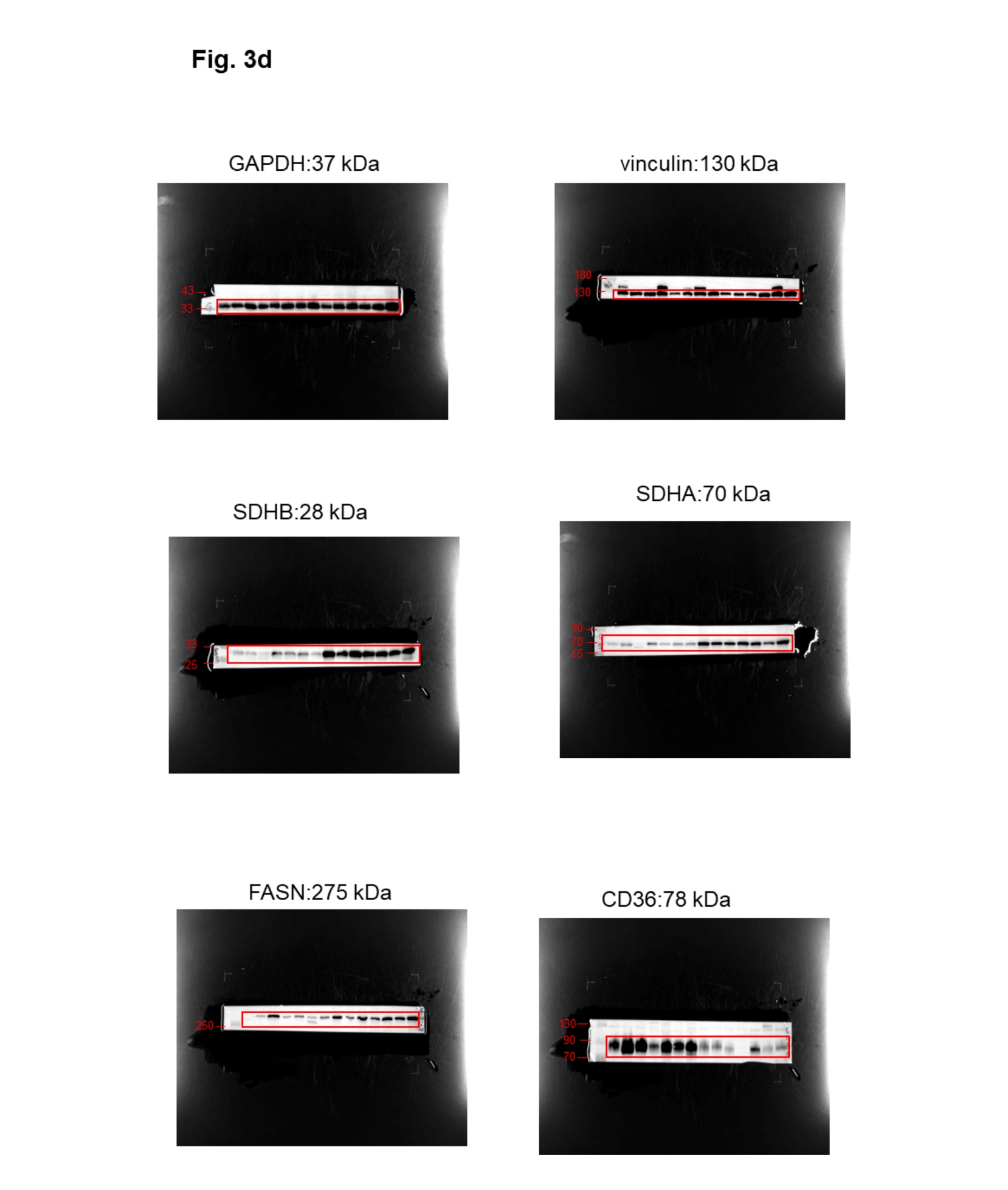


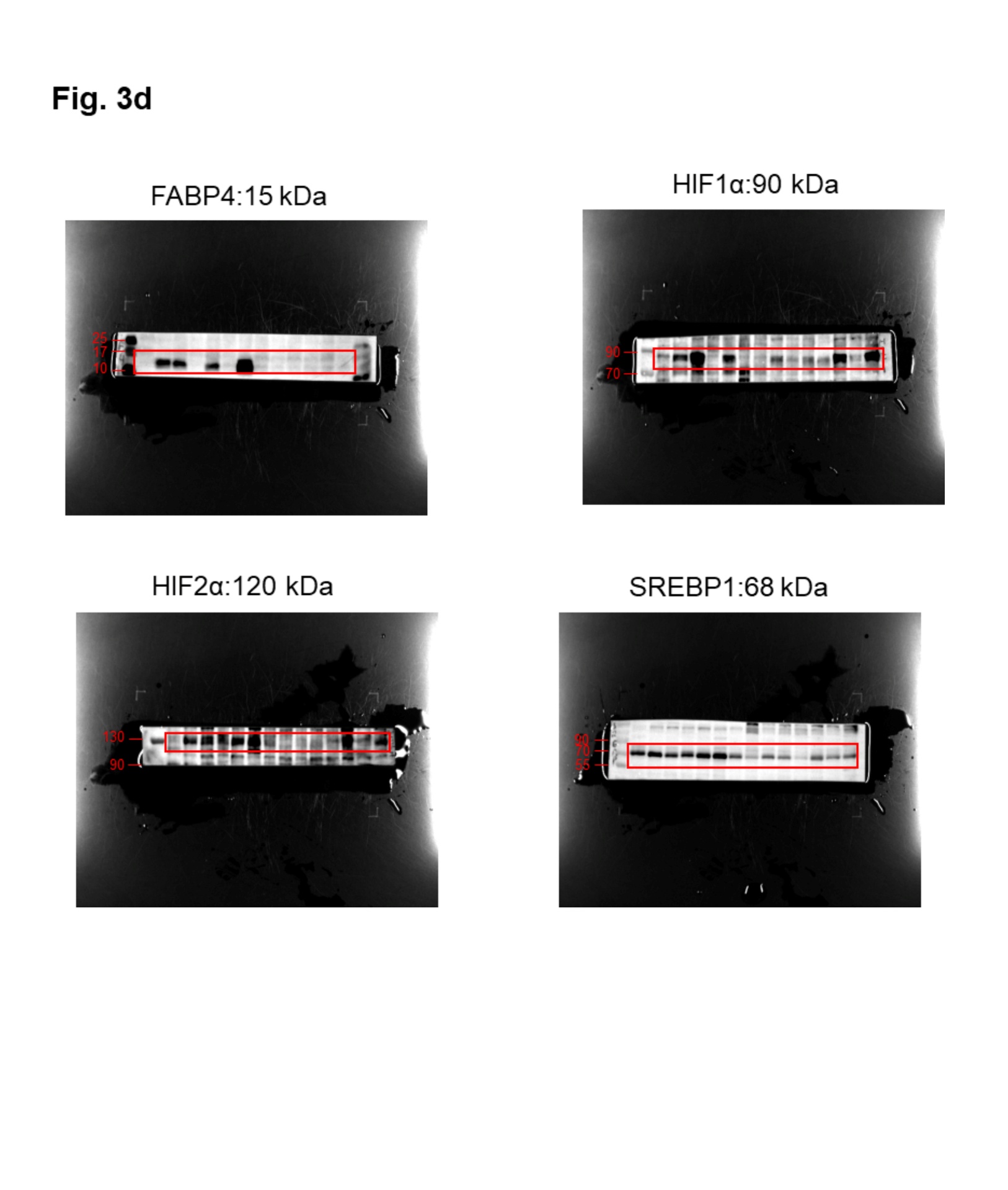


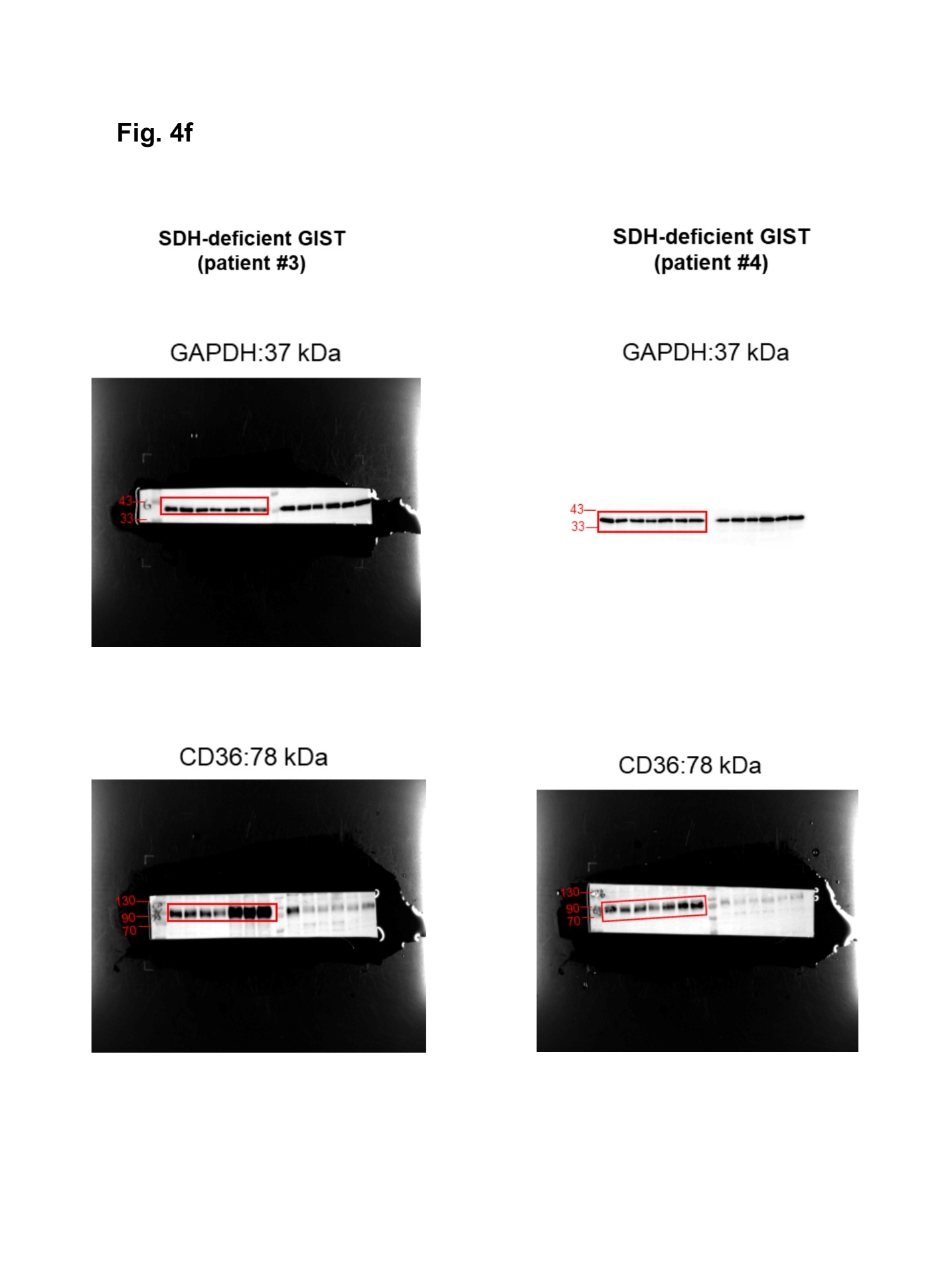


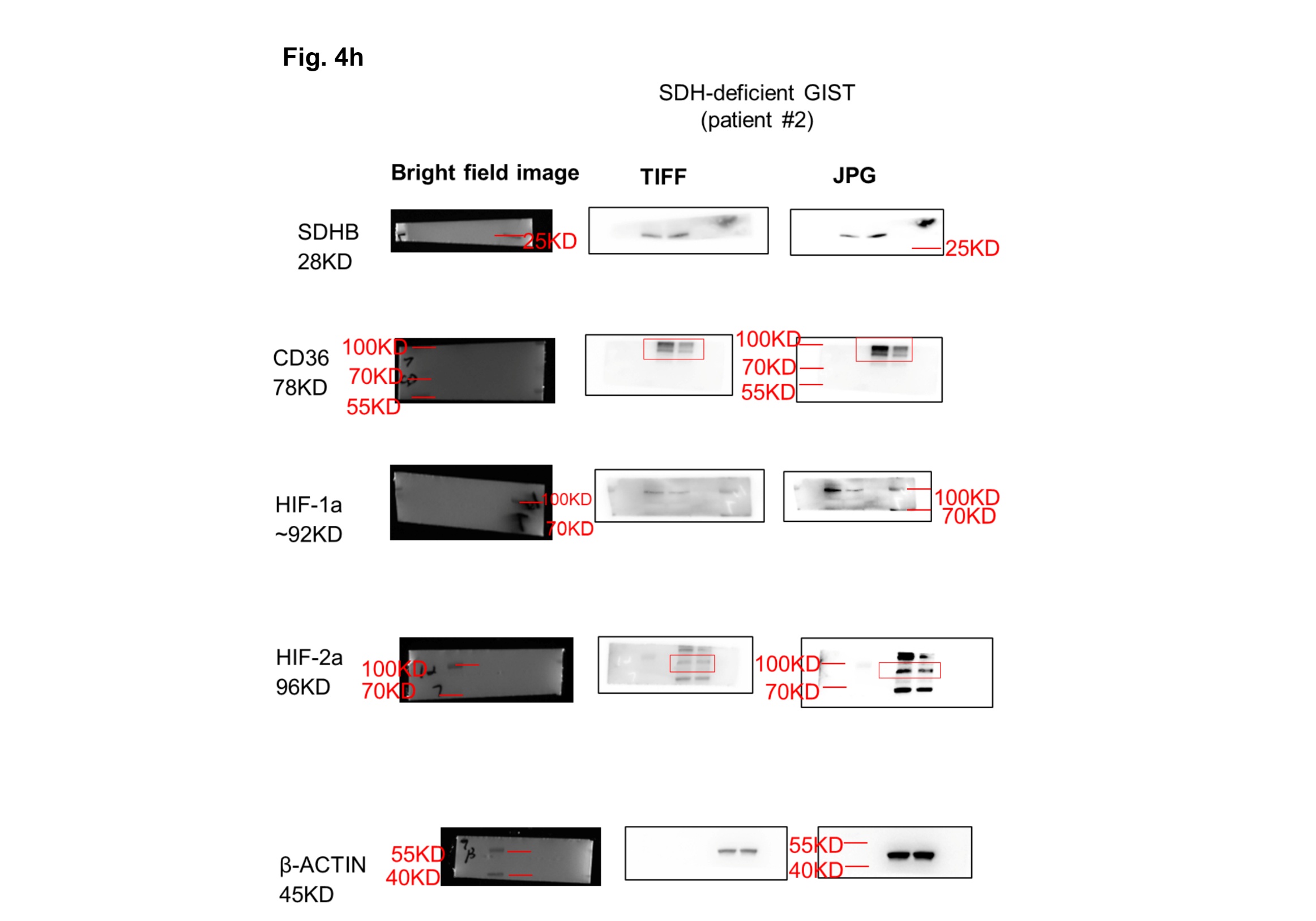


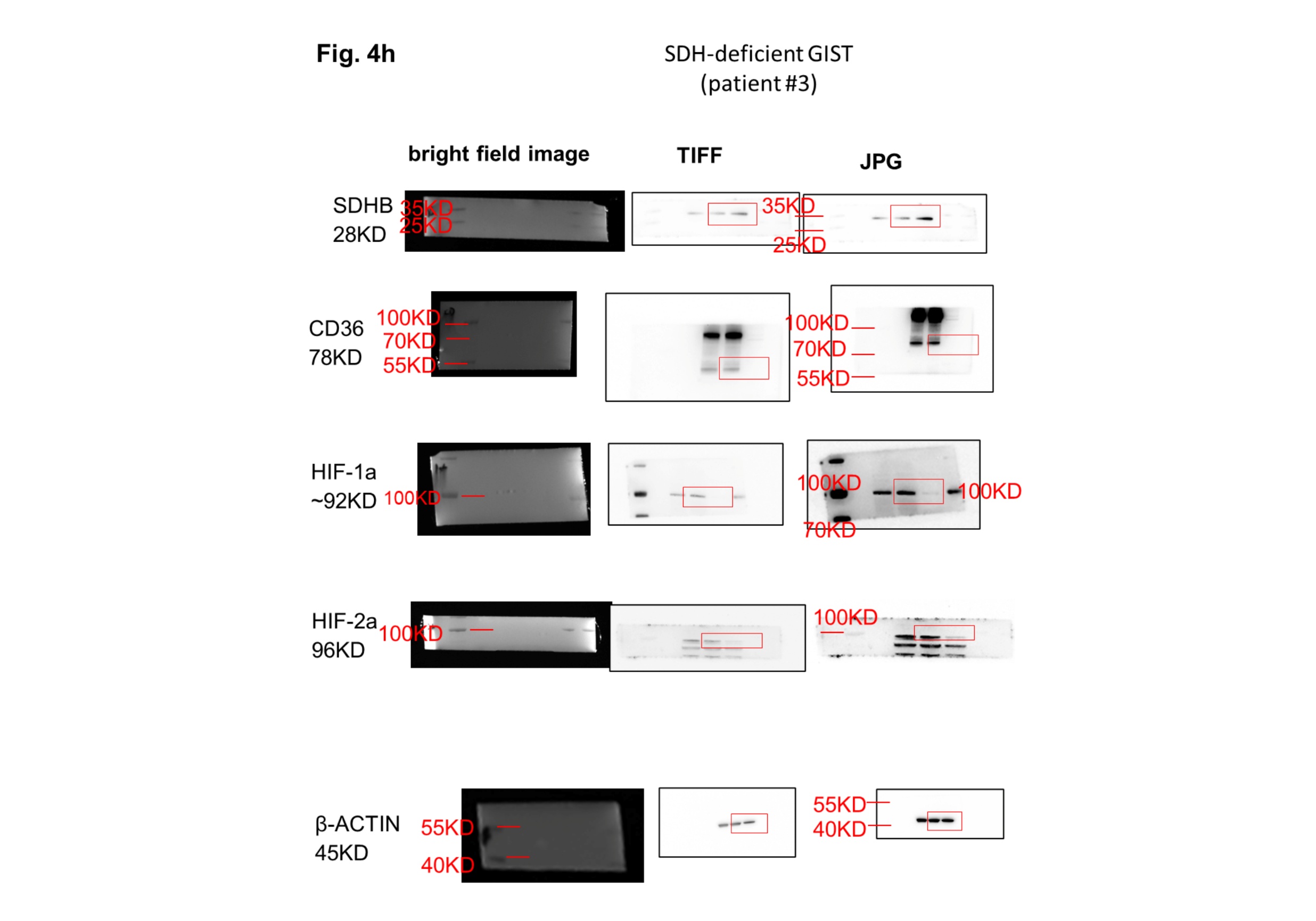


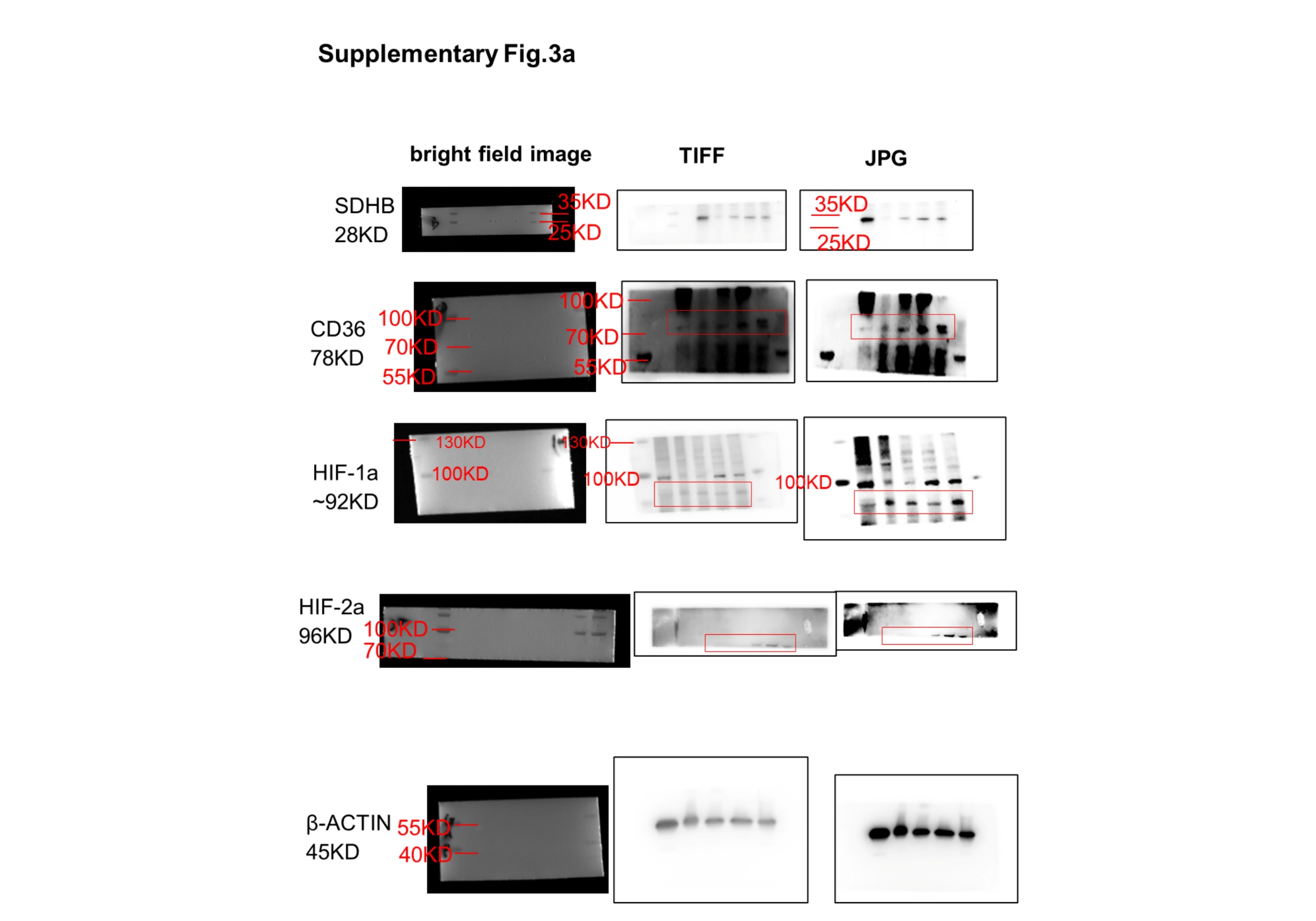


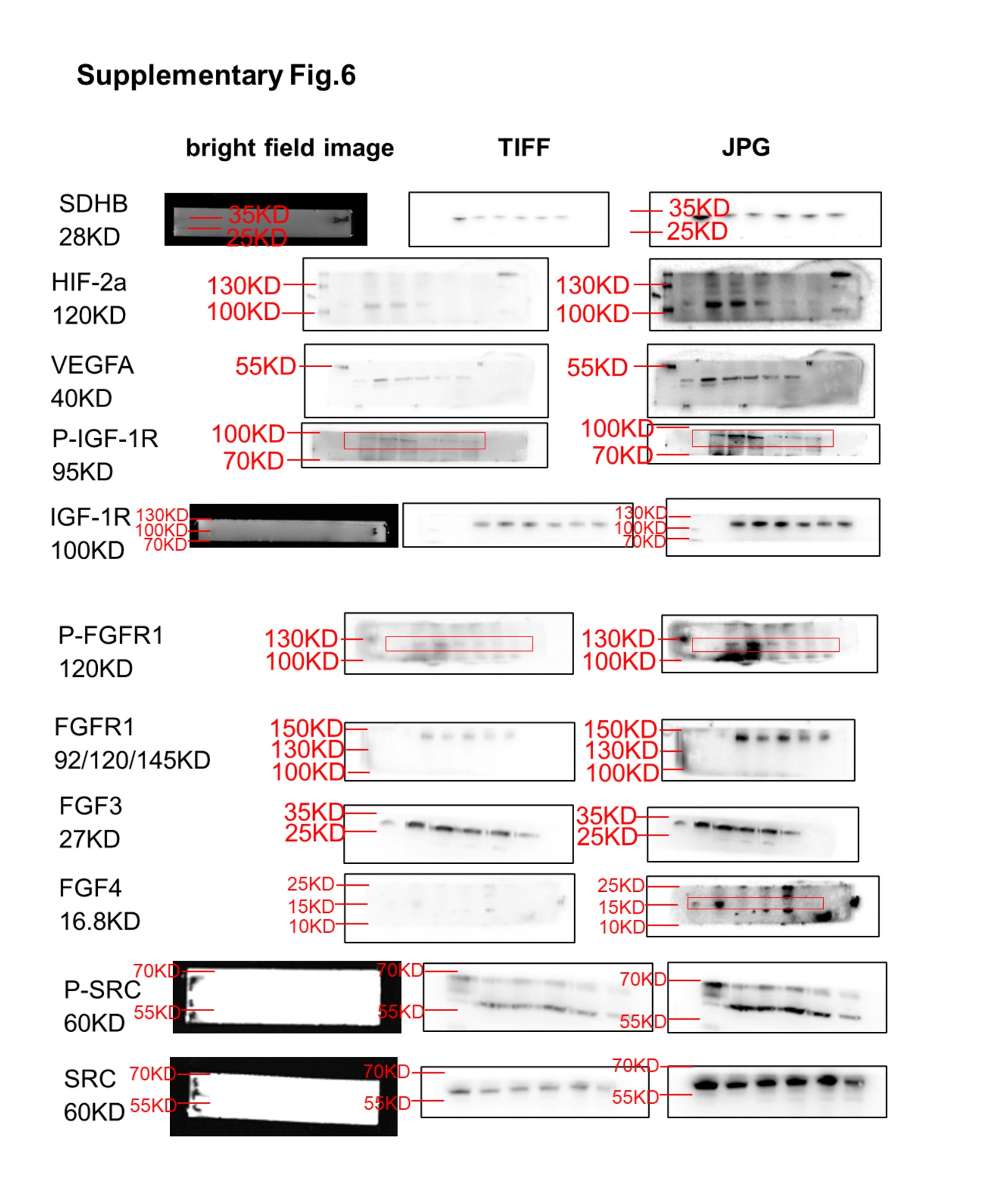


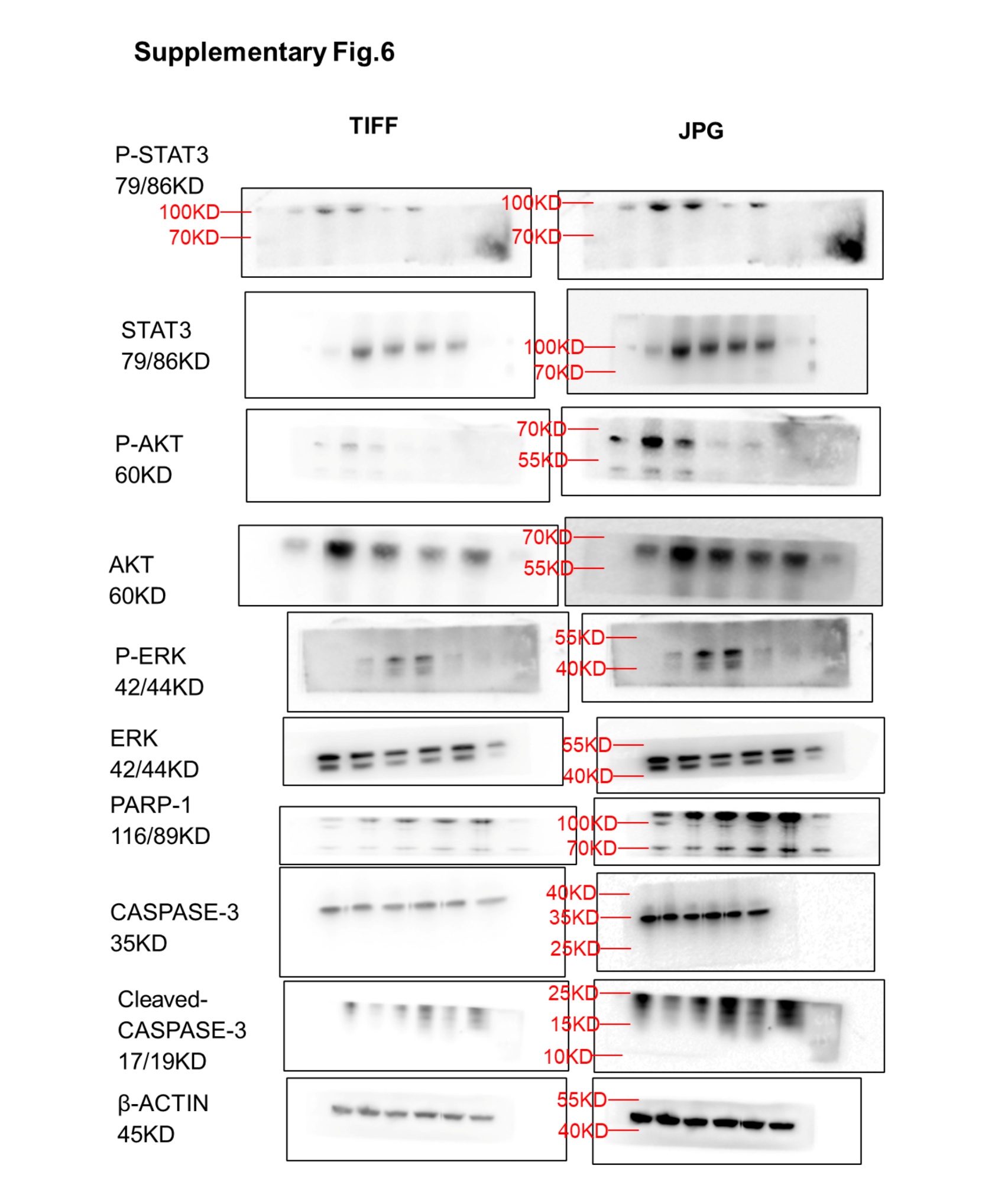

Supplement: Supplementary file 10 — Original films of western blots [file 41392_2025_2456_MOESM10_ESM.docx]
